# Groovy and Gnarly: Surface Wrinkles as a Multifunctional Motif for Terrestrial and Marine Environments

**DOI:** 10.1093/icb/icac079

**Published:** 2022-06-08

**Authors:** Venkata A Surapaneni, Mike Schindler, Ricardo Ziege, Luciano C de Faria, Jan Wölfer, Cécile M Bidan, Frederik H Mollen, Shahrouz Amini, Sean Hanna, Mason N Dean

**Affiliations:** City University of Hong Kong, 31 To Yuen Street, Kowloon, Hong Kong; Max Planck Institute of Colloids and Interfaces, Am Mühlenberg 1, Potsdam, Brandenburg 14476, Germany; City University of Hong Kong, 31 To Yuen Street, Kowloon, Hong Kong; Max Planck Institute of Colloids and Interfaces, Am Mühlenberg 1, Potsdam, Brandenburg 14476, Germany; University College London, 14 Upper Woburn Place, London WC1H 0NN, UK; Humboldt University of Berlin, Unter den Linden 6, Berlin 10099, Germany; Max Planck Institute of Colloids and Interfaces, Am Mühlenberg 1, Potsdam, Brandenburg 14476, Germany; Elasmobranch Research Belgium, Rehaegenstraat 4, 2820 Bonheiden, Belgium; Max Planck Institute of Colloids and Interfaces, Am Mühlenberg 1, Potsdam, Brandenburg 14476, Germany; University College London, 14 Upper Woburn Place, London WC1H 0NN, UK; City University of Hong Kong, 31 To Yuen Street, Kowloon, Hong Kong; Max Planck Institute of Colloids and Interfaces, Am Mühlenberg 1, Potsdam, Brandenburg 14476, Germany

## Abstract

From large ventral pleats of humpback whales to nanoscale ridges on flower petals, wrinkled structures are omnipresent, multifunctional, and found at hugely diverse scales. Depending on the particulars of the biological system—its environment, morphology, and mechanical properties—wrinkles may control adhesion, friction, wetting, or drag; promote interfacial exchange; act as flow channels; or contribute to stretching, mechanical integrity, or structural color. Undulations on natural surfaces primarily arise from stress-induced instabilities of surface layers (e.g., buckling) during growth or aging. Variation in the material properties of surface layers and in the magnitude and orientation of intrinsic stresses during growth lead to a variety of wrinkling morphologies and patterns which, in turn, reflect the wide range of biophysical challenges wrinkled surfaces can solve. Therefore, investigating how surface wrinkles vary and are implemented across biological systems is key to understanding their structure–function relationships. In this work, we synthesize the literature in a metadata analysis of surface wrinkling in various terrestrial and marine organisms to review important morphological parameters and classify functional aspects of surface wrinkles in relation to the size and ecology of organisms. Building on our previous and current experimental studies, we explore case studies on nano/micro-scale wrinkles in biofilms, plant surfaces, and basking shark filter structures to compare developmental and structure-vs-function aspects of wrinkles with vastly different size scales and environmental demands. In doing this and by contrasting wrinkle development in soft and hard biological systems, we provide a template of structure–function relationships of biological surface wrinkles and an outlook for functionalized wrinkled biomimetic surfaces.

## Introduction

From large ventral pleats on the throats of humpback whales to fine scale ridges on flower petals, a variety of wrinkle-like surface patterns exist at highly diverse length scales in nature. In addition to the environment and species involved, the location, size, and morphological complexity of surface wrinkles determine their function and ecological role. Therefore, understanding the functional morphology of surface wrinkles across biological systems will not only assist in establishing organismal structure–environment links, but also in laying out morphological rules for diverse technical applications.

In this work, we explore the structure–function relationships of surface wrinkles from various terrestrial and aquatic organisms from an ecomorphological viewpoint. First, we briefly describe the mechanical aspects of wrinkle formation, distinguishing between soft and hard biological systems (made of materials that yield and resist deformation, respectively). We then investigate structure–function aspects of biological wrinkled surfaces in relation to the size and ecology of organisms in a broad-scale metadata analysis of the literature. To illustrate the diverse interrelation of formation, development, and function in wrinkled biological systems, we then discuss three case studies based on our previous work on plant surfaces and preliminary data from our ongoing work on bacterial biofilms and basking shark filter structures. Taken together, we outline structure–function rules for biological surface wrinkles and provide an outlook for the targeted design of functionalized biomimetic surfaces (Fu et al. 2018; Zou et al. 2019; Bergmann et al. 2020).

## How wrinkles develop and vary across biological systems

### Basic mechanical aspects

**Fig. 1:**
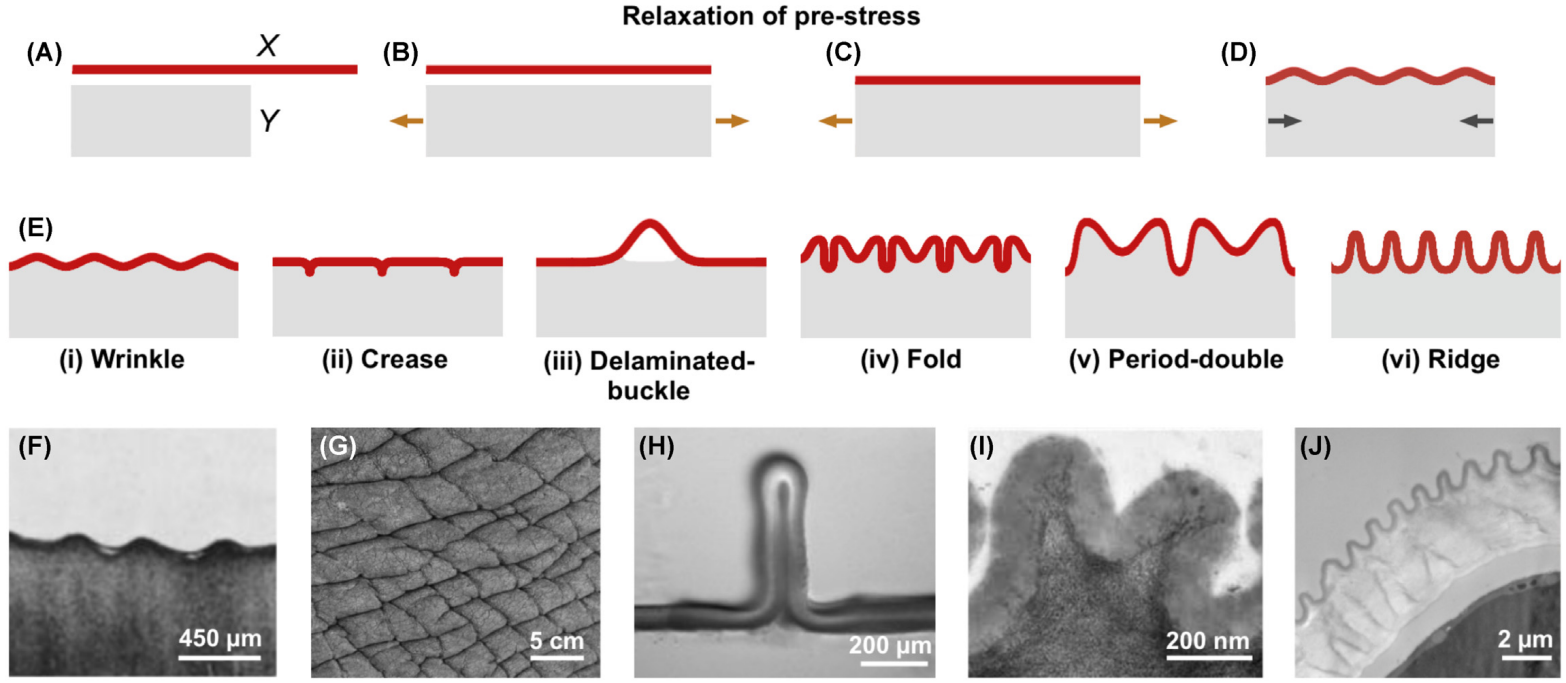
The schematic **(A–D)** shows the mechanics of wrinkle formation by the release of prestretch. The substrate is assumed to be stretched (in tension) initially from state A to B, and a thin top layer *X* is bonded to the substrate *Y* in C. Once the tension is released, the substrate tends to return to its original state leading to the formation of surface wrinkles. The schematic **[E(i–vi)]** shows different possible morphological patterns of wrinkles (adapted from Wang and Zhao 2015) and **(F–J)** show typical examples in biological systems that correspond to the patterns in E. **(F)** Skin wrinkles of a bottlenose dolphin *Tursiops truncatus* (modified from Shoemaker and Ridgway 1991with permission); **(G)** creases on an elephant skin; **(H)** delamination of a biofilm (modified from Ziege et al. 2021, CC BY 4.0); **(I)** period-double of cuticle on a *Narcissus pseudonarcissus* petal (modified from Jeffree 2006 with permission); and **(J)** cuticular ridges on a *Hibiscus trionum* petal (modified from Vignolini et al. 2015 with permission).

Aging human skin, a drying apple, and a moving earthworm, all share a distinctive surface feature: wrinkles form on the surface of their multi-layered tissues, arising from a compression-induced buckling instability (albeit from different sources; see following section). In any bilayer system with a thin outer layer and thick inner substrate, an in-plane strain mismatch between these components due to compressive forces may induce mechanical instabilities, leading to the formation of wrinkles. Out-of-plane deformations of the system arise when bending becomes energetically favorable compared to compression. The structural effects of this mismatch in strain can be intuitively understood by considering two fabrics, *X* and *Y* (Fig. 1A–D, thin red layer *X* and thick gray substrate *Y*), the latter pre-stretched before the fabrics are bonded together. In this case, wrinkles will automatically emerge upon release of the stress in material *X*; in fact, this process of “self-shaping” is currently of great interest to the textile industry for the formation of 3D surfaces (Kycia and Guiducci 2020). Alternatively, the stress-inducing out-of-plane buckling could be introduced after the materials are bonded (e.g., by growth, swelling or shrinking of one layer; see following section).

In these structural formation processes, the wavelength and amplitude of the evolving wrinkles depend on the material properties and structural aspects of the components such as layer-to-layer rigidity ratio and layer thickness (Cerda and Mahadevan 2003; Ma et al. 2020). For example, a thin, rigid layer supported by a soft substrate (e.g., human skin) acquires a wrinkle wavelength proportional to its layer thickness, whereas the wavelength of a soft layer supported by a rigid substrate (e.g., gut) follows a more complex relation (Li et al. 2012). When confinement leads to larger compressive stresses (e.g., as with rapid anisotropic growth of cuticle relative to epidermal cell wall in plants), wrinkles can evolve (Fig. 1E–J) towards folds, ridges, creases, or even delaminated buckling (creating pockets between layers), depending on the bending energy of the film, the viscoelastic properties of the substrate and the interfacial energies (Brau et al. 2013). The extent and anisotropy of strain mismatch and the adhesion energy between the layers can also greatly influence morphological differences of the surface wrinkles and may lead to checkerboard, herringbone, or labyrinth-like spatial wrinkle patterns or delaminations (Huang et al. 2005; Wang and Zhao 2016). Wrinkles can also evolve in systems with more than two-layers as in multilayered and/or graded biological tissues, and then the wrinkle morphology depends on the structural and material properties of all layers and constituents involved and the nature of the interactions between them (Kruglikov and Scherer 2018).

### Soft biological systems

While biological tissues aren't the stretched and bonded textiles described in the previous section, the fundamental mechanisms of wrinkle formation are the same. The layered materials *X* and *Y* in Fig. 1 are still instructive: on plant leaves or petals, for example, the microscopic surface wrinkles are undulations restricted to a thin outer cuticular layer bonded to the thicker cell wall of the underlying epidermal cells. Even the more macroscopic layers of animal skin are effectively bonded laminates (outer epidermal, inner dermal, and underlying sub-cutaneous adipose layers), albeit quite structurally complex (Parry 1949).

Translating the general concepts in Fig. 1to biological tissues is most intuitive with soft biological materials such as plant surfaces, animal skin and gut, and fish gills, which are also the most-studied examples of biological wrinkling. In soft biological systems, the strain mismatch between the inner and outer tissues can be caused by various factors and occur over diverse time scales (briefly discussed here). For example, as plant surfaces grow, anisotropic expansion of the epidermal cells coupled with isotropic addition of cuticular components may induce a critical strain mismatch and lead to the formation of nanoscale cuticular ridges (Kourounioti et al. 2013). Alternatively, anisotropic swelling or shrinking of the soft tissue layers (e.g., due to local water loss or gain) may also result in the macroscopic wrinkling of skins (Liu et al. 2013). A familiar example is the skin wrinkling of a shrinking apple. Dehydration of the water-rich inner soft tissue of the fruit decreases its volume and results in the shrinkage of the inner tissue and eventual wrinkling of the thin outer and stiffer cuticle. Similarly, when exposed to water over a long period of time, our fingers wrinkle due to simultaneous swelling of the outer (epidermis) and shrinking of inner (hypodermis) layers of the skin (Sáez and Zöllner 2017). Compressive forces from adjacent tissues, for instance during facial movements or undulatory locomotion of fishes or snakes, may also result in dynamic folding of body surfaces. Similarly, growth of tissues under confinement (e.g., by adjacent tissues/structures) also causes wrinkled patterns in mammalian guts, bacterial biofilms, and plant leaves (Nelson 2016; Huang et al. 2018). On a different time scale, as animal skin ages, permanent changes (loss) in the elasticity of the outer and the inner dermal layers occur, leading to the formation of wrinkles (Krueger and Luebberding 2017; Kruglikov and Scherer 2018).

### Hard biological systems

Although generally less explored, wrinkle-like ornamentations can also be found in some hard tissues, most notably the surfaces of some seed coats (e.g., walnuts), mollusc shells (both bivalves and gastropods), and many vertebrate teeth, spines, and denticles (most studied in fishes and reptiles). Wrinkles in these tissues are not always only a surface phenomenon: in teeth, sculptured surfaces can be the result of folding of both the outer (enamel) and inner (dentin) layers together, or of just one of those layers, meaning wrinkled tissue patterning is sometimes invisible on the tooth's surface (Sander 1999; Kearney and Rieppel 2006; Maxwell et al. 2011; Germain et al. 2016; McCurry et al. 2019).

Formation of wrinkles in these materials is temporally restricted, since the hard nature of the tissues involved make their morphologies comparatively static (i.e., wrinkles can't be formed by simply bunching the material over short time scales) (Checa and Crampton 2002; Kraus and Oka 1967). Since shell and teeth tissues are laid down and then hardened (i.e., their final morphologies aren't a function of remodeling or overgrowth of existing hard tissue), wrinkles have to be deposited in their final configuration during or prior to hardening (sclerotization, mineralization). One strategy for generating periodic hard tissue wrinkles then is through variation in the rate new (non-hardened) material is laid down. This is accomplished for example, by rhythmic expansion and contraction of the bivalve mantle as it deposits new shell material (e.g., Checa and Crampton 2002; Ubukata 2005), or through differential rates of enamel deposition by ameloblasts in teeth (in those surface microstructures formed only by enamel; Sander 1999). Alternatively, wrinkles can be formed by controlling the spatial interactions of the materials involved, as proposed in reaction-diffusion models of gastropod shell growth (summarized in Ubukata 2005) or pre-folding of the dental epithelium prior to production of tooth tissues (in microstructure formed by enamel and dentin; Brink et al. 2015). Although there is a rich history of research into the formation of tooth cusps (e.g., Kraus and Oka 1967) and color patterns in shells (e.g., Meinhardt and Klingler 1987), the root causes of finescale structural ornamentation in hard tissues deserve more attention. With incipient wrinkles appearing prior to tissue hardening, investigation may reveal biophysical parallels with the soft tissue mechanisms we discussed in the previous section.

## Why study biological surface wrinkles?

The growth processes described above, coupled with the available diversity of plant and animal tissue structures and material properties, create a wide palette of wrinkle types in natural systems. As the wrinkles are often on exposed surfaces, the manifold wrinkle morphologies and size scales therefore offer a massive library for understanding natural solutions for surface-environment interactions. And yet, while there has been much success in identifying the structure and function of surface wrinkles in individual biological systems, unified (eco)morphological principles still need to be synthesized and articulated. Characterizing the diversity of wrinkled surface architectures and their function across taxa will provide pathways to evaluate the correlation between material properties and surface morphology and to establish organismal structure–environment links. These pursuits will, in turn, inform diverse disciplines, while concomitantly demanding interdisciplinary perspectives into the material science, developmental biology, and ecology and evolution of natural wrinkled surfaces.

We believe that key to a deeper understanding of the mechanistic bases of wrinkle development and evolution in biological systems is inquiry into the materials and the processing conditions (see Eder et al. 2018) involved in wrinkle formation in a wide range of taxa. This is especially necessary for hard biological systems, where the developmental aspects of their surface wrinkles are still not understood properly. Even slight variations in surface morphology, for example in wrinkle wavelength or height, can have major functional implications. For instance, in the coevolution of insect and plant surfaces, insects developed superior attachment systems to deal with rough plant surfaces during walking (Gorb 2001). However, there exists a critical roughness of 0.3–1 μm at which they still fail to maintain sufficient grip (Peressadko and Gorb 2004; Gorb 2004; Voigt et al. 2008). On wrinkled plant surfaces with characteristic length scales close to this critical roughness, any developmental changes in the cuticle and epidermal cell wall may lead to changes in the roughness and can reduce the effectiveness of the insect attachment (Surapaneni et al. 2020). Similarly, in the case of structural color (i.e., pigment-less color), changes in the wavelength of surface wrinkles on a flower petal during growth may result in variation in the colors diffracted, influencing the organism's ecological interactions (e.g., with pollinators; Moyroud et al. 2017). Moreover, uni-directional strains in the cuticle–cell wall interface lead to the development of parallel wrinkles on flower petals which may act as diffraction gratings for the production of structural color. In contrast, local multidirectional strains result in complex herringbone or labyrinth-like morphologies (Huang et al. 2005), which scatter light in all directions and therefore may not be as effective at color-production (although they could still be useful as insect-repellent surfaces). These ecomorphological studies—by investigating biological wrinkles in their ecological contexts (Campbell and Dean 2019)—augment the exploration of physiological and genetic factors responsible for wrinkle development, and vice versa. At the same time, wrinkled tissues are also attractive models for functional wrinkled biomimetic surface design, because complex surface patterns with well-defined structure can be manufactured with ease at large scales (Rahmawan et al. 2014; Zhang et al. 2020).

## Metadata analysis of the literature

**Fig. 2:**
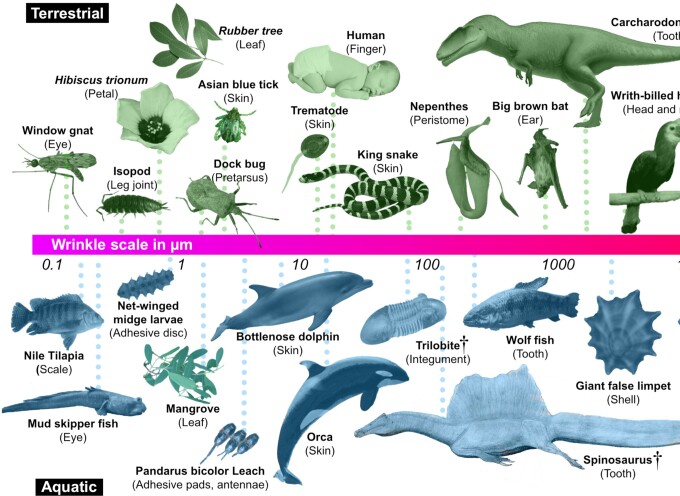
Wrinkles in terrestrial and aquatic organisms: The collage demonstrates (a small subset of) the diversity of organs, organisms (extinct and extant), and size scales where wrinkles occur in natural systems. Extinct species are denoted with a dagger symbol and wrinkle scale indicated by the horizontal bar. *(Please refer to supplementary information for the list of references and image credits.)*

**Fig. 3:**
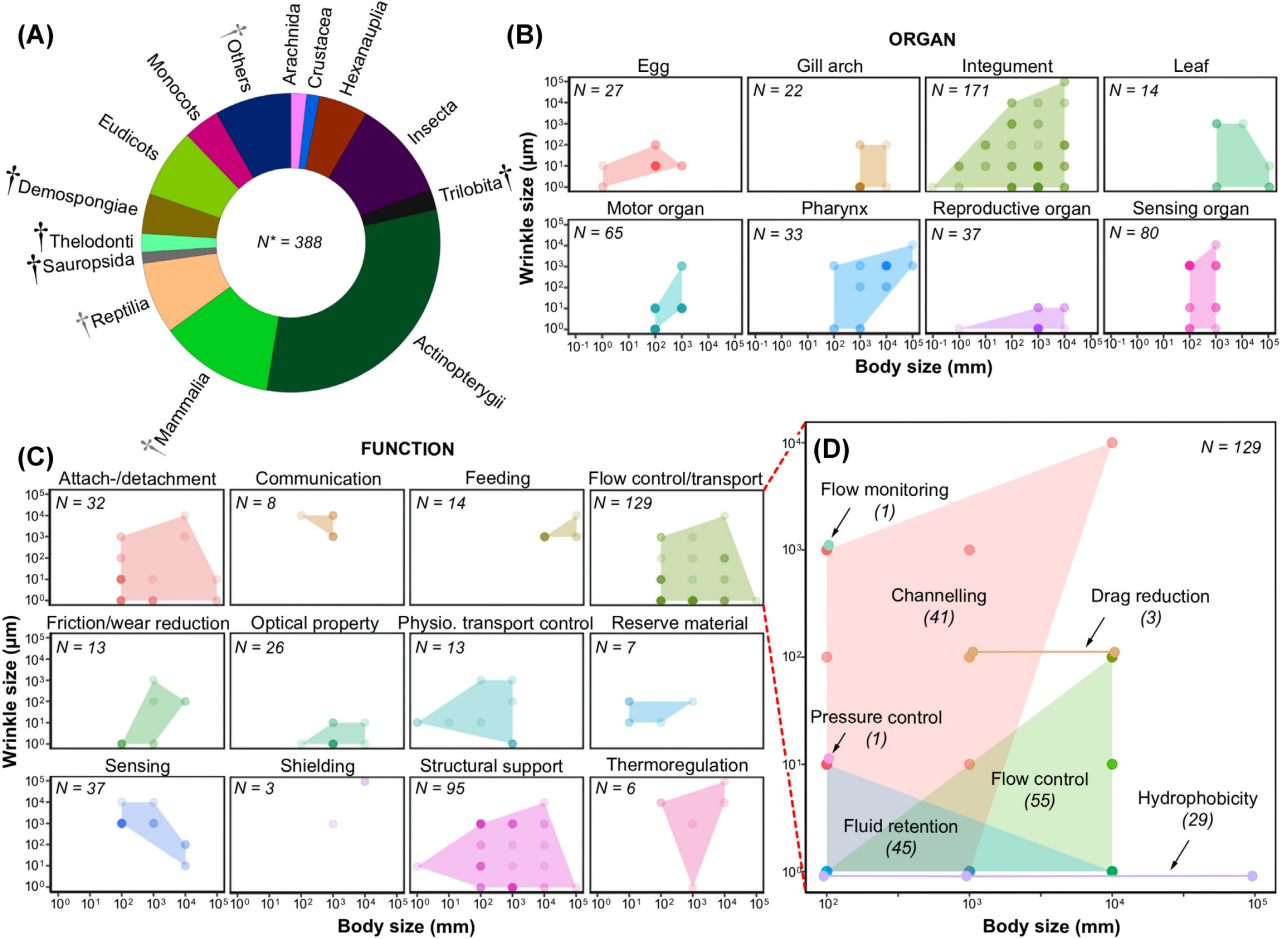
Metadata analysis of wrinkle structure–function relationships. **(A)** Relative frequencies of classes of organisms included in the metadata analysis (only data with frequencies >1% shown). These data correspond to 119 literature sources collected from WOS, from which 388 species (represented as N*) from 158 families were analyzed. The black and gray dagger symbols represent data from classes with all versus some extinct species, respectively. The scatterplots compare orders of magnitude between body size and wrinkle size as a function of **(B)** the organ on which wrinkles occur and **(C)** the functions suggested in the literature sources. The number of observations in each category (*N*) are listed in the corner of each panel. Point transparency in the scatterplots represents instance frequency: darker points indicate high frequency of occurrence, nearly-transparent points are individual species. Function categories often contain interesting variations on a functional theme: For example, **(D)** shows the major function group ‘Flow control/transport’ deconstructed into subfunctions.

Depending on the system and environment, various morphological parameters of wrinkled surfaces are crucial for understanding material–morphology and organism–environment links. Of particular interest are (i) wrinkle size and shape—height, spacing or aspect ratio, sharpness of the wrinkles, (ii) density, (iii) orientation, (iv) complexity or patterns of wrinkles, and (v) type of wrinkling instability (e.g., Fig. 1E; see also Budday et al. 2017). To begin to understand the structure–function aspects of surface wrinkles in different extinct and extant organisms, we conducted a meta-analysis, compiling literature data from BIOSIS Citation Index of the Web of Science (WOS) collection with an advanced search criteria = [(skin OR surface* OR cuticle* OR epidermis*) AND (wrinkles OR ridges OR folds OR striations OR grooves OR undulations)]. The search was filtered by limiting the literature to relevant major concepts in biology (sub-menu of WOS collection) related to organismal biology and ecology (details provided in the supplementary information), which resulted in 18,093 sources. The literature was then refined further by selecting only papers on functional morphology and ecology and relevant citations within those sources. The final analysis involved a total of 119 literature sources corresponding to 388 species from 158 families and 41 classes (both terrestrial and aquatic; extinct and extant, Figs. 2 and 3A). Parameter data on size of organism, wrinkle size (height), wrinkle pattern, environment, and proposed function were collected and analyzed from the literature. In order to reduce complexity arising from analyzing the large data set, we set upper and lower bounds to wrinkle and body size and, to maximize the number of observations in subcategories, we binned functions and organs into major groups (e.g., wrinkles on the esophagus, teeth, and tongue were all coded as “pharynx”). In a preliminary analysis of this large ecofunctional dataset and to begin to understand the scope of the data, we used the *R* software environment (version 4.0.5; R core team, 2021) to generate scatterplots comparing body size and wrinkle size as a function of organ and function (Fig. 3B–D). In the current analyses, we focused only on the maximum order of magnitude (the upper ends of the wrinkle and body size ranges).

The metadata analysis demonstrates that surface wrinkles occur in a wide range of extinct and extant taxa with length scales spanning several orders of magnitude of organism size (from 0.01–10,000 mm) and wrinkle size (0.01–10,000 μm), exhibiting diverse functions that vary with body size, wrinkle size, and environment. In some organs, for example on skin, wrinkles are broadly distributed, occurring in a wide range of wrinkle and body sizes (Fig. 3B: “Integument”), while in others, wrinkles occur over more limited organism sizes (e.g., “Sensing organs”), wrinkle scales (e.g., “Reproductive organs”), or both (e.g., “Gill arches,” “Motor organs”). Similarly, a function may cover large regions of wrinkle versus body size space, meaning that similar functions can be achieved with wrinkles of diverse size scales (Fig. 3C). For instance, wrinkles of any length scale can apparently be involved in providing mechanical support to tissues. For example, sub-micrometer sized ridges on fish epithelial cells provide mechanical support to the epidermis (Sperry and Wassersug 1976), just as millimeter sized radial ridges on hadrosaur skin may serve as structural reinforcement for the integument (Anderson et al. 1998). In contrast, some functions only cover limited regions of the wrinkle–organism space in Fig. 3, and hence may only be characteristic for specific ranges of wrinkle size. For example, there are physical constraints to the sizes at which wrinkles can contribute to hydrophobicity or optical properties, limited by the surface tension of water and the wavelength of light, respectively.

Although the distribution of scatterplot data surely reflects some sampling bias on our part (e.g., toward better-known examples), the general lack of functional data (e.g., many taxonomy papers describe only the presence or absence of wrinkles, not their function), and inevitable bias from the available literature (e.g., the preponderance of studies on wrinkled surfaces in actinopterygian fish), our graphs of wrinkle–organism size space, across natural systems, provide a potential framing of the pathways and perhaps constraints involved in the evolution and adaptation of wrinkled structures. Our results provide a first compendium of surface wrinkle size-versus-function and are valuable in studying the influence of (wrinkle) structure on ecological interactions in a wide range of organisms. We are currently expanding this dataset and refining our organ/function categories to maximize our ability to resolve clear structure–function relationships. Phylogenetic relationships, tissue material properties (e.g., whether hard or soft) and environmental associations will also be considered in this expanded analysis, as these also clearly influence wrinkle functions. For instance, the wrinkles of attachment organs have been proposed to provide adhesion in both terrestrial and aquatic organisms (Ingram and Parker 2006; Clemente et al. 2009), whereas wrinkles of tooth enamel seem to occur predominantly in aquatic feeding animals, in both extinct and extant taxa (McCurry et al. 2019).

## Case studies

### Case study 1—biofilms

**Fig. 4:**
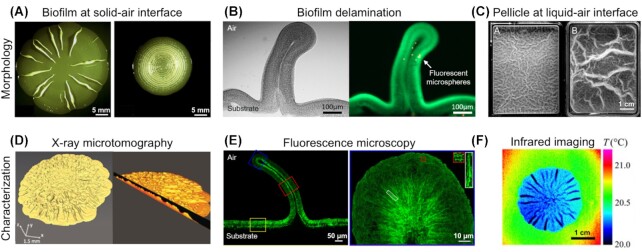
Morphologies of biofilms and pellicles and methods of characterization: **(A)***E. coli* biofilms displaying long radial buckles or dense circumferential wrinkles (modified from Serra et al. 2013 with permission). **(B)** Cross-section of delaminated buckle of *E. coli* biofilm (Ziege, unpublished). **(C)**Pellicles of two wild-type *B. subtilis* strains floating at a liquid-air interface. (modified from Trejo et al. 2013 with permission) **(D)** 3D rendered volume of a μCT scan of an *E. coli* biofilm (modified from Ryzhkov et al. 2021, CC BY-NC-ND 4.0). **(E)** Fluorescence staining of the extracellular polymeric fiber architecture inside *E. coli* biofilm and buckle tip (modified from Serra et al. 2013 with permission). **(F)** Infrared thermograph of a *B. subtilis* biofilm reveals spatial differences in evaporative flux for wrinkled and flat regions (modified from Wilking et al. 2013with permission).

Biofilms form as bacteria produce and assemble a protein- and sugar-based fibrous extracellular matrix in order to protect themselves in challenging environments (Flemming et al. 2016). Bacteria first settle on a surface before proliferating and producing matrix to create a 2D biofilm, which then turns into a more complex 3D structure after 2–3 days, often through wrinkle formation due to mechanical instabilities (Fig. 4A, B) (Serra et al. 2013; Yan et al. 2019). Biofilms forming at liquid/air or liquid/liquid interfaces become floating pellicles that undergo compressive deformation as soon as they cover the whole surface and reach the borders of the container. The biofilm morphology then evolves following the principles of growth under confinement, as shown with *Bacillus subtilis* (Fig. 4C) (Trejo et al. 2013; Douarche et al. 2015) and *Vibrio cholerae* (Qin et al. 2021). Biofilms forming at solid/air interfaces (e.g., on agar-based nutritive substrates) also experience increasing interfacial compressive stresses. Their translation into wrinkling patterns is mediated by both biological (e.g., bacteria growth and matrix synthesis) (Fei et al. 2020) and mechanical determinants (e.g., elastic properties of the biofilm and the substrate, interfacial friction and adhesion energy) (Yan et al. 2019; Ryzhkov et al. 2021). For example, the emergence of periodic or random wrinkling patterns in biofilms was shown to depend on the dominance of radial or circumferential growth rates (Zhang et al. 2016). Interestingly, similar wrinkling behavior was observed for different bacterial strains (*V. cholera*, Yan et al. 2019; *B. subtilis*, Asally et al. 2012; *Escherichia coli*, Ziege et al. 2021), thus pointing toward the dominant role of physical principles in biofilm morphogenesis.

Wrinkles provide the advantage of increasing biofilm surface/volume ratio, which favors access to essential liquid and/or gaseous resources (e.g., nutrients, oxygen) that are exchanged at the interfaces (Jo et al. 2022). Whereas regular diffusion mechanisms would be inadequate to transport such resources over large distances, the spaces created under biofilm wrinkles that have delaminated from the agar surface can promote liquid flow, enabling transport of nutrients throughout the biofilm (Fig. 4B). When these spaces form channels, the flow of nutrient-carrying water into this network is also driven by evaporation (Fig. 4F) (Wilking et al., 2013). The roughness resulting from the emergence of wrinkles also provides the surface of the biofilm with hydrophobic properties that can potentially protect the hosted bacteria from the penetration of antimicrobial solutions, making the region harder to disinfect (Zhang et al. 2017). Finally, the internal stresses responsible for biofilm wrinkling were shown to induce deformation of the substrate, showing that biofilms can effectively restructure their local environment. Disruption was even observed in a more physiological context of biofilm growth on an epithelium monolayer, suggesting a role of wrinkling in some infection mechanisms (Cont et al. 2020).

The development of wrinkling patterns during biofilm formation have been studied at different scales using different tools. For example, stereomicroscopy allows recording the growth of cm-sized biofilms and analysis of the emergence of their intriguing morphologies as a function of time (Ziege et al. 2021). Particle tracking has also been used to refine our understanding of the motion of matter and assess the “tectonic” behavior of biofilms (Asally et al. 2012). To obtain 3D structural data at a finer length scale, high-resolution confocal microscopy can be performed after staining, embedding, and slicing the biofilms, as performed on *E. coli* biofilms grown at solid/air interface (Fig. 4E) (Serra et al. 2013). While standard scanning electron microscopy can appear useful, the requisite delicate sample preparation makes it difficult to implement. When a biofilm's material density is high enough compared to its substrate, X-ray micro-computer tomography can also be used to reconstruct the three-dimensional surface geometry, opening possibilities for surface/volume ratio computation (Fig. 4D) (Ryzhkov et al. 2021). Ultimately, combining these strategies may help to clarify if wrinkles are “only” a consequence of the biofilm growth process or if bacteria generate the biofilm geometry “on purpose” for the additional advantages it provides compared to flat films.

### Case study 2—plant surfaces

Being sessile, a dominant way plants adapt to their surroundings is through their highly functional surfaces. The plant cuticle, the thin outermost layer that wraps most land plants, is multifunctional and critical for plant environmental interactions. It is rich in lipid polyesters cutin and cutan; contains intracuticular waxes, polysaccharides, and phenolics (Jeffree 2006; Domínguez et al. 2011); and is supported by a thick cell wall, polysaccharide-rich and mainly consisting of cellulose microfibrils, hemicellulose, pectin, glycoproteins, and lignin (Cosgrove 2005).

Cuticular wrinkles (or ridges) are common on various plant organs such as leaves, flower petals, sepals, and stems, with length scales ranging from 100 nm to 1 μm, and with morphologies varying depending on location and species (Barthlott and Ehler 1977; Koch et al. 2008), and may serve a variety of functions (Figs. 2 and 3). These differences in microscopic surface structure could possibly be related to ecological function—parallel ridges on the flower petals may serve as color-producing structures to attract pollinators and complex ridges on the leaf surfaces can deter herbivores. In fact, the ridges on the petal surfaces may also reduce grip of insect pollinators during interaction, but the conical morphology of epidermal cell structures on most petals enables sufficient locking of the insect claws in between the cells (Whitney et al. 2011).

**Fig. 5:**
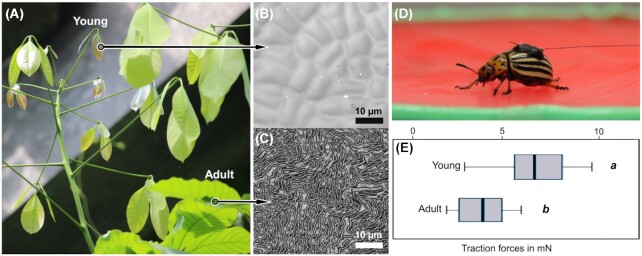
Ontogenetic variations in the cuticular structure and insect attachment: **(A)** Young and adult leaves of *H. brasiliensis*, **(B)** smooth cuticle on young, and **(C)** labyrinth-like wrinkles on adult leaf surfaces (imaged using a laser-scanning confocal microscope). Insect walking experiments on the polymer replicas of leaves **(D)** show that the traction forces are significantly higher on young leaf surfaces than on adult leaf surfaces **(E)** (adapted from Surapaneni et al. 2020).

During the ontogeny of plant organs, continuous and sometimes rapid changes in the composition and the structural properties occur in the cuticle and cell wall layers due to the deposition of new material and growth (Jeffree 2006). Strain mismatches may occur in different directions at the cuticle–cell wall interface during these structural alterations and lead to the wrinkling of the cuticle. Recent studies (sepals, *Arabidopsis thaliana*, Hong et al. 2017; petals, *daisy, Kalanchoe blossfeldiana, and Eustoma grandiflorum*, Huang et al. 2017; leaves, *Hevea brasiliensis* (Fig. 5A–C), Surapaneni et al. 2020; leaves, *Schismatoglottis calyptrata*, Surapaneni et al. 2021) show that the wrinkles form spontaneously at intermediate stages during the ontogeny and develop progressively, either in an acropetal (base to apex) or a basipetal (apex to base) fashion, suggesting the influence of cell developmental processes on wrinkle formation (Hong et al. 2017).

Besides chemistry, such ontogenetic changes in the structure of the cuticle itself can alter functional and defensive properties of the plant surfaces with age. For instance, insects have a better grip on smooth young and overmatured *H. brasiliensis* leaf surfaces when compared with the adult leaf surfaces with cuticular ridges (Fig. 5D, E), and therefore may interact differently with young and adult leaves. The results also reflect the changes in the structural defenses of plant leaves during ontogeny (Surapaneni et al. 2020). Besides insect detachment and structural color, the cuticular ridges may control wetting of the plant surface (Prüm et al. 2012), act as tactile surfaces for insects (Koch et al. 2008) and help in avoiding cuticle cracking, thus maintaining the structural integrity of the growing plant organs (Casado and Heredia 2001; Domínguez et al. 2011).

The variation in the micromorphology of the wrinkles within and across species, and during ontogeny can have significant impact on the way plants interact with their environment. While the available literature provides insights on structure–function relationships of wrinkled plant cuticles, the influence of variations in the material composition and mechanics across species and during ontogeny on wrinkle micromorphology still needs to be resolved. This knowledge complemented with genetic studies on wrinkle formation (Shi et al. 2011; Hong et al. 2017) may allow development of crops and artificial surfaces (Bergmann et al. 2020) for household and industry, where insect deterrence and/or resistance are imparted by structure rather than chemicals.

### Case study 3—basking shark gill raker surfaces

**Fig. 6:**
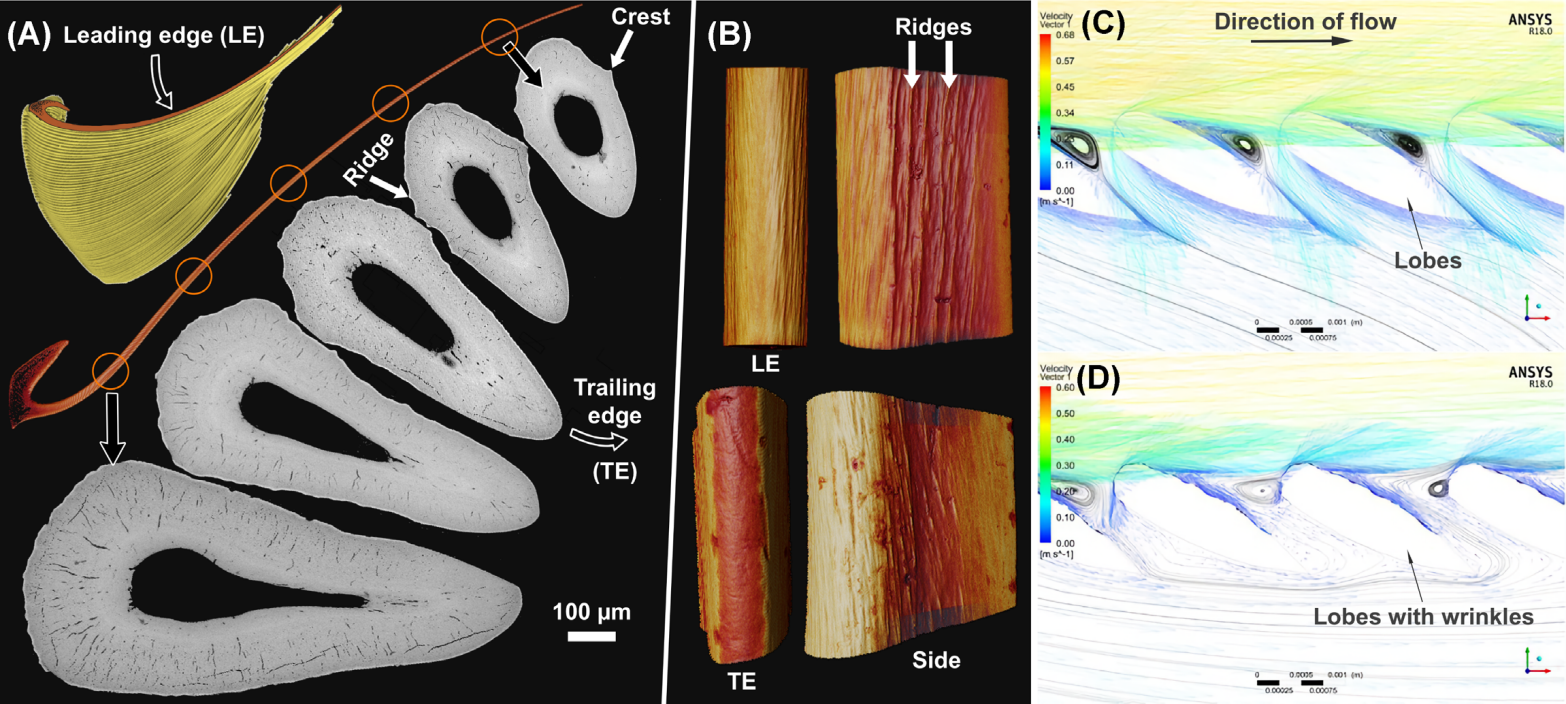
Finescale wrinkling of the surfaces of the gill rakers of the basking shark (*C. maximus*). **(A)** Rakers (orange) are stiff thread-like elements with hooked bases that nest together in large filter arrays (yellow). Raker threads have hydrofoil cross-sections (grayscale images), with a rounded leading edge (LE), and tapered trailing edge (TE). **(B)** Raker segments showing the prominent lengthwise wrinkles visible on the trailing edge and sides, but not the leading edge. CFD models of **(C)** smooth and **(D)** wrinkled raker-like cross sections suggest these surface structures could alter bulk flow patterns to affect filter performance. *(All images in A and B from μCT, except grayscale SEM images in A.)*

The largest fish in the ocean feed on some of the smallest prey. Basking sharks (*Cetorhinus maximus*) are the second largest fish and, like their relatives whale sharks and devil rays, are planktivorous suspension feeders. Filter feeding evolved multiple times independently in sharks and rays (Paig-Tran and Summers 2014), resulting in disparate filtering anatomies and behaviors: the filter of basking sharks is strikingly different from the porous pad (Motta et al. 2010) or lobular (Paig-Tran et al. 2013) filters of whale sharks and mobulids, respectively, instead involving thread-like gill rakers (Fig. 6), arrayed by the thousands along the gill arches, jutting into the pharyngeal passages leading to the gill slits. When feeding, basking sharks greatly expand and splay their gill arches, plowing through the water at mean swimming speeds of 0.85 m s^–1^, making them the only obligate ram filter-feeding sharks (Sims 1999).

Because of the exceptional experimental challenges of *in-vivo* investigation of suspension filtering in large pelagic animals, the physical principles governing how basking shark filters interact with flow and extract plankton are unknown. Although it was assumed that gill rakers are blunt sieves for plankton retention, as also previously thought for whale baleen (Werth and Potvin 2016), recent hypotheses argue for less direct filtration mechanisms. Raker morphology and orientation, for example, could encourage flow to run along raker surfaces rather than perpendicular to them (cross-flow filtration, Sanderson et al. 2001; Sanderson et al. 2016) or to create vortices that either cause plankton to accumulate at the base of rakers for later collection (vortical cross-step filtration, Sanderson et al. 2016) or to skip off the filter and continue on to the esophagus (ricochet filtration, Divi et al. 2018).

In examining gill raker arrayment (by μCT and SEM; Fig. 6A and B) to clarify raker orientation in flow and determine which filtering mechanism is in play, we discovered a distinct fine-scale surface wrinkling that may also coordinate fluid movement. In cross-section, raker threads are hollow hydrofoils (Fig. 6A), with rounded leading edges facing into incoming flow. Especially at their mid-shafts, rakers’ elliptical cross-sections are not entirely symmetrical, bearing conspicuous crests (∼30–50 μm wide) protruding off either side, near the thickest points of the hydrofoil. Smaller scale surface wrinkles (<15 μm wide) also corrugate the cross-sections’ margins, particularly pronounced on the mid-raker's leading edge, decreasing in prominence toward the distal tip of the thread. The wrinkles form cord-like surface ridgelines, hundreds of microns long, and oriented roughly parallel to the raker's long axis. Raker trailing edges, on the other hand, are smooth (Fig. 6B).

Given that we found wrinkles only on the leading edge of rakers, we hypothesized they could act to entrain spanwise flow, as proposed for ridged skin denticles, located where water exits the gill slits in sharks (Gabler-Smith et al. 2021). To test the effect of adding wrinkles to filter surfaces (and since flow characteristics of basking shark filters are not yet available for ground-truthing), we performed a virtual 3D simulation (ANSYS ICEM, 2018a) mimicking a model for ricochet filtration in manta ray gill lobes (Divi et al. 2018). Transient computational fluid dynamics simulations were performed (ANSYS CFX, 2018b) for lobes with smooth surfaces (Fig. 6C) and wrinkled ones (Fig. 6D). Smooth lobe simulations agreed with Divi et al's models, with vortices producing low-pressure zones downstream of lobes, helping to effectively occlude gaps between lobes and increase crossflow filtration efficiency. As wrinkles were introduced to models, however, bulk flow patterns changed considerably, with low-pressure vortices shifting upstream of lobes and even flow reversals appearing in some locations (Fig. 6D).

Although the cross-sectional aspect ratios of manta and basking shark filters differ considerably (e.g., basking shark rakers are ∼⅓ that of manta lobes), our simulations suggest even fine-scale wrinkles have potential for significant local flow control. Our future CFD analyses will incorporate measurements from physical experiments to determine the extent to which raker surface wrinkling—even at a microstructural scale—may impact particle retention efficiency for the basking shark's massive biological filter. These results combined with anatomical and material data from rakers and raker arrays therefore, also represent valuable natural prototypes for inspiring large-scale dynamic industrial filters or lightweight yet robust filaments with interlocking and/or hydrodynamic cross sections (e.g., for textile vortex spinning).

## Summary

Using broad scale meta-analysis of literature and case studies, we demonstrate the huge diversity and structure–function aspects of biological surface wrinkles in a wide range of extinct and extant organisms (Figs. 2 and 3). From the meta-analysis, we demonstrate the utility of a deep-dive to parse out general performance principles in natural wrinkled surfaces, establishing that the functional morphology of surface wrinkles may be influenced by or linked to the size of the organism, wrinkle size and the organism's environment. These results offer a large library of functions in relation to the structure of surface wrinkles in a wide range of terrestrial and aquatic organisms and therefore help in understanding the ecomorphology of surface wrinkles (and eventually perhaps the selective pressures that shape them). Knowing how these structures are employed across taxa could also be useful in examining their efficiency in ecological interactions, compared with their evolutionary alternatives (e.g., in closely related structures). These pursuits will help in establishing cross-disciplinary links (e.g., developmental biology with mechanical ecology) and in distilling core biomechanical principles to aid in the development of highly functionalized and adaptive biomimetic surfaces. Our reported case studies, for example, highlight natural examples that could inform real-world textile, agriculture and industrial filter applications. The building constraints on biological systems—for example, their limited chemistry and low-temperature synthesis conditions (Eder et al. 2018)—regulate the formation, development, and function of surface wrinkles, but also make nature a great source of inspiration for technical applications.

Beyond characterizing the morphological and functional diversity of natural wrinkles, we believe, a grand challenge in the study of wrinkled biological surfaces (and many other structured biological materials) is determining the degree to which “structures” in different systems are adaptations that were selected for versus being “merely” byproducts of growth and/or physics (albeit functional ones). Key to disentangling this and the factors impelling wrinkle formation is building time into comparative analyses, exploring the ontogenetic development of wrinkles (e.g., contrasting hard and soft tissue development), as well as the evolution of wrinkled systems in the context of organismal ecology. In this regard, we believe our metadata analysis can function much like a material selection chart (Ashby 2017), providing a series of structural and functional variables to define valuable ingroups for investigation. For example, our meta-analysis demonstrated that wrinkle patterning is particularly structurally and phylogenetically diverse in biology and may prove a fruitful variable to explore, manifesting as radial (e.g., on seashells) or parallel patterns (e.g., on dolphin skin), but also complex maze-like topographies (e.g., on fish skin or plant leaves). Within this diversity, parallel enamel ridges seemed to have convergently evolved specifically on tooth surfaces in a wide range of extinct and extant aquatic feeding (terrestrial and aquatic) animals (see also McCurry et al. 2019), pointing perhaps to potentially useful models for investigating functional advantages for or common factors driving tooth surface sculpturing.

Given the need to resolve, at multiple scales, wrinkle form–function relationships and the factors shaping them, we hope our work will act to spur more fluid multidisciplinary interactions, such as between biologists (e.g., with perspectives in organismal ecology and environmental interactions) and material scientists (e.g., versed in the physics of surface interactions and tribology) (Campbell and Dean 2019). The case studies we presented are illustrations of the value of such interactions; our preliminary observations on biofilms and basking shark filters, for example, provided the baseline for current, deeper investigations into the interplay of material “ecology” and tissue morphology (and morphogenesis) in both systems. Similarly, our ongoing extended literature meta-analysis will, by incorporating phylogenetic relationships, tissue properties, and ecological interactions, not only complement our understanding of functional morphological diversity, but also help build maps for tracing evolutionary pathways of functional convergence of biological surface wrinkles.

## Supplementary Material

icac079_Supplemental_File

## Data Availability

The datasets supporting the results presented in this article are included in the supplementary information. The R-script used to analyse the data is available upon request.
