# Supplementary material for: Groovy and Gnarly: Surface Wrinkles as a Multifunctional Motif for Terrestrial and Marine Environments
Source: Integr Comp Biol. 2022 Jun 8;62(3):749–61. doi: 10.1093/icb/icac079 (PMC9703940; doi:10.1093/icb/icac079)
Supplement: icac079_Supplemental_File [file icac079_supplemental_file.zip › icb-2022-0098-File008.docx]

Supplementary information for

**Groovy and gnarly: Surface wrinkles as a multifunctional motif for terrestrial and marine environments**

Literature collection for meta-data analysis from Web of Science (WOS) database

The literature search procedure in Web of Science database for the meta-data analysis is shown as a flow chart:

**BIOSIS Citation Index database selection**

**Advanced search criteria**

((skin OR surface* OR cuticle* OR epidermis*) AND (wrinkles OR ridges OR folds OR striations OR grooves OR undulations)) – resulted in 80127 literature sources

**Filtering of major concepts**

(*Major concepts selected:* Development, Morphology, Biomaterials, Botany, Zoology, Ecology Environmental Sciences, Evolution and Adaptation, Freshwater ecology, Terrestrial ecology, Behaviour, Ecology, Paleobiology, Marine ecology, Physics, Estuarine ecology, Groundwater ecology, Subterranean ecology) *–* resulted in 18093 literature sources

Further refinement of literature on functional morphology and ecology and relevant citations within those sources

**Final analysis**

on 119 literature sources corresponding to 388 species from 158 families and 41 classes (both terrestrial and aquatic; extinct and extant – also see Fig. 3 in the main text
