# Supplementary material for: Groovy and Gnarly: Surface Wrinkles as a Multifunctional Motif for Terrestrial and Marine Environments
Source: Integr Comp Biol. 2022 Jun 8;62(3):749–61. doi: 10.1093/icb/icac079 (PMC9703940; doi:10.1093/icb/icac079)
Supplement: icac079_Supplemental_File [file icac079_supplemental_file.zip › icb-2022-0098-File009.pdf]

	A	B	C	D	E	F
1	**Supplementary information - Meta-analysis data**				Red text = approximation (not derived from reference)	
2	**Environment**	**Kingdom**	**Phylum**	**Class**	**Order**	**Family**
3	Aquatic	Animalia	Chordata	Reptilia	Squamata	Acrochordidae
4	Aquatic	Animalia	Chordata	Reptilia	Squamata	Acrochordidae
5	Aquatic	Animalia	Chordata	Actinopterygii	Perciformes	Cichlidae
6	Aquatic	Animalia	Chordata	Actinopterygii	Perciformes	Cichlidae
7	Aquatic	Animalia	Chordata	Actinopterygii	Perciformes	Cichlidae
8	Aquatic	Animalia	Chordata	Actinopterygii	Ovalentaria incertae sedis	Pomacentridae
9	Aquatic	Animalia	Chordata	Actinopterygii	Ovalentaria incertae sedis	Pomacentridae
10	Aquatic	Animalia	Chordata	Actinopterygii	Scorpaeniformes	Anarhichadidae
11	Aquatic	Animalia	Chordata	Actinopterygii	Perciformes	Apogonidae
12	Aquatic	Animalia	Chordata	Actinopterygii	Perciformes	Apogonidae
13	Aquatic	Animalia	Chordata	Actinopterygii	Perciformes	Apogonidae
14	Aquatic	Animalia	Chordata	Actinopterygii	Perciformes	Apogonidae
15	Aquatic	Animalia	Chordata	Actinopterygii	Perciformes	Apogonidae
16	Aquatic	Animalia	Chordata	Actinopterygii	Perciformes	Apogonidae
17	Aquatic	Animalia	Chordata	Actinopterygii	Perciformes	Apogonidae
18	Aquatic	Animalia	Chordata	Actinopterygii	Perciformes	Apogonidae
19	Aquatic	Animalia	Chordata	Actinopterygii	Kurtiformes	Apogonidae
20	Aquatic	Animalia	Chordata	Actinopterygii	Perciformes	Apogonidae
21	Aquatic	Animalia	Chordata	Actinopterygii	Perciformes	Apogonidae
22	Aquatic	Animalia	Chordata	Actinopterygii	Perciformes	Apogonidae
23	Aquatic	Animalia	Chordata	Actinopterygii	Perciformes	Apogonidae
24	Aquatic	Animalia	Chordata	Actinopterygii	Perciformes	Apogonidae
25	Aquatic	Animalia	Chordata	Actinopterygii	Perciformes	Apogonidae
26	Aquatic	Animalia	Chordata	Actinopterygii	Perciformes	Apogonidae
27	Aquatic	Animalia	Chordata	Actinopterygii	Perciformes	Apogonidae
28	Aquatic	Animalia	Chordata	Actinopterygii	Perciformes	Apogonidae
29	Aquatic	Animalia	Chordata	Actinopterygii	Perciformes	Apogonidae
30	Aquatic	Animalia	Chordata	Actinopterygii	Perciformes	Apogonidae
31	Aquatic	Animalia	Chordata	Actinopterygii	Perciformes	Apogonidae

	G	H	I	J	K	L	M
1							
2	**Species**	**Organ**	**Ontogeny**	**Wrinkle orientation**	**Organism_si**	**Wrinkle_size_µm**	**Function**
3	*Acrochordus javanicus*	Tooth	Adult	Parallel	1000-10000	10-100	Drag reduction
4	*Acrochordus javanicus*	Tooth	Adult	Parallel	1000-10000	10-100	Structural support
5	*Aequidens portalegrensis*	Scale		Maze	100-1000	0.1-1	Flow control
6	*Aequidens portalegrensis*	Scale		Maze	100-1000	0.1-1	Fluid retention
7	*Aequidens portalegrensis*	Scale		Maze	100-1000	0.1-1	Structural support
8	*Ampohiprion percula (Lacép*	Scale	Adult	Maze	100-1000		Chanelling
9	*Ampohiprion percula (Lacép*	Scale	Adult	Maze	100-1000		Friction and Wear reduction
10	*Anarhichas lupus*	Tooth		Infoldings	100-1000	100-1000	Structural support
11	*Apogon cookii*	Tongue	Adult	Maze	10-100	0.1-1	
12	*Apogon cyanosoma*	Tongue	Adult	Parallel	10-100	100-1000	
13	*Apogon evermanni*	Egg	Adult	Maze	100-1000		Adhesion and friction
14	*Apogon evermanni*	Egg	Adult	Maze	100-1000		Chanelling
15	*Apogon evermanni*	Egg	Adult	Maze	100-1000		Structural support
16	*Apogon fraenatus*	Egg	Adult	Maze	10-100		Adhesion and friction
17	*Apogon fraenatus*	Egg	Adult	Maze	10-100		Chanelling
18	*Apogon fraenatus*	Egg	Adult	Maze	10-100		Structural support
19	*Apogon frenatus*	Tongue	Adult	Maze	10-100	0.1-1	
20	*Apogon hungi*	Egg	Adult	Maze	100-1000		Adhesion and friction
21	*Apogon hungi*	Egg	Adult	Maze	100-1000		Chanelling
22	*Apogon hungi*	Egg	Adult	Maze	100-1000		Structural support
23	*Apogon semiornatus*	Egg	Adult	Maze	10-100		Adhesion and friction
24	*Apogon semiornatus*	Egg	Adult	Maze	10-100		Chanelling
25	*Apogon semiornatus*	Egg	Adult	Maze	10-100		Structural support
26	*Apogon talboti*	Egg	Adult	Maze	10-100	1-10	Adhesion and friction
27	*Apogon talboti*	Egg	Adult	Maze	10-100	1-10	Chanelling
28	*Apogon talboti*	Egg	Adult	Maze	10-100	1-10	Structural support
29	*Archamia bilineata*	Egg	Adult	Maze	10-100		Adhesion and friction
30	*Archamia bilineata*	Egg	Adult	Maze	10-100		Chanelling
31	*Archamia bilineata*	Egg	Adult	Maze	10-100		Structural support

	N
1	
2	**Literature**
3	Vaeth RH, Rossman DA, Shoop W. 1985 Observations of Tooth Surface Morphology in Snakes. Journal of Herpetology 19, 20. (doi:10.2307/1564416)
4	Vaeth RH, Rossman DA, Shoop W. 1985 Observations of Tooth Surface Morphology in Snakes. Journal of Herpetology 19, 20. (doi:10.2307/1564416)
5	Fishelson L. 1984 A comparative study of ridge-mazes on surface epithelial cell-membranes of fish scales (Pisces, Teleostei). Zoomorphology 104, 231–238. (
6	Fishelson L. 1984 A comparative study of ridge-mazes on surface epithelial cell-membranes of fish scales (Pisces, Teleostei). Zoomorphology 104, 231–238. (
7	Fishelson L. 1984 A comparative study of ridge-mazes on surface epithelial cell-membranes of fish scales (Pisces, Teleostei). Zoomorphology 104, 231–238. (
8	Hunter CR, Nayudu PL. 1978 Surface folds in superficial epidermal cells of three species of teleost fish. J Fish Biology 12, 163–166. (doi:10.1111/j.1095-864
9	Hunter CR, Nayudu PL. 1978 Surface folds in superficial epidermal cells of three species of teleost fish. J Fish Biology 12, 163–166. (doi:10.1111/j.1095-864
10	Thangadurai S, Brumfeld V, Milgram J, Li L, Shahar R. 2022 Osteodentin in the Atlantic wolffish (Anarhichas lupus): Dentin or bone? Journal of Morphology
11	Fishelson L, Delarea Y, Zverdling A. 2004 Taste bud form and distribution on lips and in the oropharyngeal cavity of cardinal fish species (Apogonidae, Teleo
12	Fishelson L, Delarea Y, Zverdling A. 2004 Taste bud form and distribution on lips and in the oropharyngeal cavity of cardinal fish species (Apogonidae, Teleo
13	Fishelson L, Gon O. 2008 Comparative oogenesis in cardinal fishes (Apogonidae, Perciformes), with special focus on the adaptive structures of the egg enve
14	Fishelson L, Gon O. 2008 Comparative oogenesis in cardinal fishes (Apogonidae, Perciformes), with special focus on the adaptive structures of the egg enve
15	Fishelson L, Gon O. 2008 Comparative oogenesis in cardinal fishes (Apogonidae, Perciformes), with special focus on the adaptive structures of the egg enve
16	Fishelson L, Gon O. 2008 Comparative oogenesis in cardinal fishes (Apogonidae, Perciformes), with special focus on the adaptive structures of the egg enve
17	Fishelson L, Gon O. 2008 Comparative oogenesis in cardinal fishes (Apogonidae, Perciformes), with special focus on the adaptive structures of the egg enve
18	Fishelson L, Gon O. 2008 Comparative oogenesis in cardinal fishes (Apogonidae, Perciformes), with special focus on the adaptive structures of the egg enve
19	Fishelson L, Delarea Y, Zverdling A. 2004 Taste bud form and distribution on lips and in the oropharyngeal cavity of cardinal fish species (Apogonidae, Teleo
20	Fishelson L, Gon O. 2008 Comparative oogenesis in cardinal fishes (Apogonidae, Perciformes), with special focus on the adaptive structures of the egg enve
21	Fishelson L, Gon O. 2008 Comparative oogenesis in cardinal fishes (Apogonidae, Perciformes), with special focus on the adaptive structures of the egg enve
22	Fishelson L, Gon O. 2008 Comparative oogenesis in cardinal fishes (Apogonidae, Perciformes), with special focus on the adaptive structures of the egg enve
23	Fishelson L, Gon O. 2008 Comparative oogenesis in cardinal fishes (Apogonidae, Perciformes), with special focus on the adaptive structures of the egg enve
24	Fishelson L, Gon O. 2008 Comparative oogenesis in cardinal fishes (Apogonidae, Perciformes), with special focus on the adaptive structures of the egg enve
25	Fishelson L, Gon O. 2008 Comparative oogenesis in cardinal fishes (Apogonidae, Perciformes), with special focus on the adaptive structures of the egg enve
26	Fishelson L, Gon O. 2008 Comparative oogenesis in cardinal fishes (Apogonidae, Perciformes), with special focus on the adaptive structures of the egg enve
27	Fishelson L, Gon O. 2008 Comparative oogenesis in cardinal fishes (Apogonidae, Perciformes), with special focus on the adaptive structures of the egg enve
28	Fishelson L, Gon O. 2008 Comparative oogenesis in cardinal fishes (Apogonidae, Perciformes), with special focus on the adaptive structures of the egg enve
29	Fishelson L, Gon O. 2008 Comparative oogenesis in cardinal fishes (Apogonidae, Perciformes), with special focus on the adaptive structures of the egg enve
30	Fishelson L, Gon O. 2008 Comparative oogenesis in cardinal fishes (Apogonidae, Perciformes), with special focus on the adaptive structures of the egg enve
31	Fishelson L, Gon O. 2008 Comparative oogenesis in cardinal fishes (Apogonidae, Perciformes), with special focus on the adaptive structures of the egg enve

	A	B	C	D	E	F
32	Aquatic	Animalia	Chordata	Actinopterygii	Perciformes	Apogonidae
33	Aquatic	Animalia	Chordata	Actinopterygii	Perciformes	Apogonidae
34	Aquatic	Animalia	Chordata	Actinopterygii	Perciformes	Apogonidae
35	Aquatic	Animalia	Chordata	Actinopterygii	Perciformes	Apogonidae
36	Aquatic	Animalia	Chordata	Actinopterygii	Perciformes	Apogonidae
37	Aquatic	Animalia	Chordata	Actinopterygii	Perciformes	Apogonidae
38	Aquatic	Animalia	Arthropoda	Arachnida	Araneae	Dictynidae
39	Aquatic	Animalia	Chordata	Actinopterygii	Perciformes	Artedidraconidae
40	Aquatic	Animalia	Chordata	Actinopterygii	Perciformes	Artedidraconidae
41	Aquatic	Animalia	Chordata	Actinopterygii	Perciformes	Cichlidae
42	Aquatic	Animalia	Chordata	Actinopterygii	Perciformes	Cichlidae
43	Aquatic	Animalia	Chordata	Actinopterygii	Perciformes	Cichlidae
44	Aquatic	Animalia	Chordata	Actinopterygii	Perciformes	Cichlidae
45	Aquatic	Animalia	Chordata	Actinopterygii	Perciformes	Cichlidae
46	Aquatic	Animalia	Chordata	Actinopterygii	Perciformes	Cichlidae
47	Aquatic	Animalia	Chordata	Chondrichthyes	Rajiformes	Elasmobranchii
48	Aquatic	Animalia	Arthropoda	Insecta	Diptera	Blephariceridae
49	Aquatic	Animalia	Arthropoda	Insecta	Diptera	Blephariceridae
50	Aquatic	Animalia	Arthropoda	Insecta	Diptera	Blephariceridae
51	Aquatic	Animalia	Chordata	Actinopterygii	Perciformes	Gobiidae
52	Aquatic	Animalia	Chordata	Actinopterygii	Perciformes	Gobiidae
53	Aquatic	Animalia	Chordata	Actinopterygii	Perciformes	Gobiidae
54	Aquatic	Animalia	Chordata	Actinopterygii	Perciformes	Gobiidae
55	Aquatic	Animalia	Rotifera	Monogononta	Ploima	Brachionidae
56	Aquatic	Animalia	Rotifera	Monogononta	Ploima	Brachionidae
57	Aquatic	Animalia	Chordata	Actinopterygii	Cypriniformes	Cyprinidae
58	Aquatic	Animalia	Chordata	Actinopterygii	Cypriniformes	Cyprinidae
59	Aquatic	Animalia	Chordata	Actinopterygii	Perciformes	Serranidae
60	Aquatic	Animalia	Chordata	Actinopterygii	Perciformes	Serranidae
61	Aquatic	Animalia	Chordata	Actinopterygii	Perciformes	Serranidae
62	Aquatic	Animalia	Chordata	Actinopterygii	Perciformes	Serranidae
63	Aquatic	Animalia	Chordata	Actinopterygii	Perciformes	Serranidae
64	Aquatic	Animalia	Chordata	Actinopterygii	Perciformes	Serranidae

	G	H	I	J	K	L	M
32	*Archamia lineolata*	Egg	Adult	Maze	10-100	1-10	Adhesion and friction
33	*Archamia lineolata*	Egg	Adult	Maze	10-100	1-10	Chanelling
34	*Archamia lineolata*	Egg	Adult	Maze	10-100	1-10	Structural support
35	*Archamia mozambiquensis*	Egg	Adult	Maze	10-100		Adhesion and friction
36	*Archamia mozambiquensis*	Egg	Adult	Maze	10-100		Chanelling
37	*Archamia mozambiquensis*	Egg	Adult	Maze	10-100		Structural support
38	*Argyroneta aquatica*	Skin	Adult	Labyrinth	10-100	1-10	Structural support
39	*Artedidraco glareobarbatus*	Barbel	Adult	Labyrinth	100-1000	1000-10000	Lure
40	*Artedidraco glareobarbatus*	Barbel	Adult	Labyrinth	100-1000	1000-10000	Sensing
41	*Astatotilapia flaviijosephi*	Scale		Maze	10-100	0.1-1	Flow control
42	*Astatotilapia flaviijosephi*	Scale		Maze	10-100	0.1-1	Flow control
43	*Astatotilapia flaviijosephi*	Scale		Maze	10-100	0.1-1	Fluid retention
44	*Astatotilapia flaviijosephi*	Scale		Maze	10-100	0.1-1	Fluid retention
45	*Astatotilapia flaviijosephi*	Scale		Maze	10-100	0.1-1	Structural support
46	*Astatotilapia flaviijosephi*	Scale		Maze	10-100	0.1-1	Structural support
47	*Atlanticopristis equatorialis*	Tooth		Parallel	10-100	100-1000	
48	*Blepharicera sp.*	Leg	Larvae	Radial	10-100	1-10	Adhesion and friction
49	*Blepharicera sp.*	Leg	Larvae	Radial	10-100	1-10	Chanelling
50	*Blepharicera sp.*	Leg	Larvae	Radial	10-100	1-10	Pressure control
51	*Boleophthalmus pectinirostri*	Eye	Adult	Maze	100-1000	0.1-1	Fluid retention
52	*Boleophthalmus pectinirostri*	Eye	Larvae	Labyrinth	10-100	0.1-1	Fluid retention
53	*Boleophthalmus pectinirostri*	Eye	Adult	Maze	100-1000	0.1-1	Structural support
54	*Boleophthalmus pectinirostri*	Eye	Larvae	Labyrinth	10-100	0.1-1	Structural support
55	*Brachionus plicatilis*	Egg	Egg	Labyrinth	0.1-1	0.1-1	
56	*Brachionus rotundiformis*	Egg	Egg	Labyrinth	0.1-1	0.1-1	
57	*Carassius auratus auratus (L*	Scale	Adult	Maze	100-1000		Chanelling
58	*Carassius auratus auratus (L*	Scale	Adult	Maze	100-1000		Friction and Wear reduction
59	*Cephalopholis argus*	Scale		Maze	100-1000	0.1-1	Flow control
60	*Cephalopholis argus*	Scale		Maze	100-1000	0.1-1	Flow control
61	*Cephalopholis argus*	Scale		Maze	100-1000	0.1-1	Fluid retention
62	*Cephalopholis argus*	Scale		Maze	100-1000	0.1-1	Fluid retention
63	*Cephalopholis argus*	Scale		Maze	100-1000	0.1-1	Structural support
64	*Cephalopholis argus*	Scale		Maze	100-1000	0.1-1	Structural support

	N
32	Fishelson L, Gon O. 2008 Comparative oogenesis in cardinal fishes (Apogonidae, Perciformes), with special focus on the adaptive structures of the egg enve
33	Fishelson L, Gon O. 2008 Comparative oogenesis in cardinal fishes (Apogonidae, Perciformes), with special focus on the adaptive structures of the egg enve
34	Fishelson L, Gon O. 2008 Comparative oogenesis in cardinal fishes (Apogonidae, Perciformes), with special focus on the adaptive structures of the egg enve
35	Fishelson L, Gon O. 2008 Comparative oogenesis in cardinal fishes (Apogonidae, Perciformes), with special focus on the adaptive structures of the egg enve
36	Fishelson L, Gon O. 2008 Comparative oogenesis in cardinal fishes (Apogonidae, Perciformes), with special focus on the adaptive structures of the egg enve
37	Fishelson L, Gon O. 2008 Comparative oogenesis in cardinal fishes (Apogonidae, Perciformes), with special focus on the adaptive structures of the egg enve
38	Neumann D, Woermann D. 2013 Stability of the volume of air trapped on the abdomen of the water spider Argyroneta aquatica. SpringerPlus 2, 694. (doi:10.
39	Mesa ML, Vacchi M. 2005 On the second record of the Antarctic plunderfish Artedidraco glareobarbatus (Artedidraconidae) from the Ross Sea. Polar Biol 29,
40	Mesa ML, Vacchi M. 2005 On the second record of the Antarctic plunderfish Artedidraco glareobarbatus (Artedidraconidae) from the Ross Sea. Polar Biol 29,
41	Fishelson L. 1984 A comparative study of ridge-mazes on surface epithelial cell-membranes of fish scales (Pisces, Teleostei). Zoomorphology 104, 231–238. (
42	Fishelson L. 1984 A comparative study of ridge-mazes on surface epithelial cell-membranes of fish scales (Pisces, Teleostei). Zoomorphology 104, 231–238. (
43	Fishelson L. 1984 A comparative study of ridge-mazes on surface epithelial cell-membranes of fish scales (Pisces, Teleostei). Zoomorphology 104, 231–238. (
44	Fishelson L. 1984 A comparative study of ridge-mazes on surface epithelial cell-membranes of fish scales (Pisces, Teleostei). Zoomorphology 104, 231–238. (
45	Fishelson L. 1984 A comparative study of ridge-mazes on surface epithelial cell-membranes of fish scales (Pisces, Teleostei). Zoomorphology 104, 231–238. (
46	Fishelson L. 1984 A comparative study of ridge-mazes on surface epithelial cell-membranes of fish scales (Pisces, Teleostei). Zoomorphology 104, 231–238. (
47	Pereira A. 2008 A new sclerorhynchiform (Elasmobranchii) from the Middle Cretaceous of Brazil. RBP 11, 207–212. (doi:10.4072/rbp.2008.3.07)
48	Liu G-L, Chang H-K, Chuang Y-C, Lin Y-M, Chen P-Y. 2020 Reversible Underwater Adhesion: The Unique C-shaped Suckers of Net-winged Midge Larvae (Bl
49	Liu G-L, Chang H-K, Chuang Y-C, Lin Y-M, Chen P-Y. 2020 Reversible Underwater Adhesion: The Unique C-shaped Suckers of Net-winged Midge Larvae (Bl
50	Liu G-L, Chang H-K, Chuang Y-C, Lin Y-M, Chen P-Y. 2020 Reversible Underwater Adhesion: The Unique C-shaped Suckers of Net-winged Midge Larvae (Bl
51	Hu W, Zhang J, Kang B. 2016 Structure and function of corneal surface of mudskipper fishes. Fish Physiol Biochem 42, 1481–1489. (doi:10.1007/s10695-01
52	Hu W, Zhang J, Kang B. 2016 Structure and function of corneal surface of mudskipper fishes. Fish Physiol Biochem 42, 1481–1489. (doi:10.1007/s10695-01
53	Hu W, Zhang J, Kang B. 2016 Structure and function of corneal surface of mudskipper fishes. Fish Physiol Biochem 42, 1481–1489. (doi:10.1007/s10695-01
54	Hu W, Zhang J, Kang B. 2016 Structure and function of corneal surface of mudskipper fishes. Fish Physiol Biochem 42, 1481–1489. (doi:10.1007/s10695-01
55	Munuswamy N, Hagiwara A, Murugan G, Hirayama K, Dumont HJ. 1996 Structural differences between the resting eggs of Brachionus plicatilis and Brachionu
56	Munuswamy N, Hagiwara A, Murugan G, Hirayama K, Dumont HJ. 1996 Structural differences between the resting eggs of Brachionus plicatilis and Brachionu
57	Hunter CR, Nayudu PL. 1978 Surface folds in superficial epidermal cells of three species of teleost fish. J Fish Biology 12, 163–166. (doi:10.1111/j.1095-864
58	Hunter CR, Nayudu PL. 1978 Surface folds in superficial epidermal cells of three species of teleost fish. J Fish Biology 12, 163–166. (doi:10.1111/j.1095-864
59	Fishelson L. 1984 A comparative study of ridge-mazes on surface epithelial cell-membranes of fish scales (Pisces, Teleostei). Zoomorphology 104, 231–238. (
60	Fishelson L. 1984 A comparative study of ridge-mazes on surface epithelial cell-membranes of fish scales (Pisces, Teleostei). Zoomorphology 104, 231–238. (
61	Fishelson L. 1984 A comparative study of ridge-mazes on surface epithelial cell-membranes of fish scales (Pisces, Teleostei). Zoomorphology 104, 231–238. (
62	Fishelson L. 1984 A comparative study of ridge-mazes on surface epithelial cell-membranes of fish scales (Pisces, Teleostei). Zoomorphology 104, 231–238. (
63	Fishelson L. 1984 A comparative study of ridge-mazes on surface epithelial cell-membranes of fish scales (Pisces, Teleostei). Zoomorphology 104, 231–238. (
64	Fishelson L. 1984 A comparative study of ridge-mazes on surface epithelial cell-membranes of fish scales (Pisces, Teleostei). Zoomorphology 104, 231–238. (

	A	B	C	D	E	F
65	Aquatic	Animalia	Chordata	Chondrichthyes	Lamniformes	Cetorhinidae
66	Aquatic	Animalia	Chordata	Actinopterygii	Perciformes	Apogonidae
67	Aquatic	Animalia	Chordata	Actinopterygii	Perciformes	Apogonidae
68	Aquatic	Animalia	Chordata	Actinopterygii	Perciformes	Apogonidae
69	Aquatic	Animalia	Platyhelminthes	Trematoda	Diplostomida	Clinostomidae
70	Aquatic	Animalia	Platyhelminthes	Trematoda	Diplostomida	Clinostomidae
71	Aquatic	Animalia	Chordata	Actinopterygii	Aulopiformes	Evermannellidae
72	Aquatic	Animalia	Chordata	Actinopterygii	Aulopiformes	Evermannellidae
73	Aquatic	Animalia	Chordata	Reptilia	Crocodilia	
74	Aquatic	Animalia	Chordata	Mammalia	Cetacea	Monodontidae
75	Aquatic	Animalia	Chordata	Mammalia	Cetacea	Monodontidae
76	Aquatic	Animalia	Arthropoda	Hexanauplia	Siphonostomatoida	Pandaridae
77	Aquatic	Animalia	Arthropoda	Hexanauplia	Siphonostomatoida	Pandaridae
78	Aquatic	Animalia	Arthropoda	Hexanauplia	Siphonostomatoida	Pandaridae
79	Aquatic	Animalia	Arthropoda	Hexanauplia	Siphonostomatoida	Pandaridae
80	Aquatic	Animalia	Arthropoda	Hexanauplia	Siphonostomatoida	Pandaridae
81	Aquatic	Animalia	Arthropoda	Hexanauplia	Siphonostomatoida	Pandaridae
82	Aquatic	Animalia	Arthropoda	Hexanauplia	Siphonostomatoida	Pandaridae
83	Aquatic	Animalia	Arthropoda	Hexanauplia	Siphonostomatoida	Pandaridae
84	Aquatic	Animalia	Arthropoda	Hexanauplia	Siphonostomatoida	Pandaridae
85	Aquatic	Animalia	Arthropoda	Hexanauplia	Siphonostomatoida	Pandaridae
86	Aquatic	Animalia	Arthropoda	Hexanauplia	Siphonostomatoida	Pandaridae
87	Aquatic	Animalia	Chordata	Actinopterygii	Perciformes	Sparidae
88	Aquatic	Animalia	Chordata	Actinopterygii	Perciformes	Sparidae
89	Aquatic	Animalia	Chordata	Actinopterygii	Perciformes	Sparidae
90	Aquatic	Animalia	Chordata	Actinopterygii	Perciformes	Nototheneidae
91	Aquatic	Animalia	Arthropoda	Hexanauplia	Siphonostomatoida	Pandaridae
92	Aquatic	Animalia	Arthropoda	Hexanauplia	Siphonostomatoida	Pandaridae
93	Aquatic	Animalia	Arthropoda	Hexanauplia	Siphonostomatoida	Pandaridae
94	Aquatic	Animalia	Arthropoda	Hexanauplia	Siphonostomatoida	Pandaridae
95	Aquatic	Animalia	Arthropoda	Hexanauplia	Siphonostomatoida	Pandaridae

	G	H	I	J	K	L	M
65	*Cetorhinus maximus*	Raker		Longitudinal	1000-10000	10-100	Flow control
66	*Cheilodipterus lineatus*	Egg	Adult	Maze	100-1000	1-10	Adhesion and friction
67	*Cheilodipterus lineatus*	Egg	Adult	Maze	100-1000	1-10	Chanelling
68	*Cheilodipterus lineatus*	Egg	Adult	Maze	100-1000	1-10	Structural support
69	*Clinostomum complanatum*	Skin	Larvae	Parallel and labyrinth	1-10	1-10	Stretching
70	*Clinostomum complanatum*	Skin	Larvae	Parallel and labyrinth	1-10	1-10	Nutrient intake
71	*Coccorella atlantica*	Esophagus	Adult	Maze	100-1000	0.1-1	Chanelling
72	*Coccorella atlantica*	Esophagus	Adult	Maze	100-1000	0.1-1	Fluid retention
73	*Deinosuchus rugosus*	Tooth	Adult	Parallel	1000-10000	100-1000	Feeding
74	*Delphinapterus leucas*	Skin	Adult	Parallel	1000-10000	10-100	Flow control
75	*Delphinapterus leucas*	Skin	Adult	Parallel	1000-10000	10-100	Sensing
76	*Dinemoura latifolia*	Antenna	Adult	Parallel	100-1000	1-10	
77	*Dinemoura latifolia*	Antenna	Adult	Parallel	100-1000	1-10	
78	*Dinemoura latifolia*	Antenna	Adult	Parallel	100-1000	1-10	
79	*Dinemoura latifolia*	Maxilliped	Adult	Parallel	100-1000	1-10	
80	*Dinemoura latifolia*		Adult	Parallel	100-1000	1-10	
81	*Dinemoura latifolia*	Leg	Adult	Parallel	100-1000	1-10	
82	*Dinemoura latifolia*	Leg	Adult	Parallel	100-1000	1-10	
83	*Dinemoura latifolia*	Leg	Adult	Parallel	100-1000	1-10	
84	*Dinemoura latifolia*	Leg	Adult	Parallel	100-1000	1-10	
85	*Dinemoura latifolia*	Leg	Adult	Parallel	100-1000	1-10	
86	*Dinemoura latifolia*	Leg	Adult	Parallel	100-1000	1-10	
87	*Diplodus annularis*	Scale		Maze	100-1000	0.1-1	Flow control
88	*Diplodus annularis*	Scale		Maze	100-1000	0.1-1	Fluid retention
89	*Diplodus annularis*	Scale		Maze	100-1000	0.1-1	Structural support
90	*Dissostichus eleginoides*	Tooth		Infoldings	1000-10000	100-1000	Structural support
91	*Echthrogaleus coleoptratus*	Antenna	Adult	Parallel	100-1000	1-10	
92	*Echthrogaleus coleoptratus*	Antenna	Adult	Parallel	100-1000	1-10	
93	*Echthrogaleus coleoptratus*	Maxilliped	Adult	Parallel	100-1000	1-10	
94	*Echthrogaleus coleoptratus*	Leg	Adult	Parallel	100-1000	1-10	
95	*Echthrogaleus coleoptratus*	Leg	Adult	Parallel	100-1000	1-10	

	N
65	Own work
66	Fishelson L, Gon O. 2008 Comparative oogenesis in cardinal fishes (Apogonidae, Perciformes), with special focus on the adaptive structures of the egg enve
67	Fishelson L, Gon O. 2008 Comparative oogenesis in cardinal fishes (Apogonidae, Perciformes), with special focus on the adaptive structures of the egg enve
68	Fishelson L, Gon O. 2008 Comparative oogenesis in cardinal fishes (Apogonidae, Perciformes), with special focus on the adaptive structures of the egg enve
69	Abidi SMA, Ahmad M, Nizami WA, Hanna REB. 1988 Clinostomum complanatum: Tegumental surface changes during in vivo development. International Jour
70	Abidi SMA, Ahmad M, Nizami WA, Hanna REB. 1988 Clinostomum complanatum: Tegumental surface changes during in vivo development. International Jour
71	Sperry DG, Wassersug RJ. 1976 A proposed function for microridges on epithelial cells. Anat. Rec. 185, 253–257. (doi:10.1002/ar.1091850212)
72	Sperry DG, Wassersug RJ. 1976 A proposed function for microridges on epithelial cells. Anat. Rec. 185, 253–257. (doi:10.1002/ar.1091850212)
73	McCurry MR, Evans AR, Fitzgerald EMG, McHenry CR, Bevitt J, Pyenson ND. 2019 The repeated evolution of dental apicobasal ridges in aquatic-feeding mar
74	Wainwright DK, Fish FE, Ingersoll S, Williams TM, St Leger J, Smits AJ, Lauder GV. 2019 How smooth is a dolphin? The ridged skin of odontocetes. Biol. Lett.
75	Wainwright DK, Fish FE, Ingersoll S, Williams TM, St Leger J, Smits AJ, Lauder GV. 2019 How smooth is a dolphin? The ridged skin of odontocetes. Biol. Lett.
76	Ingram AL, Parker AR. 2006 The functional morphology and attachment mechanism of pandarid adhesion pads (Crustacea: Copepoda: Pandaridae). Zoologi
77	Ingram AL, Parker AR. 2006 The functional morphology and attachment mechanism of pandarid adhesion pads (Crustacea: Copepoda: Pandaridae). Zoologi
78	Ingram AL, Parker AR. 2006 The functional morphology and attachment mechanism of pandarid adhesion pads (Crustacea: Copepoda: Pandaridae). Zoologi
79	Ingram AL, Parker AR. 2006 The functional morphology and attachment mechanism of pandarid adhesion pads (Crustacea: Copepoda: Pandaridae). Zoologi
80	Ingram AL, Parker AR. 2006 The functional morphology and attachment mechanism of pandarid adhesion pads (Crustacea: Copepoda: Pandaridae). Zoologi
81	Ingram AL, Parker AR. 2006 The functional morphology and attachment mechanism of pandarid adhesion pads (Crustacea: Copepoda: Pandaridae). Zoologi
82	Ingram AL, Parker AR. 2006 The functional morphology and attachment mechanism of pandarid adhesion pads (Crustacea: Copepoda: Pandaridae). Zoologi
83	Ingram AL, Parker AR. 2006 The functional morphology and attachment mechanism of pandarid adhesion pads (Crustacea: Copepoda: Pandaridae). Zoologi
84	Ingram AL, Parker AR. 2006 The functional morphology and attachment mechanism of pandarid adhesion pads (Crustacea: Copepoda: Pandaridae). Zoologi
85	Ingram AL, Parker AR. 2006 The functional morphology and attachment mechanism of pandarid adhesion pads (Crustacea: Copepoda: Pandaridae). Zoologi
86	Ingram AL, Parker AR. 2006 The functional morphology and attachment mechanism of pandarid adhesion pads (Crustacea: Copepoda: Pandaridae). Zoologi
87	Fishelson L. 1984 A comparative study of ridge-mazes on surface epithelial cell-membranes of fish scales (Pisces, Teleostei). Zoomorphology 104, 231–238. (
88	Fishelson L. 1984 A comparative study of ridge-mazes on surface epithelial cell-membranes of fish scales (Pisces, Teleostei). Zoomorphology 104, 231–238. (
89	Fishelson L. 1984 A comparative study of ridge-mazes on surface epithelial cell-membranes of fish scales (Pisces, Teleostei). Zoomorphology 104, 231–238. (
90	Germain D, Mondéjar-Fernández J, Meunier FJ. 2016 The detection of weakly developed plicidentine in teleost teeth using 3D tomography. (doi:10.26028/CY
91	Ingram AL, Parker AR. 2006 The functional morphology and attachment mechanism of pandarid adhesion pads (Crustacea: Copepoda: Pandaridae). Zoologi
92	Ingram AL, Parker AR. 2006 The functional morphology and attachment mechanism of pandarid adhesion pads (Crustacea: Copepoda: Pandaridae). Zoologi
93	Ingram AL, Parker AR. 2006 The functional morphology and attachment mechanism of pandarid adhesion pads (Crustacea: Copepoda: Pandaridae). Zoologi
94	Ingram AL, Parker AR. 2006 The functional morphology and attachment mechanism of pandarid adhesion pads (Crustacea: Copepoda: Pandaridae). Zoologi
95	Ingram AL, Parker AR. 2006 The functional morphology and attachment mechanism of pandarid adhesion pads (Crustacea: Copepoda: Pandaridae). Zoologi

	A	B	C	D	E	F
96	Aquatic	Animalia	Arthropoda	Hexanauplia	Siphonostomatoida	Pandaridae
97	Aquatic	Animalia	Arthropoda	Hexanauplia	Siphonostomatoida	Pandaridae
98	Aquatic	Animalia	Arthropoda	Hexanauplia	Siphonostomatoida	Pandaridae
99	Aquatic	Animalia	Arthropoda	Hexanauplia	Siphonostomatoida	Pandaridae
100	Aquatic	Animalia	Arthropoda	Hexanauplia	Siphonostomatoida	Pandaridae
101	Aquatic	Animalia	Arthropoda	Hexanauplia	Siphonostomatoida	Pandaridae
102	Aquatic	Animalia	Arthropoda	Hexanauplia	Siphonostomatoida	Pandaridae
103	Aquatic	Animalia	Arthropoda	Hexanauplia	Siphonostomatoida	Pandaridae
104	Aquatic	Animalia	Arthropoda	Hexanauplia	Siphonostomatoida	Pandaridae
105	Aquatic	Excavata	Euglenophyta	Euglenophyceae	Euglenales	Euglenaceae
106	Aquatic	Excavata	Euglenophyta	Euglenophyceae	Euglenales	Euglenaceae
107	Aquatic	Animalia	Chordata	Actinopterygii	Scorpaeniformes	Cyclopteridae
108	Aquatic	Animalia	Chordata	Actinopterygii	Perciformes	Kyphosidae
109	Aquatic	Animalia	Chordata	Actinopterygii	Perciformes	Kyphosidae
110	Aquatic	Animalia	Chordata	Actinopterygii	Perciformes	Kyphosidae
111	Aquatic	Animalia	Chordata	Mammalia	Cetacea	Delphinidae
112	Aquatic	Animalia	Chordata	Mammalia	Cetacea	Delphinidae
113	Aquatic	Animalia	Chordata	Reptilia	Squamata	Mosasauridae
114	Aquatic	Animalia	Chordata	Actinopterygii	Perciformes	Apogonidae
115	Aquatic	Animalia	Chordata	Actinopterygii	Perciformes	Apogonidae
116	Aquatic	Animalia	Chordata	Actinopterygii	Perciformes	Apogonidae
117	Aquatic	Animalia	Chordata	Reptilia	Squamata	Colubridae
118	Aquatic	Animalia	Chordata	Reptilia	Squamata	Colubridae
119	Aquatic	Animalia	Chordata	Reptilia	Squamata	Homalopsidae
120	Aquatic	Animalia	Chordata	Reptilia	Squamata	Homalopsidae
121	Aquatic	Animalia	Chordata	Actinopterygii	Characiformes	Erythrinidae
122	Aquatic	Animalia	Chordata	Actinopterygii	Characiformes	Alestidae
123	Aquatic	Animalia	Chordata	Mammalia	Carnivora	Phocidae
124	Aquatic	Animalia	Chordata	Actinopterygii	Ovalentaria *incertae sedis*	Pomacentridae
125	Aquatic	Animalia	Chordata	Actinopterygii	Ovalentaria *incertae sedis*	Pomacentridae
126	Aquatic	Animalia	Chordata	Actinopterygii	Ovalentaria *incertae sedis*	Pomacentridae
127	Aquatic	Animalia	Chordata	Actinopterygii	Perciformes	Gobiidae
128	Aquatic	Animalia	Chordata	Actinopterygii	Perciformes	Gobiidae

	G	H	I	J	K	L	M
96	*Echthrogaleus coleoptratus*	Leg	Adult	Parallel	100-1000	1-10	
97	*Echthrogaleus coleoptratus*	Leg	Adult	Parallel	100-1000	1-10	
98	*Echthrogaleus coleoptratus*	Leg	Adult	Parallel	100-1000	1-10	
99	*Echthrogaleus coleoptratus*	Leg	Adult	Parallel	100-1000	1-10	
100	*Echthrogaleus coleoptratus*	Leg	Adult	Parallel	100-1000	1-10	
101	*Echthrogaleus coleoptratus*	Leg	Adult	Parallel	100-1000	1-10	
102	*Echthrogaleus coleoptratus*	Leg	Adult	Parallel	100-1000	1-10	
103	*Echthrogaleus coleoptratus*	Leg	Adult	Parallel	100-1000	1-10	
104	*Echthrogaleus coleoptratus*	Leg	Adult	Parallel	100-1000	1-10	
105	*Euglena helicoideus*	Skin	Adult	Parallel/twisted	0.1-1		Chanelling
106	*Euglena helicoideus*	Skin	Adult	Parallel/twisted	0.1-1		Friction and Wear reduction
107	*Eumicrotremus orbis*	Integument		Radial	10-100	10-100	
108	*Girella nigricans*	Scale		Maze	100-1000	0.1-1	Flow control
109	*Girella nigricans*	Scale		Maze	100-1000	0.1-1	Fluid retention
110	*Girella nigricans*	Scale		Maze	100-1000	0.1-1	Structural support
111	*Globicephala macrorhynchus*	Skin	Adult	Parallel	1000-10000	1-10	Flow control
112	*Globicephala macrorhynchus*	Skin	Adult	Parallel	1000-10000	1-10	Sensing
113	*Globidens alabamensis*	Tooth	Adult	Parallel	1000-10000	100-1000	Feeding
114	*Glossamia aprion*	Egg	Adult	Maze	10-100		Adhesion and friction
115	*Glossamia aprion*	Egg	Adult	Maze	10-100		Chanelling
116	*Glossamia aprion*	Egg	Adult	Maze	10-100		Structural support
117	*Helicops leopardinus*	Tooth	Adult	Parallel		10-100	Drag reduction
118	*Helicops leopardinus*	Tooth	Adult	Parallel		10-100	Structural support
119	*Homalapsis buccata*	Tooth	Adult	Parallel	100-1000	10-100	Drag reduction
120	*Homalapsis buccata*	Tooth	Adult	Parallel	100-1000	10-100	Structural support
121	*Hoplias aimara*	Tooth	Adult	Infoldings	1000-10000	100-1000	Structural support
122	*Hydrocynus forskahlii.*	Tooth	Adult	Infoldings	100-1000		Structural support
123	*Hydrurga leptonyx*	Tooth	Adult	Parallel	1000-10000		Feeding
124	*Hypsypops rubieunda*	Scale		Maze	100-1000	0.1-1	Flow control
125	*Hypsypops rubieunda*	Scale		Maze	100-1000	0.1-1	Fluid retention
126	*Hypsypops rubieunda*	Scale		Maze	100-1000	0.1-1	Structural support
127	*Istigobius spence*	Scale		Maze		0.1-1	Flow control
128	*Istigobius spence*	Scale		Maze		0.1-1	Fluid retention

	N
96	Ingram AL, Parker AR. 2006 The functional morphology and attachment mechanism of pandarid adhesion pads (Crustacea: Copepoda: Pandaridae). Zoologi
97	Ingram AL, Parker AR. 2006 The functional morphology and attachment mechanism of pandarid adhesion pads (Crustacea: Copepoda: Pandaridae). Zoologi
98	Ingram AL, Parker AR. 2006 The functional morphology and attachment mechanism of pandarid adhesion pads (Crustacea: Copepoda: Pandaridae). Zoologi
99	Ingram AL, Parker AR. 2006 The functional morphology and attachment mechanism of pandarid adhesion pads (Crustacea: Copepoda: Pandaridae). Zoologi
100	Ingram AL, Parker AR. 2006 The functional morphology and attachment mechanism of pandarid adhesion pads (Crustacea: Copepoda: Pandaridae). Zoologi
101	Ingram AL, Parker AR. 2006 The functional morphology and attachment mechanism of pandarid adhesion pads (Crustacea: Copepoda: Pandaridae). Zoologi
102	Ingram AL, Parker AR. 2006 The functional morphology and attachment mechanism of pandarid adhesion pads (Crustacea: Copepoda: Pandaridae). Zoologi
103	Ingram AL, Parker AR. 2006 The functional morphology and attachment mechanism of pandarid adhesion pads (Crustacea: Copepoda: Pandaridae). Zoologi
104	Ingram AL, Parker AR. 2006 The functional morphology and attachment mechanism of pandarid adhesion pads (Crustacea: Copepoda: Pandaridae). Zoologi
105	Hunter CR, Nayudu PL. 1978 Surface folds in superficial epidermal cells of three species of teleost fish. J Fish Biology 12, 163–166. (doi:10.1111/j.1095-864
106	Hunter CR, Nayudu PL. 1978 Surface folds in superficial epidermal cells of three species of teleost fish. J Fish Biology 12, 163–166. (doi:10.1111/j.1095-864
107	Woodruff EC, Huie JM, Summers AP, Cohen KE. 2022 Pacific Spiny Lumpsucker armor—Development, damage, and defense in the intertidal. Journal of Mor
108	Fishelson L. 1984 A comparative study of ridge-mazes on surface epithelial cell-membranes of fish scales (Pisces, Teleostei). Zoomorphology 104, 231–238.
109	Fishelson L. 1984 A comparative study of ridge-mazes on surface epithelial cell-membranes of fish scales (Pisces, Teleostei). Zoomorphology 104, 231–238.
110	Fishelson L. 1984 A comparative study of ridge-mazes on surface epithelial cell-membranes of fish scales (Pisces, Teleostei). Zoomorphology 104, 231–238.
111	Wainwright DK, Fish FE, Ingersoll S, Williams TM, St Leger J, Smits AJ, Lauder GV. 2019 How smooth is a dolphin? The ridged skin of odontocetes. Biol. Lett
112	Wainwright DK, Fish FE, Ingersoll S, Williams TM, St Leger J, Smits AJ, Lauder GV. 2019 How smooth is a dolphin? The ridged skin of odontocetes. Biol. Lett
113	McCurry MR, Evans AR, Fitzgerald EMG, McHenry CR, Bevitt J, Pyenson ND. 2019 The repeated evolution of dental apicobasal ridges in aquatic-feeding mar
114	Fishelson L, Gon O. 2008 Comparative oogenesis in cardinal fishes (Apogonidae, Perciformes), with special focus on the adaptive structures of the egg enve
115	Fishelson L, Gon O. 2008 Comparative oogenesis in cardinal fishes (Apogonidae, Perciformes), with special focus on the adaptive structures of the egg enve
116	Fishelson L, Gon O. 2008 Comparative oogenesis in cardinal fishes (Apogonidae, Perciformes), with special focus on the adaptive structures of the egg enve
117	Vaeth RH, Rossman DA, Shoop W. 1985 Observations of Tooth Surface Morphology in Snakes. Journal of Herpetology 19, 20. (doi:10.2307/1564416)
118	Vaeth RH, Rossman DA, Shoop W. 1985 Observations of Tooth Surface Morphology in Snakes. Journal of Herpetology 19, 20. (doi:10.2307/1564416)
119	Vaeth RH, Rossman DA, Shoop W. 1985 Observations of Tooth Surface Morphology in Snakes. Journal of Herpetology 19, 20. (doi:10.2307/1564416)
120	Vaeth RH, Rossman DA, Shoop W. 1985 Observations of Tooth Surface Morphology in Snakes. Journal of Herpetology 19, 20. (doi:10.2307/1564416)
121	Meunier FJ, De Mayrinck D, Brito PM. 2015 Presence of plicidentine in the labial teeth of Hoplias aimara (Erythrinidae; Ostariophysi; Teleostei). Acta Zool 96,
122	Meunier FJ, De Mayrinck D, Brito PM. 2015 Presence of plicidentine in the labial teeth of Hoplias aimara (Erythrinidae; Ostariophysi; Teleostei). Acta Zool 96,
123	McCurry MR, Evans AR, Fitzgerald EMG, McHenry CR, Bevitt J, Pyenson ND. 2019 The repeated evolution of dental apicobasal ridges in aquatic-feeding mar
124	Fishelson L. 1984 A comparative study of ridge-mazes on surface epithelial cell-membranes of fish scales (Pisces, Teleostei). Zoomorphology 104, 231–238.
125	Fishelson L. 1984 A comparative study of ridge-mazes on surface epithelial cell-membranes of fish scales (Pisces, Teleostei). Zoomorphology 104, 231–238.
126	Fishelson L. 1984 A comparative study of ridge-mazes on surface epithelial cell-membranes of fish scales (Pisces, Teleostei). Zoomorphology 104, 231–238.
127	Fishelson L. 1984 A comparative study of ridge-mazes on surface epithelial cell-membranes of fish scales (Pisces, Teleostei). Zoomorphology 104, 231–238.
128	Fishelson L. 1984 A comparative study of ridge-mazes on surface epithelial cell-membranes of fish scales (Pisces, Teleostei). Zoomorphology 104, 231–238.

	A	B	C	D	E	F
129	Aquatic	Animalia	Chordata	Actinopterygii	Perciformes	Gobiidae
130	Aquatic	Animalia	Chordata	Mammalia	Cetacea	Delphinidae
131	Aquatic	Animalia	Chordata	Mammalia	Cetacea	Delphinidae
132	Aquatic	Animalia	Chordata	Sarcopterygii	Coelacanthiformes	Latimeriidae
133	Aquatic	Animalia	Chordata	Actinopterygii	Cyprinodontiformes	Poeciliidae
134	Aquatic	Animalia	Chordata	Actinopterygii	Cyprinodontiformes	Poeciliidae
135	Aquatic	Animalia	Chordata	Actinopterygii	Cypriniformes	Cobitidae
136	Aquatic	Animalia	Chordata	Actinopterygii	Lepisosteiformes	Lepisosteidae
137	Aquatic	Animalia	Chordata	Actinopterygii	Perciformes	Centrarchidae
138	Aquatic	Animalia	Chordata	Actinopterygii	Perciformes	Centrarchidae
139	Aquatic	Animalia	Chordata	Actinopterygii	Perciformes	Centrarchidae
140	Aquatic	Animalia	Chordata	Actinopterygii	Perciformes	Centrarchidae
141	Aquatic	Animalia	Chordata	Actinopterygii	Perciformes	Centrarchidae
142	Aquatic	Animalia	Chordata	Actinopterygii	Perciformes	Centrarchidae
143	Aquatic	Animalia	Chordata	Actinopterygii	Perciformes	Centrarchidae
144	Aquatic	Animalia	Chordata	Actinopterygii	Perciformes	Centrarchidae
145	Aquatic	Animalia	Chordata	Actinopterygii	Lophiiformes	Lophiidae
146	Aquatic	Animalia	Chordata	Actinopterygii	Perciformes	Moronidae
147	Aquatic	Animalia	Chordata	Actinopterygii	Perciformes	Moronidae
148	Aquatic	Animalia	Chordata	Actinopterygii	Perciformes	Moronidae
149	Aquatic	Animalia	Chordata	Actinopterygii	Perciformes	Moronidae
150	Aquatic	Animalia	Chordata	Actinopterygii	Perciformes	Moronidae
151	Aquatic	Animalia	Chordata	Actinopterygii	Perciformes	Moronidae
152	Aquatic	Animalia	Chordata	Actinopterygii	Mugiliformes	Mugilidae
153	Aquatic	Animalia	Mollusca	Bivalvia	Mytilida	Mytilidae
154	Aquatic	Animalia	Chordata	Mammalia	Cetacea	Delphinidae
155	Aquatic	Animalia	Chordata	Mammalia	Cetacea	Delphinidae
156	Aquatic	Animalia	Chordata	Actinopterygii	Cichliformes	Cichlidae
157	Aquatic	Animalia	Chordata	Actinopterygii	Cichliformes	Cichlidae
158	Aquatic	Animalia	Chordata	Actinopterygii	Cichliformes	Cichlidae
159	Aquatic	Animalia	Chordata	Actinopterygii	Perciformes	Cichlidae
160	Aquatic	Animalia	Chordata	Actinopterygii	Perciformes	Cichlidae
161	Aquatic	Animalia	Chordata	Actinopterygii	Perciformes	Cichlidae

	G	H	I	J	K	L	M
129	*Istigobius spence*	Scale		Maze		0.1-1	Structural support
130	*Lagenorhynchus obliquiden*	Skin	Adult	Parallel	1000-10000	10-100	Flow control
131	*Lagenorhynchus obliquiden*	Skin	Adult	Parallel	1000-10000	10-100	Sensing
132	*Latimeria chalumnae*	Tooth	Adult	Infoldings	1000-10000		Structural support
133	*Lebistes reticulatus*	Skin		Maze	10-100	1-10	Fluid exchange
134	*Lebistes reticulatus*	Skin		Maze	10-100	1-10	Fluid retention
135	*Lepidocephalichthys guntea*	Integument		Maze	100-1000	0.1-1	Structural support
136	*Lepisosteus platostomus*	Tooth		Infoldings	100-1000	100-1000	Structural support
137	*Lepomis gibbosus*	Skin		Labyrinth	1-10	0.1-1	Fluid retention
138	*Lepomis gibbosus*	Skin		Maze	10-100	0.1-1	Fluid retention
139	*Lepomis gibbosus*	Skin		Maze	10-100	0.1-1	Fluid retention
140	*Lepomis gibbosus*	Skin		Maze	100-1000	0.1-1	Fluid retention
141	*Lepomis gibbosus*	Skin		Labyrinth	1-10	0.1-1	Friction and Wear reduction
142	*Lepomis gibbosus*	Skin		Maze	10-100	0.1-1	Friction and Wear reduction
143	*Lepomis gibbosus*	Skin		Maze	10-100	0.1-1	Friction and Wear reduction
144	*Lepomis gibbosus*	Skin		Maze	100-1000	0.1-1	Friction and Wear reduction
145	*Lophius sp*	Tooth	Adult	Infoldings	1000-10000		Structural support
146	*Morone saxatilis*	Scale		Maze	1000-10000	0.1-1	Flow control
147	*Morone saxatilis*	Scale		Maze	1000-10000	0.1-1	Flow control
148	*Morone saxatilis*	Scale		Maze	1000-10000	0.1-1	Fluid retention
149	*Morone saxatilis*	Scale		Maze	1000-10000	0.1-1	Fluid retention
150	*Morone saxatilis*	Scale		Maze	1000-10000	0.1-1	Structural support
151	*Morone saxatilis*	Scale		Maze	1000-10000	0.1-1	Structural support
152	*Mugil cephalus*	Gill arch	Adult	Labyrinth	1000-10000	0.1-1	Fluid retention
153	*Mytilus trossulus*	Byssal thread	Adult	Wavy	10-100	1-10	Stretching
154	*Orcinus orca*	Skin	Adult	Parallel	1000-10000	10-100	Flow control
155	*Orcinus orca*	Skin	Adult	Parallel	1000-10000	10-100	Sensing
156	Oreochromis aureus	Scale		Maze	100-1000	0.1-1	Flow control
157	Oreochromis aureus	Scale		Maze	100-1000	0.1-1	Fluid retention
158	Oreochromis aureus	Scale		Maze	100-1000	0.1-1	Structural support
159	*Oreochromis aureus*	Scale		Maze	100-1000	0.1-1	Flow control
160	*Oreochromis aureus*	Scale		Maze	100-1000	0.1-1	Fluid retention
161	*Oreochromis aureus*	Scale		Maze	100-1000	0.1-1	Structural support

	N
129	Fishelson L. 1984 A comparative study of ridge-mazes on surface epithelial cell-membranes of fish scales (Pisces, Teleostei). Zoomorphology 104, 231–238. (
130	Wainwright DK, Fish FE, Ingersoll S, Williams TM, St Leger J, Smits AJ, Lauder GV. 2019 How smooth is a dolphin? The ridged skin of odontocetes. Biol. Lett.
131	Wainwright DK, Fish FE, Ingersoll S, Williams TM, St Leger J, Smits AJ, Lauder GV. 2019 How smooth is a dolphin? The ridged skin of odontocetes. Biol. Lett.
132	Meunier FJ, Mondéjar-Fernández J, Goussard F, Clément G, Herbin M. 2015 Presence of plicidentine in the oral teeth of the coelacanth Latimeria chalumnae
133	Schliwa M. 1975 Cytoarchitecture of surface layer cells of the teleost epidermis. Journal of Ultrastructure Research 52, 377–386. (doi:10.1016/S0022-5320(75
134	Schliwa M. 1975 Cytoarchitecture of surface layer cells of the teleost epidermis. Journal of Ultrastructure Research 52, 377–386. (doi:10.1016/S0022-5320(75
135	Mittal S, Pinky, Mittal AK. 2004 Operculum of peppered loach, Lepidocephalichthys guntea (Hamilton, 1822) (Cobitidae, Cypriniformes): a scanning electron m
136	Germain D, Mondéjar-Fernández J, Meunier FJ. 2016 The detection of weakly developed plicidentine in teleost teeth using 3D tomography. (doi:10.26028/CY
137	DePasquale JA. 2020 Comparison of microridges in juvenile and adult sunfish, Lepomis gibbosus. Acta Zool 101, 147–155. (doi:10.1111/azo.12281)
138	DePasquale JA. 2020 Comparison of microridges in juvenile and adult sunfish, Lepomis gibbosus. Acta Zool 101, 147–155. (doi:10.1111/azo.12281)
139	DePasquale JA. 2020 Comparison of microridges in juvenile and adult sunfish, Lepomis gibbosus. Acta Zool 101, 147–155. (doi:10.1111/azo.12281)
140	DePasquale JA. 2020 Comparison of microridges in juvenile and adult sunfish, Lepomis gibbosus. Acta Zool 101, 147–155. (doi:10.1111/azo.12281)
141	DePasquale JA. 2020 Comparison of microridges in juvenile and adult sunfish, Lepomis gibbosus. Acta Zool 101, 147–155. (doi:10.1111/azo.12281)
142	DePasquale JA. 2020 Comparison of microridges in juvenile and adult sunfish, Lepomis gibbosus. Acta Zool 101, 147–155. (doi:10.1111/azo.12281)
143	DePasquale JA. 2020 Comparison of microridges in juvenile and adult sunfish, Lepomis gibbosus. Acta Zool 101, 147–155. (doi:10.1111/azo.12281)
144	DePasquale JA. 2020 Comparison of microridges in juvenile and adult sunfish, Lepomis gibbosus. Acta Zool 101, 147–155. (doi:10.1111/azo.12281)
145	Meunier FJ, De Mayrinck D, Brito PM. 2015 Presence of plicidentine in the labial teeth of Hoplias aimara (Erythrinidae; Ostariophysi; Teleostei). Acta Zool 96, 1
146	Fishelson L. 1984 A comparative study of ridge-mazes on surface epithelial cell-membranes of fish scales (Pisces, Teleostei). Zoomorphology 104, 231–238. (
147	Fishelson L. 1984 A comparative study of ridge-mazes on surface epithelial cell-membranes of fish scales (Pisces, Teleostei). Zoomorphology 104, 231–238. (
148	Fishelson L. 1984 A comparative study of ridge-mazes on surface epithelial cell-membranes of fish scales (Pisces, Teleostei). Zoomorphology 104, 231–238. (
149	Fishelson L. 1984 A comparative study of ridge-mazes on surface epithelial cell-membranes of fish scales (Pisces, Teleostei). Zoomorphology 104, 231–238. (
150	Fishelson L. 1984 A comparative study of ridge-mazes on surface epithelial cell-membranes of fish scales (Pisces, Teleostei). Zoomorphology 104, 231–238. (
151	Fishelson L. 1984 A comparative study of ridge-mazes on surface epithelial cell-membranes of fish scales (Pisces, Teleostei). Zoomorphology 104, 231–238. (
152	Hossler FE. 1980 Gill arch of the mullet, Mugil cephalus III. Rate of response to salinity change. American Journal of Physiology-Regulatory, Integrative and C
153	Vekhova EE. 2021 The Byssal Apparatus in the Pacific Mussel, Mytilus trossulus (Bivalvia, Mytilidae), from the Sea of Japan. Biol Bull Russ Acad Sci 48, 1443
154	Wainwright DK, Fish FE, Ingersoll S, Williams TM, St Leger J, Smits AJ, Lauder GV. 2019 How smooth is a dolphin? The ridged skin of odontocetes. Biol. Lett.
155	Wainwright DK, Fish FE, Ingersoll S, Williams TM, St Leger J, Smits AJ, Lauder GV. 2019 How smooth is a dolphin? The ridged skin of odontocetes. Biol. Lett.
156	Fishelson L. 1984 A comparative study of ridge-mazes on surface epithelial cell-membranes of fish scales (Pisces, Teleostei). Zoomorphology 104, 231–238. (
157	Fishelson L. 1984 A comparative study of ridge-mazes on surface epithelial cell-membranes of fish scales (Pisces, Teleostei). Zoomorphology 104, 231–238. (
158	Fishelson L. 1984 A comparative study of ridge-mazes on surface epithelial cell-membranes of fish scales (Pisces, Teleostei). Zoomorphology 104, 231–238. (
159	Fishelson L. 1984 A comparative study of ridge-mazes on surface epithelial cell-membranes of fish scales (Pisces, Teleostei). Zoomorphology 104, 231–238. (
160	Fishelson L. 1984 A comparative study of ridge-mazes on surface epithelial cell-membranes of fish scales (Pisces, Teleostei). Zoomorphology 104, 231–238. (
161	Fishelson L. 1984 A comparative study of ridge-mazes on surface epithelial cell-membranes of fish scales (Pisces, Teleostei). Zoomorphology 104, 231–238. (

	A	B	C	D	E	F
162	Aquatic	Animalia	Chordata	Actinopterygii	Perciformes	Cichlidae
163	Aquatic	Animalia	Chordata	Actinopterygii	Perciformes	Cichlidae
164	Aquatic	Animalia	Chordata	Actinopterygii	Perciformes	Cichlidae
165	Aquatic	Animalia	Arthropoda	Hexanauplia	Siphonostomatoida	Pandaridae
166	Aquatic	Animalia	Arthropoda	Hexanauplia	Siphonostomatoida	Pandaridae
167	Aquatic	Animalia	Arthropoda	Hexanauplia	Siphonostomatoida	Pandaridae
168	Aquatic	Animalia	Arthropoda	Hexanauplia	Siphonostomatoida	Pandaridae
169	Aquatic	Animalia	Arthropoda	Hexanauplia	Siphonostomatoida	Pandaridae
170	Aquatic	Animalia	Arthropoda	Hexanauplia	Siphonostomatoida	Pandaridae
171	Aquatic	Animalia	Arthropoda	Hexanauplia	Siphonostomatoida	Pandaridae
172	Aquatic	Animalia	Arthropoda	Hexanauplia	Siphonostomatoida	Pandaridae
173	Aquatic	Animalia	Arthropoda	Hexanauplia	Siphonostomatoida	Pandaridae
174	Aquatic	Animalia	Arthropoda	Hexanauplia	Siphonostomatoida	Pandaridae
175	Aquatic	Animalia	Arthropoda	Hexanauplia	Siphonostomatoida	Pandaridae
176	Aquatic	Animalia	Arthropoda	Hexanauplia	Siphonostomatoida	Pandaridae
177	Aquatic	Animalia	Platyhelminthes	Monogenea	Microcotylidae	Monogenea
178	Aquatic	Animalia	Platyhelminthes	Monogenea	Microcotylidae	Monogenea
179	Aquatic	Animalia	Mollusca	Gastropoda	Archaeogastropoda	Patellidae
180	Aquatic	Animalia	Chordata	Actinopterygii	Perciformes	Oxudercidae
181	Aquatic	Animalia	Chordata	Actinopterygii	Perciformes	Oxudercidae
182	Aquatic	Animalia	Chordata	Actinopterygii	Perciformes	Oxudercidae
183	Aquatic	Animalia	Chordata	Actinopterygii	Perciformes	Oxudercidae
184	Aquatic	Animalia	Chordata	Actinopterygii	Perciformes	Apogonidae
185	Aquatic	Animalia	Chordata	Actinopterygii	Perciformes	Apogonidae
186	Aquatic	Animalia	Chordata	Actinopterygii	Perciformes	Apogonidae
187	Aquatic	Animalia	Chordata	Actinopterygii	Siluriformes	Pimelodidae
188	Aquatic	Animalia	Chordata	Actinopterygii	Cyprinodontiformes	Poeciliidae
189	Aquatic	Animalia	Chordata	Actinopterygii	Cyprinodontiformes	Poeciliidae
190	Aquatic	Animalia	Chordata	Actinopterygii	Cyprinodontiformes	Poeciliidae
191	Aquatic	Animalia	Chordata	Actinopterygii	Cyprinodontiformes	Poeciliidae
192	Aquatic	Animalia	Chordata	Actinopterygii	Perciformes	Cichlidae
193	Aquatic	Animalia	Chordata	Actinopterygii	Perciformes	Cichlidae
194	Aquatic	Animalia	Chordata	Actinopterygii	Perciformes	Cichlidae

	G	H	I	J	K	L	M
162	*Oreochromis niloticus*	Scale		Maze	100-1000	0.1-1	Flow control
163	*Oreochromis niloticus*	Scale		Maze	100-1000	0.1-1	Fluid retention
164	*Oreochromis niloticus*	Scale		Maze	100-1000	0.1-1	Structural support
165	*Pandarus bicolor*	Antenna	Adult	Parallel	10-100	1-10	Adhesion and friction
166	*Pandarus bicolor*	Antenna	Adult	Parallel	10-100	1-10	Chanelling
167	*Pandarus bicolor*	Antenna	Adult	Parallel	10-100	1-10	Structural support
168	*Pandarus bicolor*	Antenna	Adult	Parallel	10-100	1-10	
169	*Pandarus bicolor*	Maxilliped	Adult	Parallel	10-100	1-10	
170	*Pandarus bicolor*	Dorsal shield	Adult	Labyrinth/ Parallel	10-100	1-10	
171	*Pandarus bicolor*	Leg	Adult	Parallel	10-100	1-10	
172	*Pandarus bicolor*	Leg	Adult	Parallel	10-100	1-10	
173	*Pandarus bicolor*	Leg	Adult	Parallel	10-100	1-10	
174	*Pandarus bicolor*	Leg	Adult	Parallel	10-100	1-10	
175	*Pandarus bicolor*	Leg	Adult	Parallel	10-100	1-10	
176	*Pandarus bicolor*	Leg	Adult	Parallel	10-100	1-10	
177	*Paranaella luquei*	Skin	Adult	Perpendicular	0.1-1	1-10	Fluid exchange
178	*Paranaella luquei*	Skin	Adult	Perpendicular	0.1-1	1-10	Nutrient intake
179	*Patella vulgata*	Shell	Adult	Radial	10-100	1000-10000	Thermoregulation
180	*Periophthalmus magnuspinn*	Eye	Adult	Labyrinth	10-100	0.1-1	Fluid retention
181	*Periophthalmus magnuspinn*	Eye	Larvae	Labyrinth	10-100	0.1-1	Fluid retention
182	*Periophthalmus magnuspinn*	Eye	Adult	Labyrinth	10-100	0.1-1	Structural support
183	*Periophthalmus magnuspinn*	Eye	Larvae	Labyrinth	10-100	0.1-1	Structural support
184	*Pheopteryx conklini*	Egg	Adult	Maze	10-100	1-10	Adhesion and friction
185	*Pheopteryx conklini*	Egg	Adult	Maze	10-100	1-10	Chanelling
186	*Pheopteryx conklini*	Egg	Adult	Maze	10-100	1-10	Structural support
187	*Pimelodus maculatus*	Skin	Adult		100-1000		
188	*Poecilia reticulata (Peters)*	Scale	Adult	Maze	10-100	0.1-1	Chanelling
189	*Poecilia reticulata (Peters)*	Fin	Adult	Maze	10-100		Chanelling
190	*Poecilia reticulata (Peters)*	Scale	Adult	Maze	10-100	0.1-1	Friction and Wear reduction
191	*Poecilia reticulata (Peters)*	Fin	Adult	Maze	10-100		Friction and Wear reduction
192	*Pterophyllum scalare*	Scale		Maze	100-1000	0.1-1	Flow control
193	*Pterophyllum scalare*	Scale		Maze	100-1000	0.1-1	Fluid retention
194	*Pterophyllum scalare*	Scale		Maze	100-1000	0.1-1	Structural support

	N
162	Fishelson L. 1984 A comparative study of ridge-mazes on surface epithelial cell-membranes of fish scales (Pisces, Teleostei). Zoomorphology 104, 231–238. (
163	Fishelson L. 1984 A comparative study of ridge-mazes on surface epithelial cell-membranes of fish scales (Pisces, Teleostei). Zoomorphology 104, 231–238. (
164	Fishelson L. 1984 A comparative study of ridge-mazes on surface epithelial cell-membranes of fish scales (Pisces, Teleostei). Zoomorphology 104, 231–238. (
165	Ingram AL, Parker AR. 2006 The functional morphology and attachment mechanism of pandarid adhesion pads (Crustacea: Copepoda: Pandaridae). Zoologi
166	Ingram AL, Parker AR. 2006 The functional morphology and attachment mechanism of pandarid adhesion pads (Crustacea: Copepoda: Pandaridae). Zoologi
167	Ingram AL, Parker AR. 2006 The functional morphology and attachment mechanism of pandarid adhesion pads (Crustacea: Copepoda: Pandaridae). Zoologi
168	Ingram AL, Parker AR. 2006 The functional morphology and attachment mechanism of pandarid adhesion pads (Crustacea: Copepoda: Pandaridae). Zoologi
169	Ingram AL, Parker AR. 2006 The functional morphology and attachment mechanism of pandarid adhesion pads (Crustacea: Copepoda: Pandaridae). Zoologi
170	Ingram AL, Parker AR. 2006 The functional morphology and attachment mechanism of pandarid adhesion pads (Crustacea: Copepoda: Pandaridae). Zoologi
171	Ingram AL, Parker AR. 2006 The functional morphology and attachment mechanism of pandarid adhesion pads (Crustacea: Copepoda: Pandaridae). Zoologi
172	Ingram AL, Parker AR. 2006 The functional morphology and attachment mechanism of pandarid adhesion pads (Crustacea: Copepoda: Pandaridae). Zoologi
173	Ingram AL, Parker AR. 2006 The functional morphology and attachment mechanism of pandarid adhesion pads (Crustacea: Copepoda: Pandaridae). Zoologi
174	Ingram AL, Parker AR. 2006 The functional morphology and attachment mechanism of pandarid adhesion pads (Crustacea: Copepoda: Pandaridae). Zoologi
175	Ingram AL, Parker AR. 2006 The functional morphology and attachment mechanism of pandarid adhesion pads (Crustacea: Copepoda: Pandaridae). Zoologi
176	Ingram AL, Parker AR. 2006 The functional morphology and attachment mechanism of pandarid adhesion pads (Crustacea: Copepoda: Pandaridae). Zoologi
177	Cohen S, Kohn A, Baptista-Farias M. 2001 Scanning and transmission electron microscopy of the tegument of Paranaella luquei Kohn, Baptista-Farias & Coh
178	Cohen S, Kohn A, Baptista-Farias M. 2001 Scanning and transmission electron microscopy of the tegument of Paranaella luquei Kohn, Baptista-Farias & Coh
179	Harley CDG, Denny MW, Mach KJ, Miller LP. 2009 Thermal stress and morphological adaptations in limpets. Functional Ecology 23, 292–301. (doi:10.1111/j.1
180	Hu W, Zhang J, Kang B. 2016 Structure and function of corneal surface of mudskipper fishes. Fish Physiol Biochem 42, 1481–1489. (doi:10.1007/s10695-01
181	Hu W, Zhang J, Kang B. 2016 Structure and function of corneal surface of mudskipper fishes. Fish Physiol Biochem 42, 1481–1489. (doi:10.1007/s10695-01
182	Hu W, Zhang J, Kang B. 2016 Structure and function of corneal surface of mudskipper fishes. Fish Physiol Biochem 42, 1481–1489. (doi:10.1007/s10695-01
183	Hu W, Zhang J, Kang B. 2016 Structure and function of corneal surface of mudskipper fishes. Fish Physiol Biochem 42, 1481–1489. (doi:10.1007/s10695-01
184	Fishelson L, Gon O. 2008 Comparative oogenesis in cardinal fishes (Apogonidae, Perciformes), with special focus on the adaptive structures of the egg enve
185	Fishelson L, Gon O. 2008 Comparative oogenesis in cardinal fishes (Apogonidae, Perciformes), with special focus on the adaptive structures of the egg enve
186	Fishelson L, Gon O. 2008 Comparative oogenesis in cardinal fishes (Apogonidae, Perciformes), with special focus on the adaptive structures of the egg enve
187	Ferri S. 1982 Ultrastructural Observations on Nerve Fibres in a Freshwater Telecost Epidermis. Anatom Histol Embryol 11, 76–80. (doi:10.1111/j.1439-0264.1
188	Hunter CR, Nayudu PL. 1978 Surface folds in superficial epidermal cells of three species of teleost fish. J Fish Biology 12, 163–166. (doi:10.1111/j.1095-864
189	Hunter CR, Nayudu PL. 1978 Surface folds in superficial epidermal cells of three species of teleost fish. J Fish Biology 12, 163–166. (doi:10.1111/j.1095-864
190	Hunter CR, Nayudu PL. 1978 Surface folds in superficial epidermal cells of three species of teleost fish. J Fish Biology 12, 163–166. (doi:10.1111/j.1095-864
191	Hunter CR, Nayudu PL. 1978 Surface folds in superficial epidermal cells of three species of teleost fish. J Fish Biology 12, 163–166. (doi:10.1111/j.1095-864
192	Fishelson L. 1984 A comparative study of ridge-mazes on surface epithelial cell-membranes of fish scales (Pisces, Teleostei). Zoomorphology 104, 231–238. (
193	Fishelson L. 1984 A comparative study of ridge-mazes on surface epithelial cell-membranes of fish scales (Pisces, Teleostei). Zoomorphology 104, 231–238. (
194	Fishelson L. 1984 A comparative study of ridge-mazes on surface epithelial cell-membranes of fish scales (Pisces, Teleostei). Zoomorphology 104, 231–238. (

	A	B	C	D	E	F
195	Aquatic	Animalia	Chordata	Reptilia	Pterosauria	
196	Aquatic	Animalia	Chordata	Chondrichthyes	Elasmobranchii	Ptychodontidae
197	Aquatic	Animalia	Chordata	Actinopterygii	Perciformes	Apogonidae
198	Aquatic	Animalia	Chordata	Actinopterygii	Perciformes	Apogonidae
199	Aquatic	Animalia	Chordata	Actinopterygii	Perciformes	Apogonidae
200	Aquatic	Animalia	Chordata	Chondrichthyes	Rhinopristiformes	Rhinidae
201	Aquatic	Animalia	Chordata	Actinopterygii	Salmoniformes	Salmonidae
202	Aquatic	Animalia	Chordata	Actinopterygii	Salmoniformes	Salmonidae
203	Aquatic	Animalia	Chordata	Actinopterygii	Salmoniformes	Salmonidae
204	Aquatic	Animalia	Chordata	Actinopterygii	Salmoniformes	Salmonidae
205	Aquatic	Animalia	Chordata	Actinopterygii	Salmoniformes	Salmonidae
206	Aquatic	Animalia	Chordata	Actinopterygii	Cichliformes	Cichlidae
207	Aquatic	Animalia	Chordata	Actinopterygii	Cichliformes	Cichlidae
208	Aquatic	Animalia	Chordata	Actinopterygii	Cichliformes	Cichlidae
209	Aquatic	Animalia	Chordata	Actinopterygii	Cichliformes	Cichlidae
210	Aquatic	Animalia	Chordata	Actinopterygii	Cichliformes	Cichlidae
211	Aquatic	Animalia	Chordata	Actinopterygii	Cichliformes	Cichlidae
212	Aquatic	Animalia	Chordata	Actinopterygii	Cichliformes	Cichlidae
213	Aquatic	Animalia	Chordata	Actinopterygii	Cichliformes	Cichlidae
214	Aquatic	Animalia	Chordata	Actinopterygii	Cichliformes	Cichlidae
215	Aquatic	Animalia	Chordata	Actinopterygii	Perciformes	Cichlidae
216	Aquatic	Animalia	Chordata	Actinopterygii	Perciformes	Cichlidae
217	Aquatic	Animalia	Chordata	Actinopterygii	Perciformes	Cichlidae
218	Aquatic	Chromista	Ciliophora	Oligohymenophorea	Sessilida	Scyphidiidae
219	Aquatic	Animalia	Chordata	Actinopterygii	Scorpaeniformes	Scorpaenidae
220	Aquatic	Animalia	Chordata	Actinopterygii	Scorpaeniformes	Scorpaenidae
221	Aquatic	Animalia	Chordata	Actinopterygii	Scorpaeniformes	Scorpaenidae
222	Aquatic	Animalia	Chordata	Actinopterygii	Scorpaeniformes	Scorpaenidae
223	Aquatic	Animalia	Chordata	Actinopterygii	Scorpaeniformes	Scorpaenidae
224	Aquatic	Animalia	Chordata	Actinopterygii	Scorpaeniformes	Scorpaenidae
225	Aquatic	Animalia	Chordata	Actinopterygii	Scorpaeniformes	Scorpaenidae
226	Aquatic	Animalia	Chordata	Actinopterygii	Scorpaeniformes	Scorpaenidae
227	Aquatic	Animalia	Chordata	Actinopterygii	Scorpaeniformes	Scorpaenidae

	G	H	I	J	K	L	M
195	*pterosaurs*	Tooth	Adult	Parallel			Feeding
196	*Ptychodus sp.*	Tooth		Parallel/transverse	1000-10000	100-1000	Feeding
197	*Rhabdamia cypselura*	Egg	Adult	Maze	10-100	10-100	Adhesion and friction
198	*Rhabdamia cypselura*	Egg	Adult	Maze	10-100	10-100	Chanelling
199	*Rhabdamia cypselura*	Egg	Adult	Maze	10-100	10-100	Structural support
200	*Rhina ancylostoma*	Tooth	Adult	Parallel/transverse	1000-10000	100-1000	Feeding
201	*Salmo gairdneri*	Gill		Labyrinth	100-1000	0.1-1	Fluid exchange
202	*Salmo gairdneri*	Gill		Labyrinth	100-1000	0.1-1	Fluid retention
203	*Salmo gairdneri*	Gill		Labyrinth	100-1000	0.1-1	Structural support
204	*Salmo gairdneri*	Esophagus	Adult	Labyrinth	100-1000	0.1-1	Chanelling
205	*Salmo gairdneri*	Esophagus	Adult	Labyrinth	100-1000	0.1-1	Fluid retention
206	*Sarotherodon (Tilapia) galila*	Gill	Adult	Maze	100-1000	0.1-1	Chanelling
207	*Sarotherodon (Tilapia) galila*	Gill	Adult	Maze	100-1000	0.1-1	Osmoregulation
208	*Sarotherodon (Tilapia) galila*	Gill	Adult	Maze	100-1000	0.1-1	Structural support
209	*Sarotherodon (Tilapia) moss*	Gill	Adult	Maze	100-1000	0.1-1	Chanelling
210	*Sarotherodon (Tilapia) moss*	Gill	Adult	Maze	100-1000	0.1-1	Osmoregulation
211	*Sarotherodon (Tilapia) moss*	Gill	Adult	Maze	100-1000	0.1-1	Structural support
212	*Sarotherodon (Tilapia) niloti*	Gill	Adult	Maze	100-1000	0.1-1	Chanelling
213	*Sarotherodon (Tilapia) niloti*	Gill	Adult	Maze	100-1000	0.1-1	Osmoregulation
214	*Sarotherodon (Tilapia) niloti*	Gill	Adult	Maze	100-1000	0.1-1	Structural support
215	*Sarotherodon galilaeus*	Scale		Maze	100-1000	0.1-1	Flow control
216	*Sarotherodon galilaeus*	Scale		Maze	100-1000	0.1-1	Fluid retention
217	*Sarotherodon galilaeus*	Scale		Maze	100-1000	0.1-1	Structural support
218	*Scyphidia physarum*	Skin	Adult	Perpendicular	0.01-0.1	0.1-1	
219	*Sebastes carnatus*	Scale		Maze	100-1000	0.1-1	Flow control
220	*Sebastes carnatus*	Scale		Maze	100-1000	0.1-1	Fluid retention
221	*Sebastes carnatus*	Scale		Maze	100-1000	0.1-1	Structural support
222	*Sebastes chlorostictus*	Scale		Maze	100-1000	0.1-1	Flow control
223	*Sebastes chlorostictus*	Scale		Maze	100-1000	0.1-1	Flow control
224	*Sebastes chlorostictus*	Scale		Maze	100-1000	0.1-1	Fluid retention
225	*Sebastes chlorostictus*	Scale		Maze	100-1000	0.1-1	Fluid retention
226	*Sebastes chlorostictus*	Scale		Maze	100-1000	0.1-1	Structural support
227	*Sebastes chlorostictus*	Scale		Maze	100-1000	0.1-1	Structural support

	N
195	McCurry MR, Evans AR, Fitzgerald EMG, McHenry CR, Bevitt J, Pyenson ND. 2019 The repeated evolution of dental apicobasal ridges in aquatic-feeding mar
196	
197	Fishelson L, Gon O. 2008 Comparative oogenesis in cardinal fishes (Apogonidae, Perciformes), with special focus on the adaptive structures of the egg enve
198	Fishelson L, Gon O. 2008 Comparative oogenesis in cardinal fishes (Apogonidae, Perciformes), with special focus on the adaptive structures of the egg enve
199	Fishelson L, Gon O. 2008 Comparative oogenesis in cardinal fishes (Apogonidae, Perciformes), with special focus on the adaptive structures of the egg enve
200	
201	Olson KR, Fromm PO. 1973 A scanning electron microscopic study of secondary lamellae and chloride cells of rainbow trout (Salmo gairdneri). Z.Zellforsch 14
202	Olson KR, Fromm PO. 1973 A scanning electron microscopic study of secondary lamellae and chloride cells of rainbow trout (Salmo gairdneri). Z.Zellforsch 14
203	Olson KR, Fromm PO. 1973 A scanning electron microscopic study of secondary lamellae and chloride cells of rainbow trout (Salmo gairdneri). Z.Zellforsch 14
204	Sperry DG, Wassersug RJ. 1976 A proposed function for microridges on epithelial cells. Anat. Rec. 185, 253–257. (doi:10.1002/ar.1091850212)
205	Sperry DG, Wassersug RJ. 1976 A proposed function for microridges on epithelial cells. Anat. Rec. 185, 253–257. (doi:10.1002/ar.1091850212)
206	Fishelson L. 1980 Scanning and transmission electron microscopy of the squamose gill-filament epithelium from fresh- and seawater adaptedTilapia. Environ
207	Fishelson L. 1980 Scanning and transmission electron microscopy of the squamose gill-filament epithelium from fresh- and seawater adaptedTilapia. Environ
208	Fishelson L. 1980 Scanning and transmission electron microscopy of the squamose gill-filament epithelium from fresh- and seawater adaptedTilapia. Environ
209	Fishelson L. 1980 Scanning and transmission electron microscopy of the squamose gill-filament epithelium from fresh- and seawater adaptedTilapia. Environ
210	Fishelson L. 1980 Scanning and transmission electron microscopy of the squamose gill-filament epithelium from fresh- and seawater adaptedTilapia. Environ
211	Fishelson L. 1980 Scanning and transmission electron microscopy of the squamose gill-filament epithelium from fresh- and seawater adaptedTilapia. Environ
212	Fishelson L. 1980 Scanning and transmission electron microscopy of the squamose gill-filament epithelium from fresh- and seawater adaptedTilapia. Environ
213	Fishelson L. 1980 Scanning and transmission electron microscopy of the squamose gill-filament epithelium from fresh- and seawater adaptedTilapia. Environ
214	Fishelson L. 1980 Scanning and transmission electron microscopy of the squamose gill-filament epithelium from fresh- and seawater adaptedTilapia. Environ
215	Fishelson L. 1984 A comparative study of ridge-mazes on surface epithelial cell-membranes of fish scales (Pisces, Teleostei). Zoomorphology 104, 231–238.
216	Fishelson L. 1984 A comparative study of ridge-mazes on surface epithelial cell-membranes of fish scales (Pisces, Teleostei). Zoomorphology 104, 231–238.
217	Fishelson L. 1984 A comparative study of ridge-mazes on surface epithelial cell-membranes of fish scales (Pisces, Teleostei). Zoomorphology 104, 231–238.
218	Warren A. 1990 A Scanning Electron Microscopic Study of the Morphology of Scyphidia physarum Lachmann, 1856 (Ciliophora: Peritrichida), Scanning Micros
219	Fishelson L. 1984 A comparative study of ridge-mazes on surface epithelial cell-membranes of fish scales (Pisces, Teleostei). Zoomorphology 104, 231–238.
220	Fishelson L. 1984 A comparative study of ridge-mazes on surface epithelial cell-membranes of fish scales (Pisces, Teleostei). Zoomorphology 104, 231–238.
221	Fishelson L. 1984 A comparative study of ridge-mazes on surface epithelial cell-membranes of fish scales (Pisces, Teleostei). Zoomorphology 104, 231–238.
222	Fishelson L. 1984 A comparative study of ridge-mazes on surface epithelial cell-membranes of fish scales (Pisces, Teleostei). Zoomorphology 104, 231–238.
223	Fishelson L. 1984 A comparative study of ridge-mazes on surface epithelial cell-membranes of fish scales (Pisces, Teleostei). Zoomorphology 104, 231–238.
224	Fishelson L. 1984 A comparative study of ridge-mazes on surface epithelial cell-membranes of fish scales (Pisces, Teleostei). Zoomorphology 104, 231–238.
225	Warren A. 1990 A Scanning Electron Microscopic Study of the Morphology of Scyphidia physarum Lachmann, 1856 (Ciliophora: Peritrichida), Scanning Micros
226	Fishelson L. 1984 A comparative study of ridge-mazes on surface epithelial cell-membranes of fish scales (Pisces, Teleostei). Zoomorphology 104, 231–238.
227	Fishelson L. 1984 A comparative study of ridge-mazes on surface epithelial cell-membranes of fish scales (Pisces, Teleostei). Zoomorphology 104, 231–238.

	A	B	C	D	E	F
228	Aquatic	Animalia	Chordata	Actinopterygii	Scorpaeniformes	Scorpaenidae
229	Aquatic	Animalia	Chordata	Actinopterygii	Scorpaeniformes	Scorpaenidae
230	Aquatic	Animalia	Chordata	Actinopterygii	Scorpaeniformes	Scorpaenidae
231	Aquatic	Animalia	Chordata	Actinopterygii	Scorpaeniformes	Scorpaenidae
232	Aquatic	Animalia	Chordata	Actinopterygii	Scorpaeniformes	Scorpaenidae
233	Aquatic	Animalia	Chordata	Actinopterygii	Scorpaeniformes	Scorpaenidae
234	Aquatic	Animalia	Chordata	Actinopterygii	Scorpaeniformes	Scorpaenidae
235	Aquatic	Animalia	Chordata	Actinopterygii	Scorpaeniformes	Scorpaenidae
236	Aquatic	Animalia	Chordata	Actinopterygii	Scorpaeniformes	Scorpaenidae
237	Aquatic	Animalia	Chordata	Actinopterygii	Scorpaeniformes	Scorpaenidae
238	Aquatic	Animalia	Chordata	Actinopterygii	Scorpaeniformes	Scorpaenidae
239	Aquatic	Animalia	Chordata	Actinopterygii	Scorpaeniformes	Scorpaenidae
240	Aquatic	Animalia	Chordata	Actinopterygii	Scorpaeniformes	Scorpaenidae
241	Aquatic	Animalia	Chordata	Actinopterygii	Scorpaeniformes	Scorpaenidae
242	Aquatic	Animalia	Chordata	Actinopterygii	Scorpaeniformes	Scorpaenidae
243	Aquatic	Animalia	Chordata	Actinopterygii	Scorpaeniformes	Scorpaenidae
244	Aquatic	Animalia	Chordata	Actinopterygii	Scorpaeniformes	Scorpaenidae
245	Aquatic	Animalia	Chordata	Actinopterygii	Scorpaeniformes	Scorpaenidae
246	Aquatic	Animalia	Chordata	Actinopterygii	Scorpaeniformes	Scorpaenidae
247	Aquatic	Animalia	Chordata	Actinopterygii	Scorpaeniformes	Scorpaenidae
248	Aquatic	Animalia	Chordata	Actinopterygii	Scorpaeniformes	Scorpaenidae
249	Aquatic	Animalia	Chordata	Actinopterygii	Scorpaeniformes	Scorpaenidae
250	Aquatic	Animalia	Chordata	Actinopterygii	Scorpaeniformes	Scorpaenidae
251	Aquatic	Animalia	Chordata	Actinopterygii	Scorpaeniformes	Scorpaenidae
252	Aquatic	Animalia	Chordata	Actinopterygii	Scorpaeniformes	Scorpaenidae
253	Aquatic	Animalia	Chordata	Actinopterygii	Scorpaeniformes	Scorpaenidae
254	Aquatic	Animalia	Chordata	Actinopterygii	Scorpaeniformes	Scorpaenidae
255	Aquatic	Animalia	Mollusca	Cephalopoda	Sepiida	Sepiidae
256	Aquatic	Animalia	Mollusca	Cephalopoda	Sepiida	Sepiidae

	G	H	I	J	K	L	M
228	*Sebastes elongatus*	Scale		Maze	100-1000	0.1-1	Flow control
229	*Sebastes elongatus*	Scale		Maze	100-1000	0.1-1	Fluid retention
230	*Sebastes elongatus*	Scale		Maze	100-1000	0.1-1	Structural support
231	*Sebastes eos*	Scale		Maze	100-1000	0.1-1	Flow control
232	*Sebastes eos*	Scale		Maze	100-1000	0.1-1	Fluid retention
233	*Sebastes eos*	Scale		Maze	100-1000	0.1-1	Structural support
234	*Sebastes hopkinsi*	Scale		Maze	100-1000	0.1-1	Flow control
235	*Sebastes hopkinsi*	Scale		Maze	100-1000	0.1-1	Fluid retention
236	*Sebastes hopkinsi*	Scale		Maze	100-1000	0.1-1	Structural support
237	*Sebastes miniatus*	Scale		Maze	100-1000	0.1-1	Flow control
238	*Sebastes miniatus*	Scale		Maze	100-1000	0.1-1	Fluid retention
239	*Sebastes miniatus*	Scale		Maze	100-1000	0.1-1	Structural support
240	*Sebastes paucispinis*	Scale		Maze	100-1000	0.1-1	Flow control
241	*Sebastes paucispinis*	Scale		Maze	100-1000	0.1-1	Fluid retention
242	*Sebastes paucispinis*	Scale		Maze	100-1000	0.1-1	Structural support
243	*Sebastes rosaceus*	Scale		Maze	100-1000	0.1-1	Flow control
244	*Sebastes rosaceus*	Scale		Maze	100-1000	0.1-1	Fluid retention
245	*Sebastes rosaceus*	Scale		Maze	100-1000	0.1-1	Structural support
246	*Sebastes rubrivinetus*	Scale		Maze	100-1000	0.1-1	Flow control
247	*Sebastes rubrivinetus*	Scale		Maze	100-1000	0.1-1	Fluid retention
248	*Sebastes rubrivinetus*	Scale		Maze	100-1000	0.1-1	Structural support
249	*Sebastes serranoides*	Scale		Maze	100-1000	0.1-1	Flow control
250	*Sebastes serranoides*	Scale		Maze	100-1000	0.1-1	Fluid retention
251	*Sebastes serranoides*	Scale		Maze	100-1000	0.1-1	Structural support
252	*Sebastes serriceps*	Scale		Maze	100-1000	0.1-1	Flow control
253	*Sebastes serriceps*	Scale		Maze	100-1000	0.1-1	Fluid retention
254	*Sebastes serriceps*	Scale		Maze	100-1000	0.1-1	Structural support
255	*Sepia officinalis*	Gill		Perpendicular Labyrinth at junctions	100-1000	10-100	Contractile activity
256	*Sepia officinalis*	Gill		Perpendicular Labyrinth at junctions	100-1000	10-100	Fluid exchange

	N
228	Fishelson L. 1984 A comparative study of ridge-mazes on surface epithelial cell-membranes of fish scales (Pisces, Teleostei). Zoomorphology 104, 231–238.
229	Fishelson L. 1984 A comparative study of ridge-mazes on surface epithelial cell-membranes of fish scales (Pisces, Teleostei). Zoomorphology 104, 231–238.
230	Fishelson L. 1984 A comparative study of ridge-mazes on surface epithelial cell-membranes of fish scales (Pisces, Teleostei). Zoomorphology 104, 231–238.
231	Fishelson L. 1984 A comparative study of ridge-mazes on surface epithelial cell-membranes of fish scales (Pisces, Teleostei). Zoomorphology 104, 231–238.
232	Fishelson L. 1984 A comparative study of ridge-mazes on surface epithelial cell-membranes of fish scales (Pisces, Teleostei). Zoomorphology 104, 231–238.
233	Fishelson L. 1984 A comparative study of ridge-mazes on surface epithelial cell-membranes of fish scales (Pisces, Teleostei). Zoomorphology 104, 231–238.
234	Fishelson L. 1984 A comparative study of ridge-mazes on surface epithelial cell-membranes of fish scales (Pisces, Teleostei). Zoomorphology 104, 231–238.
235	Fishelson L. 1984 A comparative study of ridge-mazes on surface epithelial cell-membranes of fish scales (Pisces, Teleostei). Zoomorphology 104, 231–238.
236	Fishelson L. 1984 A comparative study of ridge-mazes on surface epithelial cell-membranes of fish scales (Pisces, Teleostei). Zoomorphology 104, 231–238.
237	Fishelson L. 1984 A comparative study of ridge-mazes on surface epithelial cell-membranes of fish scales (Pisces, Teleostei). Zoomorphology 104, 231–238.
238	Fishelson L. 1984 A comparative study of ridge-mazes on surface epithelial cell-membranes of fish scales (Pisces, Teleostei). Zoomorphology 104, 231–238.
239	Fishelson L. 1984 A comparative study of ridge-mazes on surface epithelial cell-membranes of fish scales (Pisces, Teleostei). Zoomorphology 104, 231–238.
240	Fishelson L. 1984 A comparative study of ridge-mazes on surface epithelial cell-membranes of fish scales (Pisces, Teleostei). Zoomorphology 104, 231–238.
241	Fishelson L. 1984 A comparative study of ridge-mazes on surface epithelial cell-membranes of fish scales (Pisces, Teleostei). Zoomorphology 104, 231–238.
242	Fishelson L. 1984 A comparative study of ridge-mazes on surface epithelial cell-membranes of fish scales (Pisces, Teleostei). Zoomorphology 104, 231–238.
243	Fishelson L. 1984 A comparative study of ridge-mazes on surface epithelial cell-membranes of fish scales (Pisces, Teleostei). Zoomorphology 104, 231–238.
244	Fishelson L. 1984 A comparative study of ridge-mazes on surface epithelial cell-membranes of fish scales (Pisces, Teleostei). Zoomorphology 104, 231–238.
245	Fishelson L. 1984 A comparative study of ridge-mazes on surface epithelial cell-membranes of fish scales (Pisces, Teleostei). Zoomorphology 104, 231–238.
246	Fishelson L. 1984 A comparative study of ridge-mazes on surface epithelial cell-membranes of fish scales (Pisces, Teleostei). Zoomorphology 104, 231–238.
247	Fishelson L. 1984 A comparative study of ridge-mazes on surface epithelial cell-membranes of fish scales (Pisces, Teleostei). Zoomorphology 104, 231–238.
248	Fishelson L. 1984 A comparative study of ridge-mazes on surface epithelial cell-membranes of fish scales (Pisces, Teleostei). Zoomorphology 104, 231–238.
249	Fishelson L. 1984 A comparative study of ridge-mazes on surface epithelial cell-membranes of fish scales (Pisces, Teleostei). Zoomorphology 104, 231–238.
250	Fishelson L. 1984 A comparative study of ridge-mazes on surface epithelial cell-membranes of fish scales (Pisces, Teleostei). Zoomorphology 104, 231–238.
251	Fishelson L. 1984 A comparative study of ridge-mazes on surface epithelial cell-membranes of fish scales (Pisces, Teleostei). Zoomorphology 104, 231–238.
252	Fishelson L. 1984 A comparative study of ridge-mazes on surface epithelial cell-membranes of fish scales (Pisces, Teleostei). Zoomorphology 104, 231–238.
253	Fishelson L. 1984 A comparative study of ridge-mazes on surface epithelial cell-membranes of fish scales (Pisces, Teleostei). Zoomorphology 104, 231–238.
254	Fishelson L. 1984 A comparative study of ridge-mazes on surface epithelial cell-membranes of fish scales (Pisces, Teleostei). Zoomorphology 104, 231–238.
255	Pappasio G, Pisanti FA. 1984 Scanning Electron Microscopy of the Cephalopod Gill: A Vascular Cast Study. Journal of Electron Microscopy (doi:10.1093/oxf
256	Pappasio G, Pisanti FA. 1984 Scanning Electron Microscopy of the Cephalopod Gill: A Vascular Cast Study. Journal of Electron Microscopy (doi:10.1093/oxf

	A	B	C	D	E	F
257	Aquatic	Animalia	Chordata	Actinopterygii	Perciformes	Apogonidae
258	Aquatic	Animalia	Chordata	Actinopterygii	Perciformes	Apogonidae
259	Aquatic	Animalia	Chordata	Actinopterygii	Perciformes	Apogonidae
260	Aquatic	Animalia	Chordata	Actinopterygii	Perciformes	Apogonidae
261	Aquatic	Animalia	Chordata	Actinopterygii	Perciformes	Apogonidae
262	Aquatic	Animalia	Chordata	Actinopterygii	Perciformes	Apogonidae
263	Aquatic	Animalia	Chordata	Actinopterygii	Perciformes	Apogonidae
264	Aquatic	Animalia	Chordata	Actinopterygii	Perciformes	Apogonidae
265	Aquatic	Animalia	Chordata	Actinopterygii	Perciformes	Apogonidae
266	Aquatic	Animalia	Chordata	Actinopterygii	Perciformes	Apogonidae
267	Aquatic	Animalia	Chordata	Actinopterygii	Perciformes	Apogonidae
268	Aquatic	Animalia	Chordata	Actinopterygii	Kurtiformes	Apogonidae
269	Aquatic	Animalia	Chordata	Actinopterygii	Kurtiformes	Apogonidae
270	Aquatic	Animalia	Chordata	Actinopterygii	Kurtiformes	Apogonidae
271	Aquatic	Animalia	Chordata	Actinopterygii	Kurtiformes	Apogonidae
272	Aquatic	Animalia	Chordata	Actinopterygii	Kurtiformes	Apogonidae
273	Aquatic	Animalia	Chordata	Actinopterygii	Kurtiformes	Apogonidae
274	Aquatic	Animalia	Chordata	Actinopterygii	Perciformes	Apogonidae
275	Aquatic	Animalia	Chordata	Actinopterygii	Perciformes	Apogonidae
276	Aquatic	Animalia	Chordata	Actinopterygii	Perciformes	Apogonidae
277	Aquatic	Animalia	Chordata	Actinopterygii	Kurtiformes	Apogonidae
278	Aquatic	Animalia	Chordata	Actinopterygii	Kurtiformes	Apogonidae
279	Aquatic	Animalia	Chordata	Actinopterygii	Kurtiformes	Apogonidae
280	Aquatic	Animalia	Mollusca	Gastropoda		Siphonariidae
281	Aquatic	Plantae	Angiosperms	Eudicots	Myrtales	Lythraceae
282	Aquatic	Animalia	Chordata	Actinopterygii	Perciformes	Apogonidae
283	Aquatic	Animalia	Chordata	Actinopterygii	Perciformes	Apogonidae
284	Aquatic	Animalia	Chordata	Actinopterygii	Perciformes	Apogonidae
285	Aquatic	Animalia	Arthropoda	Crustacea	Isopoda	Sphaeromatidae
286	Aquatic	Animalia	Arthropoda	Crustacea	Isopoda	Sphaeromatidae
287	Aquatic	Animalia	Chordata	Actinopterygii	Perciformes	Apogonidae
288	Aquatic	Animalia	Chordata	Reptilia	Crocodilia	
289	Aquatic	Animalia	Chordata	Reptilia	Crocodilia	

	G	H	I	J	K	L	M
257	*Siphamia cephalotes*	Tongue	Adult	Maze	10-100	0.1-1	Prey collection
258	*Siphamia mossambica*	Egg	Adult	Maze	10-100	1-10	Adhesion and friction
259	*Siphamia mossambica*	Egg	Adult	Maze	10-100	1-10	Chanelling
260	*Siphamia mossambica*	Egg	Adult	Maze	10-100	1-10	Structural support
261	*Siphamia permutata*	Egg	Adult	Maze		1-10	Adhesion and friction
262	*Siphamia permutata*	Egg	Adult	Maze		1-10	Chanelling
263	*Siphamia permutata*	Tongue	Adult	Maze			Prey collection
264	*Siphamia permutata*	Egg	Adult	Maze		1-10	Structural support
265	*Siphamia permutata*	Tongue	Adult	Maze		0.1-1	
266	*Siphamia roseigaster*	Egg	Adult	Maze	10-100		Adhesion and friction
267	*Siphamia roseigaster*	Egg	Adult	Maze	10-100		Structural support
268	*Siphamia sp.1*	Egg	Adult	Maze	10-100	1-10	Adhesion and friction
269	*Siphamia sp.1*	Egg	Adult	Maze	10-100	1-10	Chanelling
270	*Siphamia sp.1*	Egg	Adult	Maze	10-100	1-10	Structural support
271	*Siphamia sp.2*	Egg	Adult	Maze		0.1-1	Adhesion and friction
272	*Siphamia sp.2*	Egg	Adult	Maze		0.1-1	Chanelling
273	*Siphamia sp.2*	Egg	Adult	Maze		0.1-1	Structural support
274	*Siphamia versicolor*	Egg	Adult	Maze		1-10	Adhesion and friction
275	*Siphamia versicolor*	Egg	Adult	Maze		1-10	Chanelling
276	*Siphamia versicolor*	Egg	Adult	Maze		1-10	Structural support
277	*Siphamia versicolor*	Egg	Adult	Maze	10-100	1-10	Adhesion and friction
278	*Siphamia versicolor*	Egg	Adult	Maze	10-100	1-10	Chanelling
279	*Siphamia versicolor*	Egg	Adult	Maze	10-100	1-10	Structural support
280	*Siphonaria gigas*	Shell	Adult	Radial	10-100	1000-10000	Thermoregulation
281	*Sonneratia apetala*	Leaf	Adult	Labyrinth	10000-10000(	1-10	Biofouling
282	*Sphaeramia orbicularis*	Egg	Adult	Maze	10-100		Adhesion and friction
283	*Sphaeramia orbicularis*	Egg	Adult	Maze	10-100		Chanelling
284	*Sphaeramia orbicularis*	Egg	Adult	Maze	10-100		Structural support
285	*Sphaeroma serratum*	Leg	Adult	Parallel	10-100	0.1-1	Friction and Wear reduction
286	*Sphaeroma serratum*	Leg	Adult	Parallel	10-100	0.1-1	Particle removal
287	*Structural support, sound de*	Egg	Adult	Maze	10-100		Chanelling
288	*Thoracosaurus bahiensis*	Tooth		Radial		100-1000	
289	*Thoracosaurus neocesariens*	Tooth		Radial	1000-10000		

	N
257	Fishelson L, Gon O, Goren M, Ben-David-Zaslow R. 2005 The oral cavity and bioluminescent organs of the cardinal fish species Siphamia permutata and S. c
258	Fishelson L, Gon O. 2008 Comparative oogenesis in cardinal fishes (Apogonidae, Perciformes), with special focus on the adaptive structures of the egg enve
259	Fishelson L, Gon O. 2008 Comparative oogenesis in cardinal fishes (Apogonidae, Perciformes), with special focus on the adaptive structures of the egg enve
260	Fishelson L, Gon O. 2008 Comparative oogenesis in cardinal fishes (Apogonidae, Perciformes), with special focus on the adaptive structures of the egg enve
261	Fishelson L, Gon O. 2008 Comparative oogenesis in cardinal fishes (Apogonidae, Perciformes), with special focus on the adaptive structures of the egg enve
262	Fishelson L, Gon O. 2008 Comparative oogenesis in cardinal fishes (Apogonidae, Perciformes), with special focus on the adaptive structures of the egg enve
263	Fishelson L, Gon O, Goren M, Ben-David-Zaslow R. 2005 The oral cavity and bioluminescent organs of the cardinal fish species Siphamia permutata and S. c
264	Fishelson L, Gon O. 2008 Comparative oogenesis in cardinal fishes (Apogonidae, Perciformes), with special focus on the adaptive structures of the egg enve
265	Fishelson L, Delarea Y, Zverdling A. 2004 Taste bud form and distribution on lips and in the oropharyngeal cavity of cardinal fish species (Apogonidae, Teleo:
266	Fishelson L, Gon O. 2008 Comparative oogenesis in cardinal fishes (Apogonidae, Perciformes), with special focus on the adaptive structures of the egg enve
267	Fishelson L, Gon O. 2008 Comparative oogenesis in cardinal fishes (Apogonidae, Perciformes), with special focus on the adaptive structures of the egg enve
268	Fishelson L, Gon O. 2008 Comparative oogenesis in cardinal fishes (Apogonidae, Perciformes), with special focus on the adaptive structures of the egg enve
269	Fishelson L, Gon O. 2008 Comparative oogenesis in cardinal fishes (Apogonidae, Perciformes), with special focus on the adaptive structures of the egg enve
270	Fishelson L, Gon O. 2008 Comparative oogenesis in cardinal fishes (Apogonidae, Perciformes), with special focus on the adaptive structures of the egg enve
271	Fishelson L, Gon O. 2008 Comparative oogenesis in cardinal fishes (Apogonidae, Perciformes), with special focus on the adaptive structures of the egg enve
272	Fishelson L, Gon O. 2008 Comparative oogenesis in cardinal fishes (Apogonidae, Perciformes), with special focus on the adaptive structures of the egg enve
273	Fishelson L, Gon O. 2008 Comparative oogenesis in cardinal fishes (Apogonidae, Perciformes), with special focus on the adaptive structures of the egg enve
274	Fishelson L, Gon O. 2008 Comparative oogenesis in cardinal fishes (Apogonidae, Perciformes), with special focus on the adaptive structures of the egg enve
275	Fishelson L, Gon O. 2008 Comparative oogenesis in cardinal fishes (Apogonidae, Perciformes), with special focus on the adaptive structures of the egg enve
276	Fishelson L, Gon O. 2008 Comparative oogenesis in cardinal fishes (Apogonidae, Perciformes), with special focus on the adaptive structures of the egg enve
277	Fishelson L, Gon O. 2008 Comparative oogenesis in cardinal fishes (Apogonidae, Perciformes), with special focus on the adaptive structures of the egg enve
278	Fishelson L, Gon O. 2008 Comparative oogenesis in cardinal fishes (Apogonidae, Perciformes), with special focus on the adaptive structures of the egg enve
279	Fishelson L, Gon O. 2008 Comparative oogenesis in cardinal fishes (Apogonidae, Perciformes), with special focus on the adaptive structures of the egg enve
280	Harley CDG, Denny MW, Mach KJ, Miller LP. 2009 Thermal stress and morphological adaptations in limpets. Functional Ecology 23, 292–301. (doi:10.1111/j.1
281	Feng D, Wang W, Wang X, Qiu Y, Ke C. 2016 Low barnacle fouling on leaves of the mangrove plant Sonneratia apetala and possible anti-barnacle defense
282	Fishelson L, Gon O. 2008 Comparative oogenesis in cardinal fishes (Apogonidae, Perciformes), with special focus on the adaptive structures of the egg enve
283	Fishelson L, Gon O. 2008 Comparative oogenesis in cardinal fishes (Apogonidae, Perciformes), with special focus on the adaptive structures of the egg enve
284	Fishelson L, Gon O. 2008 Comparative oogenesis in cardinal fishes (Apogonidae, Perciformes), with special focus on the adaptive structures of the egg enve
285	Vittori M. 2021 Structure of a hinge joint with textured sliding surfaces in terrestrial isopods (Crustacea: Isopoda: Oniscidea). Zoological Lett 7, 7. (doi:10.1186
286	Vittori M. 2021 Structure of a hinge joint with textured sliding surfaces in terrestrial isopods (Crustacea: Isopoda: Oniscidea). Zoological Lett 7, 7. (doi:10.1186
287	Fishelson L, Gon O. 2008 Comparative oogenesis in cardinal fishes (Apogonidae, Perciformes), with special focus on the adaptive structures of the egg enve
288	G. Souza R, Riff D, W. A. Kellner A. 2015 Taxonomic revision of Thoracosaurus bahiensis Marsh, 1869, a supposed Gavialoidea (Reptilia, Crocodylia) from Cr
289	G. Souza R, Riff D, W. A. Kellner A. 2015 Taxonomic revision of Thoracosaurus bahiensis Marsh, 1869, a supposed Gavialoidea (Reptilia, Crocodylia) from Cr

	A	B	C	D	E	F
290	Aquatic	Animalia	Chordata	Actinopterygii	Cichliformes	Cichlidae
291	Aquatic	Animalia	Chordata	Actinopterygii	Perciformes	Cichlidae
292	Aquatic	Animalia	Chordata	Actinopterygii	Perciformes	Cichlidae
293	Aquatic	Animalia	Chordata	Actinopterygii	Perciformes	Cichlidae
294	Aquatic	Animalia	Chordata	Actinopterygii	Perciformes	Cichlidae
295	Aquatic	Animalia	Chordata	Actinopterygii	Perciformes	Cichlidae
296	Aquatic	Animalia	Chordata	Actinopterygii	Perciformes	Cichlidae
297	Aquatic	Animalia	Chordata	Actinopterygii	Perciformes	Cichlidae
298	Aquatic	Animalia	Chordata	Actinopterygii	Perciformes	Cichlidae
299	Aquatic	Animalia	Chordata	Actinopterygii	Perciformes	Cichlidae
300	Aquatic	Animalia	Chordata	Actinopterygii	Perciformes	Cichlidae
301	Aquatic	Animalia	Chordata	Actinopterygii	Perciformes	Cichlidae
302	Aquatic	Animalia	Chordata	Actinopterygii	Perciformes	Cichlidae
303	Aquatic	Animalia	Chordata	Mammalia	Artiodactyla	Delphinidae
304	Aquatic	Animalia	Chordata	Mammalia	Artiodactyla	Delphinidae
305	Aquatic	Animalia	Chordata	Mammalia	Artiodactyla	Delphinidae
306	Aquatic - extinct	Animalia	Chordata	Sauropsida	Theropoda	Spinosauridae
307	Aquatic - extinct	Animalia	Porifera	Demospongiae	Lithistida	Anthaspidellidae
308	Aquatic - extinct	Animalia	Porifera	Demospongiae	Lithistida	Anthaspidellidae
309	Aquatic - extinct	Animalia	Porifera	Demospongiae	Lithistida	Anthaspidellidae
310	Aquatic - extinct	Animalia	Porifera	Demospongiae	Lithistida	Anthaspidellidae
311	Aquatic - extinct	Animalia	Porifera	Demospongiae	Lithistida	Anthaspidellidae
312	Aquatic - extinct	Animalia	Porifera	Demospongiae	Lithistida	Anthaspidellidae
313	Aquatic - extinct	Animalia	Porifera	Demospongiae	Lithistida	Anthaspidellidae
314	Aquatic - extinct	Animalia	Porifera	Demospongiae	Lithistida	Anthaspidellidae
315	Aquatic - extinct	Animalia	Porifera	Demospongiae	Lithistida	Anthaspidellidae
316	Aquatic - extinct	Animalia	Porifera	Demospongiae	Lithistida	Anthaspidellidae
317	Aquatic - extinct	Animalia	Porifera	Demospongiae	Lithistida	Anthaspidellidae
318	Aquatic - extinct	Animalia	Porifera	Demospongiae	Lithistida	Anthaspidellidae
319	Aquatic - extinct	Animalia	Porifera	Demospongiae	Lithistida	Anthaspidellidae
320	Aquatic - extinct	Animalia	Porifera	Demospongiae	Lithistida	Anthaspidellidae
321	Aquatic - extinct	Animalia	Porifera	Demospongiae	Lithistida	Anthaspidellidae
322	Aquatic - extinct	Animalia	Porifera	Demospongiae	Lithistida	Anthaspidellidae
323	Aquatic - extinct	Animalia	Porifera	Demospongiae	Lithistida	Anthaspidellidae

	G	H	I	J	K	L	M
290	*Tilapia mossambica (Peters)*	Scale	Adult	Maze	100-1000	0.1-1	Structural support
291	*Tilapia zillii*	Gill	Adult	Maze	100-1000	0.1-1	Chanelling
292	*Tilapia zillii*	Scale		Maze	100-1000	0.1-1	Flow control
293	*Tilapia zillii*	Scale		Maze	100-1000	0.1-1	Flow control
294	*Tilapia zillii*	Scale		Maze	100-1000	0.1-1	Fluid retention
295	*Tilapia zillii*	Scale		Maze	100-1000	0.1-1	Fluid retention
296	*Tilapia zillii*	Gill	Adult	Maze	100-1000	0.1-1	Osmoregulation
297	*Tilapia zillii*	Gill	Adult	Maze	100-1000	0.1-1	Structural support
298	*Tilapia zillii*	Scale		Maze	100-1000	0.1-1	Structural support
299	*Tilapia zillii*	Scale		Maze	100-1000	0.1-1	Structural support
300	*Tristramella sacra*	Gill	Adult	Maze	100-1000	0.1-1	Chanelling
301	*Tristramella sacra*	Gill	Adult	Maze	100-1000	0.1-1	Osmoregulation
302	*Tristramella sacra*	Gill	Adult	Maze	100-1000	0.1-1	Structural support
303	*Tursiops truncatus*	Skin	Adult	Parallel	1000-10000	1-10	Flow control
304	*Tursiops truncatus*	Skin	Adult	Parallel	1000-10000		Flow control
305	*Tursiops truncatus*	Skin	Adult	Parallel	1000-10000	1-10	Sensing
306	*Angaturama limai*	Tooth		Radial	1000-10000		
307	*Anthaspidella annulata*	Skin				1000-10000	Flow control
308	*Anthaspidella annulata*	Skin				1000-10000	Nutrient intake
309	*Archaeoscyphia bassleri*	Skin				1000-10000	Flow control
310	*Archaeoscyphia bassleri*	Skin				1000-10000	Nutrient intake
311	*Archaeoscyphia boltoni*					10000-100000	Flow control
312	*Archaeoscyphia boltoni*					10000-100000	Nutrient intake
313	*Archaeoscyphia eganensis*	Skin				1000-10000	Flow control
314	*Archaeoscyphia eganensis*	Skin				1000-10000	Nutrient intake
315	*Archaeoscyphia mazourkensis*					10000-100000	Flow control
316	*Archaeoscyphia mazourkensis*					10000-100000	Nutrient intake
317	*Archaeoscyphia minganensi*	Skin		Parallel		10000-100000	Flow control
318	*Archaeoscyphia minganensi*	Skin		Parallel		10000-100000	Nutrient intake
319	*Archaeoscyphia nana*	Skin				1000-10000	Flow control
320	*Archaeoscyphia nana*	Skin				1000-10000	Nutrient intake
321	*Archaeoscyphia pannosa*	Skin				1000-10000	Flow control
322	*Archaeoscyphia pannosa*	Skin				1000-10000	Nutrient intake
323	*Archaeoscyphia pulchra*	Skin				10000-100000	Flow control

	N
290	Lanzing WJR, Higginbotham DR. 1974 Scanning microscopy of surface structures of Tilapia mossambica (Peters) scales. J Fish Biology 6, 307–310. (doi:10.1
291	Fishelson L. 1980 Scanning and transmission electron microscopy of the squamose gill-filament epithelium from fresh- and seawater adaptedTilapia. Environ
292	Fishelson L. 1984 A comparative study of ridge-mazes on surface epithelial cell-membranes of fish scales (Pisces, Teleostei). Zoomorphology 104, 231–238.
293	Fishelson L. 1984 A comparative study of ridge-mazes on surface epithelial cell-membranes of fish scales (Pisces, Teleostei). Zoomorphology 104, 231–238.
294	Fishelson L. 1984 A comparative study of ridge-mazes on surface epithelial cell-membranes of fish scales (Pisces, Teleostei). Zoomorphology 104, 231–238.
295	Fishelson L. 1984 A comparative study of ridge-mazes on surface epithelial cell-membranes of fish scales (Pisces, Teleostei). Zoomorphology 104, 231–238.
296	Fishelson L. 1980 Scanning and transmission electron microscopy of the squamose gill-filament epithelium from fresh- and seawater adaptedTilapia. Environ
297	Fishelson L. 1980 Scanning and transmission electron microscopy of the squamose gill-filament epithelium from fresh- and seawater adaptedTilapia. Environ
298	Fishelson L. 1984 A comparative study of ridge-mazes on surface epithelial cell-membranes of fish scales (Pisces, Teleostei). Zoomorphology 104, 231–238.
299	Fishelson L. 1984 A comparative study of ridge-mazes on surface epithelial cell-membranes of fish scales (Pisces, Teleostei). Zoomorphology 104, 231–238.
300	Fishelson L. 1980 Scanning and transmission electron microscopy of the squamose gill-filament epithelium from fresh- and seawater adaptedTilapia. Environ
301	Fishelson L. 1980 Scanning and transmission electron microscopy of the squamose gill-filament epithelium from fresh- and seawater adaptedTilapia. Environ
302	Fishelson L. 1980 Scanning and transmission electron microscopy of the squamose gill-filament epithelium from fresh- and seawater adaptedTilapia. Environ
303	Wainwright DK, Fish FE, Ingersoll S, Williams TM, St Leger J, Smits AJ, Lauder GV. 2019 How smooth is a dolphin? The ridged skin of odontocetes. Biol. Lett
304	Ridgway SH, Carder DA. 1993 Features of dolphin skin with potential hydrodynamic importance. IEEE Eng. Med. Biol. Mag. 12, 83–88. (doi:10.1109/51.2323
305	Wainwright DK, Fish FE, Ingersoll S, Williams TM, St Leger J, Smits AJ, Lauder GV. 2019 How smooth is a dolphin? The ridged skin of odontocetes. Biol. Lett
306	G. Souza R, Riff D, W. A. Kellner A. 2015 Taxonomic revision of Thoracosaurus bahiensis Marsh, 1869, a supposed Gavialoidea (Reptilia, Crocodylia) from Cr
307	Church SB. 2017 Efficient Ornamentation in Ordovician Anthaspidellid Sponges. PC (doi:10.17161/1808.24771)
308	Church SB. 2017 Efficient Ornamentation in Ordovician Anthaspidellid Sponges. PC (doi:10.17161/1808.24771)
309	Church SB. 2017 Efficient Ornamentation in Ordovician Anthaspidellid Sponges. PC (doi:10.17161/1808.24771)
310	Church SB. 2017 Efficient Ornamentation in Ordovician Anthaspidellid Sponges. PC (doi:10.17161/1808.24771)
311	Church SB. 2017 Efficient Ornamentation in Ordovician Anthaspidellid Sponges. PC (doi:10.17161/1808.24771)
312	Church SB. 2017 Efficient Ornamentation in Ordovician Anthaspidellid Sponges. PC (doi:10.17161/1808.24771)
313	Church SB. 2017 Efficient Ornamentation in Ordovician Anthaspidellid Sponges. PC (doi:10.17161/1808.24771)
314	Church SB. 2017 Efficient Ornamentation in Ordovician Anthaspidellid Sponges. PC (doi:10.17161/1808.24771)
315	Church SB. 2017 Efficient Ornamentation in Ordovician Anthaspidellid Sponges. PC (doi:10.17161/1808.24771)
316	Church SB. 2017 Efficient Ornamentation in Ordovician Anthaspidellid Sponges. PC (doi:10.17161/1808.24771)
317	Church SB. 2017 Efficient Ornamentation in Ordovician Anthaspidellid Sponges. PC (doi:10.17161/1808.24771)
318	Church SB. 2017 Efficient Ornamentation in Ordovician Anthaspidellid Sponges. PC (doi:10.17161/1808.24771)
319	Church SB. 2017 Efficient Ornamentation in Ordovician Anthaspidellid Sponges. PC (doi:10.17161/1808.24771)
320	Church SB. 2017 Efficient Ornamentation in Ordovician Anthaspidellid Sponges. PC (doi:10.17161/1808.24771)
321	Church SB. 2017 Efficient Ornamentation in Ordovician Anthaspidellid Sponges. PC (doi:10.17161/1808.24771)
322	Church SB. 2017 Efficient Ornamentation in Ordovician Anthaspidellid Sponges. PC (doi:10.17161/1808.24771)
323	Church SB. 2017 Efficient Ornamentation in Ordovician Anthaspidellid Sponges. PC (doi:10.17161/1808.24771)

	A	B	C	D	E	F
324	Aquatic - extinct	Animalia	Porifera	Demospongiae	Lithistida	Anthaspidellidae
325	Aquatic - extinct	Animalia	Porifera	Demospongiae	Lithistida	Anthaspidellidae
326	Aquatic - extinct	Animalia	Porifera	Demospongiae	Lithistida	Anthaspidellidae
327	Aquatic - extinct	Animalia	Porifera	Demospongiae	Lithistida	Anthaspidellidae
328	Aquatic - extinct	Animalia	Porifera	Demospongiae	Lithistida	Anthaspidellidae
329	Aquatic - extinct	Animalia	Chordata	Thelodonti	Thelodontiformes	Turiniidae
330	Aquatic - extinct	Animalia	Chordata	Thelodonti	Thelodontiformes	Barlowodidae
331	Aquatic - extinct	Animalia	Chordata	Thelodonti	Thelodontiformes	Barlowodidae
332	Aquatic - extinct	Animalia	Chordata	Thelodonti	Thelodontiformes	Boothialepididae
333	Aquatic - extinct	Animalia	Chordata	Thelodonti		Nikoliviidae
334	Aquatic - extinct	Animalia	Chordata	Acanthodii	Ischnacanthiformes	Ischnacanthidae
335	Aquatic - extinct	Animalia	Chordata	Sauropsida		Dyrosauridae
336	Aquatic - extinct	Animalia	Chordata	Reptilia	Ichthyosauria	
337	Aquatic - extinct	Animalia	Chordata	Sauropsida	Theropoda	Spinosauridae
338	Aquatic - extinct	Animalia	Porifera	Demospongiae	Orchocladina	Anthaspidellidae
339	Aquatic - extinct	Animalia	Porifera	Demospongiae	Orchocladina	Anthaspidellidae
340	Aquatic - extinct	Animalia	Chordata	Reptilia	Nothosauroidea	Nothosauridae
341	Aquatic - extinct	Animalia	Chordata	Osteichthyes incertae sedis	Lophosteiformes	Lophosteidae
342	Aquatic - extinct	Animalia	Chordata	Reptilia		Machimosauridae
343	Aquatic - extinct	Animalia	Chordata	Reptilia		Machimosauridae
344	Aquatic - extinct	Animalia	Chordata	Reptilia		Notosuchidae
345	Aquatic - extinct	Animalia	Arthropoda	Trilobita	Redlichiida	Laudoniidae
346	Aquatic - extinct	Animalia	Arthropoda	Trilobita	Redlichiida	Laudoniidae
347	Aquatic - extinct	Animalia	Porifera	Demospongiae	Lithistida	Anthaspidellidae
348	Aquatic - extinct	Animalia	Porifera	Demospongiae	Lithistida	Anthaspidellidae
349	Aquatic - extinct	Animalia	Porifera	Demospongiae	Lithistida	Anthaspidellidae
350	Aquatic - extinct	Animalia	Porifera	Demospongiae	Lithistida	Anthaspidellidae
351	Aquatic - extinct	Animalia	Arthropoda	Trilobita		
352	Aquatic - extinct	Animalia	Chordata	Reptilia	Nothosauroidea	Nothosauridae
353	Aquatic - extinct	Animalia	Arthropoda	Trilobita	Corynexochida	Styginidae
354	Aquatic - extinct	Animalia	Arthropoda	Trilobita	Corynexochida	Styginidae
355	Aquatic - extinct	Animalia	Arthropoda	Trilobita	Corynexochida	Styginidae
356	Aquatic - extinct	Animalia	Arthropoda	Trilobita	Corynexochida	Styginidae

	G	H	I	J	K	L	M
324	*Archaeoscyphia pulchra*	Skin				10000-100000	Nutrient intake
325	*Archaeoscyphia rossi*	Skin				1000-10000	Flow control
326	*Archaeoscyphia rossi*	Skin				1000-10000	Nutrient intake
327	*Archaeoscyphia undulata*	Skin				10000-100000	Flow control
328	*Archaeoscyphia undulata*	Skin				10000-100000	Nutrient intake
329	*Australolepis seddoni*	Denticle		Parallel	100-1000	1-10	Attachment
330	*Barlowodus excelsus*	Denticle		Parallel	100-1000	1-10	Attachment
331	*Barlowodus thorsteinssoni*	Denticle		Parallel	100-1000	1-10	Attachment
332	*Boothialepis thorsteinssoni*	Denticle		Parallel	100-1000	1-10	Attachment
333	*Chattertonodus cometoides*	Denticle		Parallel	100-1000	1-10	Attachment
334	*Gomphonchus sandelensis*	Denticle		Parallel	1000-10000	1-10	Attachment
335	*Hyposaurus derbianus*	Tooth		Radial		100-1000	
336	*Ichthyosaurus*	Tooth	Adult	Parallel	1000-10000	100-1000	Feeding
337	*Irritator challengeri*	Tooth		Radial	1000-10000	100-1000	
338	*Jianghania yichangensis*	Skin				10000-100000	Flow control
339	*Jianghania yichangensis*	Skin				10000-100000	Nutrient intake
340	*Lamprosauroides goepperti*	Tooth		Radial		10-100	
341	*Lophosteus sp.*	Denticle		Parallel		1-10	Attachment
342	*Machimosaurus buffetauti*	Tooth	Adult	Parallel	1000-10000	100-1000	Feeding
343	*Machimosaurus hugii*	Tooth	Adult	Parallel	1000-10000	100-1000	Feeding
344	*MARILIASUCHUS AMARALI*	Tooth	Adult	Parallel and labyrinth	1000-10000	100-1000	Feeding
345	*Mummaspis truncatooculatus*	Skin	Adult	Wavy	1-100	10-100	Adhesion and friction
346	*Mummaspis truncatooculatus*	Skin	Adult	Wavy	1-100	10-100	Structural support
347	*Nevadocoelia grandis*	Skin				10000-100000	Flow control
348	*Nevadocoelia grandis*	Skin				10000-100000	Nutrient intake
349	*Nevadocoelia wistae*	Skin				1000-10000	Flow control
350	*Nevadocoelia wistae*	Skin				1000-10000	Nutrient intake
351	*Nileus nileonis*	Skin	Adult	Wavy			
352	*Nothosaurus sp*	Tooth		Radial	1000-10000	10-100	
353	*Paralejurus aff. brongniarti*	Skin	Adult	Wavy			Particle removal
354	*Paralejurus bohemicus*	Skin	Adult	Wavy			Particle removal
355	*Paralejurus brongniarti*	Skin	Adult	Wavy			Particle removal
356	*Paralejurus brongniarti cf. me*	Skin	Adult	Wavy		10-100	Particle removal

	N
324	Church SB. 2017 Efficient Ornamentation in Ordovician Anthaspidellid Sponges. PC (doi:10.17161/1808.24771)
325	Church SB. 2017 Efficient Ornamentation in Ordovician Anthaspidellid Sponges. PC (doi:10.17161/1808.24771)
326	Church SB. 2017 Efficient Ornamentation in Ordovician Anthaspidellid Sponges. PC (doi:10.17161/1808.24771)
327	Church SB. 2017 Efficient Ornamentation in Ordovician Anthaspidellid Sponges. PC (doi:10.17161/1808.24771)
328	Church SB. 2017 Efficient Ornamentation in Ordovician Anthaspidellid Sponges. PC (doi:10.17161/1808.24771)
329	Märss T. 2006 Exoskeletal ultrasculpture of early vertebrates. Journal of Vertebrate Paleontology 26, 235–252. (doi:10.1671/0272-4634(2006)26[235:EUOE
330	Märss T. 2006 Exoskeletal ultrasculpture of early vertebrates. Journal of Vertebrate Paleontology 26, 235–252. (doi:10.1671/0272-4634(2006)26[235:EUOE
331	Märss T. 2006 Exoskeletal ultrasculpture of early vertebrates. Journal of Vertebrate Paleontology 26, 235–252. (doi:10.1671/0272-4634(2006)26[235:EUOE
332	Märss T. 2006 Exoskeletal ultrasculpture of early vertebrates. Journal of Vertebrate Paleontology 26, 235–252. (doi:10.1671/0272-4634(2006)26[235:EUOE
333	Märss T. 2006 Exoskeletal ultrasculpture of early vertebrates. Journal of Vertebrate Paleontology 26, 235–252. (doi:10.1671/0272-4634(2006)26[235:EUOE
334	Märss T. 2006 Exoskeletal ultrasculpture of early vertebrates. Journal of Vertebrate Paleontology 26, 235–252. (doi:10.1671/0272-4634(2006)26[235:EUOE
335	G. Souza R, Riff D, W. A. Kellner A. 2015 Taxonomic revision of Thoracosaurus bahiensis Marsh, 1869, a supposed Gavialoidea (Reptilia, Crocodylia) from Cr
336	McCurry MR, Evans AR, Fitzgerald EMG, McHenry CR, Bevitt J, Pyenson ND. 2019 The repeated evolution of dental apicobasal ridges in aquatic-feeding mar
337	G. Souza R, Riff D, W. A. Kellner A. 2015 Taxonomic revision of Thoracosaurus bahiensis Marsh, 1869, a supposed Gavialoidea (Reptilia, Crocodylia) from Cr
338	Church SB. 2017 Efficient Ornamentation in Ordovician Anthaspidellid Sponges. PC (doi:10.17161/1808.24771)
339	Church SB. 2017 Efficient Ornamentation in Ordovician Anthaspidellid Sponges. PC (doi:10.17161/1808.24771)
340	Spiekman SNF, Klein N. 2021 An enigmatic lower jaw from the Lower Muschelkalk (Anisian, Middle Triassic) of Winterswijk provides insights into dental configu
341	Märss T. 2006 Exoskeletal ultrasculpture of early vertebrates. Journal of Vertebrate Paleontology 26, 235–252. (doi:10.1671/0272-4634(2006)26[235:EUOE
342	Young MT, Steel L, Brusatte SL, Foffa D, Lepage Y. In press. Tooth serration morphologies in the genus Machimosaurus (Crocodylomorpha, Thalattosuchia)
343	Young MT, Steel L, Brusatte SL, Foffa D, Lepage Y. In press. Tooth serration morphologies in the genus Machimosaurus (Crocodylomorpha, Thalattosuchia)
344	Andrade, Marco & Bertini, Reinaldo. (2008). Morphology of the dental carinae in Mariliasuchus amarali (Crocodylomorpha, Notosuchia) and the pattern of toot
345	Ortega-Hernández J, Esteve J, Butterfield NJ. 2013 Humble origins for a successful strategy: complete enrolment in early Cambrian olenellid trilobites. Biol. Le
346	Ortega-Hernández J, Esteve J, Butterfield NJ. 2013 Humble origins for a successful strategy: complete enrolment in early Cambrian olenellid trilobites. Biol. Le
347	Church SB. 2017 Efficient Ornamentation in Ordovician Anthaspidellid Sponges. PC (doi:10.17161/1808.24771)
348	Church SB. 2017 Efficient Ornamentation in Ordovician Anthaspidellid Sponges. PC (doi:10.17161/1808.24771)
349	Church SB. 2017 Efficient Ornamentation in Ordovician Anthaspidellid Sponges. PC (doi:10.17161/1808.24771)
350	Church SB. 2017 Efficient Ornamentation in Ordovician Anthaspidellid Sponges. PC (doi:10.17161/1808.24771)
351	Nai-ren, H., 1984. The panderian organs of Nileus nileonis (Trilobita). Acta Palaeontologica Sinica.
352	Spiekman SNF, Klein N. 2021 An enigmatic lower jaw from the Lower Muschelkalk (Anisian, Middle Triassic) of Winterswijk provides insights into dental configu
353	Feist R, Lerosey-Aubril R, Johnson R. 2010 Coaptative devices, enrollment, and life habits in Paralejurus, a particular case in scutelluid trilobites. Palaeobio Pa
354	Feist R, Lerosey-Aubril R, Johnson R. 2010 Coaptative devices, enrollment, and life habits in Paralejurus, a particular case in scutelluid trilobites. Palaeobio Pa
355	Feist R, Lerosey-Aubril R, Johnson R. 2010 Coaptative devices, enrollment, and life habits in Paralejurus, a particular case in scutelluid trilobites. Palaeobio Pa
356	Feist R, Lerosey-Aubril R, Johnson R. 2010 Coaptative devices, enrollment, and life habits in Paralejurus, a particular case in scutelluid trilobites. Palaeobio Pa

	A	B	C	D	E	F
357	Aquatic - extinct	Animalia	Arthropoda	Trilobita	Corynexochida	Styginidae
358	Aquatic - extinct	Animalia	Arthropoda	Trilobita	Corynexochida	Styginidae
359	Aquatic - extinct	Animalia	Arthropoda	Trilobita	Corynexochida	Styginidae
360	Aquatic - extinct	Animalia	Chordata	Thelodonti	Shieliiformes	Shieliidae
361	Aquatic - extinct	Animalia	Chordata	Reptilia	Thalattosauria	Thalattosauridae
362	Aquatic - extinct	Animalia	Porifera	Demospongiae	Lithistida	Anthaspidellidae
363	Aquatic - extinct	Animalia	Porifera	Demospongiae	Lithistida	Anthaspidellidae
364	Aquatic - extinct	Animalia	Chordata	Thelodonti	Thelodontiformes	Phlebolepididae
365	Aquatic - extinct	Animalia	Chordata	Thelodonti	Phlebolepidiformes	Phlebolepididae
366	Aquatic - extinct	Animalia	Chordata	Thelodonti	Phlebolepidiformes	Phlebolepididae
367	Aquatic - extinct	Animalia	Chordata	Reptilia	Plesiosauria	Pliosauridae
368	Aquatic - extinct	Animalia	Arthropoda	Trilobita	Corynexochida	Scutelluidae
369	Aquatic - extinct	Animalia	Annelida	Polychaeta	Echiuroidea	Echiuridae
370	Aquatic - extinct	Animalia	Chordata	Pteraspidomorphi	Pteraspidiformes	Psammosteidae
371	Aquatic - extinct	Animalia	Porifera	Demospongiae	Orchocladina	Anthaspidellidae
372	Aquatic - extinct	Animalia	Porifera	Demospongiae	Orchocladina	Anthaspidellidae
373	Aquatic - extinct	Animalia	Porifera	Demospongiae	Lithistida	Anthaspidellidae
374	Aquatic - extinct	Animalia	Porifera	Demospongiae	Lithistida	Anthaspidellidae
375	Aquatic - extinct	Animalia	Chordata	Cephalaspidomorphi	Cephalaspidiformes	
376	Aquatic - extinct	Animalia	Chordata	Thelodonti	Shieliiformes	Shieliidae
377	Aquatic - extinct	Animalia	Arthropoda	Trilobita	Corynexochida	Styginidae
378	Aquatic - extinct	Animalia	Chordata	Reptilia	Saurischia	Spinosauridae
379	Aquatic - extinct	Animalia	Arthropoda	Trilobita	Asaphida	Nileidae
380	Aquatic - extinct	Animalia	Arthropoda	Trilobita	Asaphida	Nileidae
381	Aquatic - extinct	Animalia	Arthropoda	Trilobita	Asaphida	Nileidae
382	Aquatic - extinct	Animalia	Chordata	Thelodonti	Thelodontiformes	Talivaliidae
383	Aquatic - extinct	Animalia	Chordata	Reptilia		Tanystropheidae
384	Aquatic - extinct	Animalia	Chordata	Thelodonti	Thelodontiformes	Coelolepidae
385	Aquatic - extinct	Animalia	Problematica	Trilobozoa	Trilobozoa incertae sedis	Tribrachididae
386	Aquatic - extinct	Animalia	Problematica	Trilobozoa	Trilobozoa incertae sedis	Tribrachididae
387	Aquatic - extinct	Animalia	Chordata	Thelodonti	Thelodontiformes	Turiniidae

	G	H	I	J	K	L	M
357	*Paralejurus rehamnanus*	Skin	Adult	Wavy	10-100	100-1000	Particle removal
358	*Paralejurus spatuliformis*	Skin	Adult	Wavy		10-100	Particle removal
359	*Paralejurus tenuistriatus*	Skin	Adult	Wavy		10-100	Particle removal
360	*Paralogania menneri*	Denticle		Parallel	100-1000	1-10	Attachment
361	*Paralonectes merriami*	Tooth	Adult	Parallel			Feeding
362	*Patellispongia brosiusae*	Skin				1000-10000	Flow control
363	*Patellispongia brosiusae*	Skin				1000-10000	Nutrient intake
364	*Phlebolepis elegans*	Denticle			10-100	0.1-1	
365	*Phlebolepis elegans*	Denticle		Parallel	100-1000	1-10	Attachment
366	*Phlebolepis ornata*	Denticle		Parallel	100-1000	1-10	Attachment
367	*Polyptychodon interruptus*	Tooth	Adult	Parallel	1000-10000	100-1000	Feeding
368	*Poroscutellum porosum*	Skin	Adult	Wavy			
369	*Protechiurus edmondsi*			Parallel (Longitudinal) Perpendicular (Transverse)			
370	*Psammosteus sp.*	Denticle		Parallel		1-10	Attachment
371	*Rhopalocoelia sanxiaensis*	Skin				10000-100000	Flow control
372	*Rhopalocoelia sanxiaensis*	Skin				10000-100000	Nutrient intake
373	*Rugocoelia eganensis*	Skin				10000-100000	Flow control
374	*Rugocoelia eganensis*	Skin				10000-100000	Nutrient intake
375	*Septaspis pectinata*	Denticle		Parallel	100-1000	1-10	Attachment
376	*Shielia multispinata*	Denticle		Parallel	100-1000	1-10	Attachment
377	*Spiniscutellum umbelliferum*	Skin	Adult	Wavy			
378	*Spinosaurus*	Tooth	Adult	Parallel	10000-10000(	100-1000	Feeding
379	*Symphysurus palpebrosus*	Skin	Adult	Wavy	10-100	100-1000	Adhesion and friction
380	*Symphysurus palpebrosus*	Skin	Adult	Wavy	10-100	100-1000	Flow monitoring
381	*Symphysurus palpebrosus*	Skin	Adult	Wavy	10-100	100-1000	Respiration control
382	*Talivalia elongata*	Denticle		Parallel	100-1000	1-10	Attachment
383	*Tanystropheus longobardicu*	Tooth	Adult	Parallel	1000-10000		Feeding
384	*Thelodus laevis*	Denticle		Parallel	100-1000	1-10	Attachment
385	*Tribrachidium heraldicum*	Shield	Adult	Radial	10-100	100-1000	Chanelling
386	*Tribrachidium heraldicum*	Shield	Adult	Radial	10-100	100-1000	Food or particle collection
387	*Turinia antarctica*	Denticle		Parallel	100-1000	1-10	Attachment

	N
357	Feist R, Lerosey-Aubril R, Johnson R. 2010 Coaptative devices, enrollment, and life habits in Paralejurus, a particular case in scutelluid trilobites. Palaeobio P
358	Feist R, Lerosey-Aubril R, Johnson R. 2010 Coaptative devices, enrollment, and life habits in Paralejurus, a particular case in scutelluid trilobites. Palaeobio P
359	Feist R, Lerosey-Aubril R, Johnson R. 2010 Coaptative devices, enrollment, and life habits in Paralejurus, a particular case in scutelluid trilobites. Palaeobio P
360	Märss T. 2006 Exoskeletal ultrasculpture of early vertebrates. Journal of Vertebrate Paleontology 26, 235–252. (doi:10.1671/0272-4634(2006)26[235:EUOE
361	McCurry MR, Evans AR, Fitzgerald EMG, McHenry CR, Bevitt J, Pyenson ND. 2019 The repeated evolution of dental apicobasal ridges in aquatic-feeding mar
362	Church SB. 2017 Efficient Ornamentation in Ordovician Anthaspidellid Sponges. PC (doi:10.17161/1808.24771)
363	Church SB. 2017 Efficient Ornamentation in Ordovician Anthaspidellid Sponges. PC (doi:10.17161/1808.24771)
364	Märss T, Wilson MVH. 2008 Buccopharyngo-branchial denticles of Phlebolepis elegans Pander (Thelodonti, Agnatha). Journal of Vertebrate Paleontology 28
365	Märss T. 2006 Exoskeletal ultrasculpture of early vertebrates. Journal of Vertebrate Paleontology 26, 235–252. (doi:10.1671/0272-4634(2006)26[235:EUOE
366	Märss T. 2006 Exoskeletal ultrasculpture of early vertebrates. Journal of Vertebrate Paleontology 26, 235–252. (doi:10.1671/0272-4634(2006)26[235:EUOE
367	McCurry MR, Evans AR, Fitzgerald EMG, McHenry CR, Bevitt J, Pyenson ND. 2019 The repeated evolution of dental apicobasal ridges in aquatic-feeding mar
368	Whittington HB. 1999 Siluro-Devonian Scutelluinae (Trilobita) from the Czech Republic: morphology and classification. J. Paleontol. 73, 414–430. (doi:10.101
369	Ivantsov AYu, Vickers-Rich P, Zakrevskaya MA, Hall M. 2019 Conical Thecae of Precambrian Macroorganisms. Paleontol. J. 53, 1134–1146. (doi:10.1134/S0
370	Märss T. 2006 Exoskeletal ultrasculpture of early vertebrates. Journal of Vertebrate Paleontology 26, 235–252. (doi:10.1671/0272-4634(2006)26[235:EUOE
371	Church SB. 2017 Efficient Ornamentation in Ordovician Anthaspidellid Sponges. PC (doi:10.17161/1808.24771)
372	Church SB. 2017 Efficient Ornamentation in Ordovician Anthaspidellid Sponges. PC (doi:10.17161/1808.24771)
373	Church SB. 2017 Efficient Ornamentation in Ordovician Anthaspidellid Sponges. PC (doi:10.17161/1808.24771)
374	Church SB. 2017 Efficient Ornamentation in Ordovician Anthaspidellid Sponges. PC (doi:10.17161/1808.24771)
375	Märss T. 2006 Exoskeletal ultrasculpture of early vertebrates. Journal of Vertebrate Paleontology 26, 235–252. (doi:10.1671/0272-4634(2006)26[235:EUOE
376	Märss T. 2006 Exoskeletal ultrasculpture of early vertebrates. Journal of Vertebrate Paleontology 26, 235–252. (doi:10.1671/0272-4634(2006)26[235:EUOE
377	Whittington HB. 1999 Siluro-Devonian Scutelluinae (Trilobita) from the Czech Republic: morphology and classification. J. Paleontol. 73, 414–430. (doi:10.101
378	McCurry MR, Evans AR, Fitzgerald EMG, McHenry CR, Bevitt J, Pyenson ND. 2019 The repeated evolution of dental apicobasal ridges in aquatic-feeding mar
379	Fortey RA. 1986 The type species of the ordovician trilobitesymphysurus: systematics, functional morphology and terrace ridges. Paläontol. Z. 60, 255–275. (
380	Fortey RA. 1986 The type species of the ordovician trilobitesymphysurus: systematics, functional morphology and terrace ridges. Paläontol. Z. 60, 255–275. (
381	Fortey RA. 1986 The type species of the ordovician trilobitesymphysurus: systematics, functional morphology and terrace ridges. Paläontol. Z. 60, 255–275. (
382	Märss T. 2006 Exoskeletal ultrasculpture of early vertebrates. Journal of Vertebrate Paleontology 26, 235–252. (doi:10.1671/0272-4634(2006)26[235:EUOE
383	McCurry MR, Evans AR, Fitzgerald EMG, McHenry CR, Bevitt J, Pyenson ND. 2019 The repeated evolution of dental apicobasal ridges in aquatic-feeding mar
384	Märss T. 2006 Exoskeletal ultrasculpture of early vertebrates. Journal of Vertebrate Paleontology 26, 235–252. (doi:10.1671/0272-4634(2006)26[235:EUOE
385	Ivantsov AYu, Zakrevskaya MA. 2021 Trilobozoa, Precambrian Tri-Radial Organisms. Paleontol. J. 55, 727–741. (doi:10.1134/S0031030121070066)
386	Ivantsov AYu, Zakrevskaya MA. 2021 Trilobozoa, Precambrian Tri-Radial Organisms. Paleontol. J. 55, 727–741. (doi:10.1134/S0031030121070066)
387	Märss T. 2006 Exoskeletal ultrasculpture of early vertebrates. Journal of Vertebrate Paleontology 26, 235–252. (doi:10.1671/0272-4634(2006)26[235:EUOE

	A	B	C	D	E	F
388	Aquatic - extinct	Animalia	Chordata	Thelodonti	Thelodontiformes	Turiniidae
389	Aquatic - extinct	Animalia	Chordata	Osteostraci	Cephalaspidiformes	
390	Aquatic - extinct	Animalia	Cnidaria	Conulata		
391	Aquatic - extinct	Animalia	Chordata	Chondrichthyes		
392	Aquatic - extinct	Animalia	Chordata	Osteostraci	Tremataspidiformes	
393	Aquatic - extinct	Animalia	Chordata	Pteraspidomorphi	Eriptychiiformes	
394	Aquatic - extinct	Animalia	Chordata	Pteraspidomorphi	Cyathaspidiformes	
395	Caves	Animalia	Arthropoda	Crustacea	Isopoda	Trichoniscidae
396	Caves	Animalia	Arthropoda	Crustacea	Isopoda	Trichoniscidae
397	Humid, mostly near w	Animalia	Arthropoda	Crustacea	Isopoda	Ligiidae
398	Humid, mostly near w	Animalia	Arthropoda	Crustacea	Isopoda	Ligiidae
399	Terrestrial	Animalia	Arthropoda	Insecta	Lepidoptera	Hesperiidae
400	Terrestrial	Animalia	Chordata	Aves	Bucerotiformes	Bucerotidae
401	Terrestrial	Plantae	Angiosperms	Eudicots	Ranunculales	Ranunculaceae
402	Terrestrial	Animalia	Arthropoda	Insecta	Lepidoptera	Saturniidae
403	Terrestrial	Animalia	Arthropoda	Insecta	Lepidoptera	Saturniidae
404	Terrestrial	Animalia	Chordata	Reptilia	Squamata	
405	Terrestrial	Plantae	Angiosperms	Monocots	Asparagales	Amaryllidaceae
406	Terrestrial	Plantae	Angiosperms	Monocots	Asparagales	Amaryllidaceae
407	Terrestrial	Plantae	Angiosperms	Monocots	Asparagales	Amaryllidaceae
408	Terrestrial	Plantae	Angiosperms	Monocots	Asparagales	Amaryllidaceae
409	Terrestrial	Plantae	Angiosperms	Monocots	Asparagales	Amaryllidaceae
410	Terrestrial	Plantae	Angiosperms	Monocots	Asparagales	Amaryllidaceae
411	Terrestrial	Plantae	Angiosperms	Monocots	Asparagales	Amaryllidaceae
412	Terrestrial	Plantae	Angiosperms	Monocots	Asparagales	Amaryllidaceae
413	Terrestrial	Plantae	Angiosperms	Monocots	Asparagales	Amaryllidaceae
414	Terrestrial	Plantae	Angiosperms	Monocots	Asparagales	Amaryllidaceae
415	Terrestrial	Plantae	Angiosperms	Monocots	Asparagales	Amaryllidaceae
416	Terrestrial	Plantae	Angiosperms	Monocots	Asparagales	Amaryllidaceae
417	Terrestrial	Plantae	Angiosperms	Monocots	Asparagales	Amaryllidaceae

	G	H	I	J	K	L	M
388	*Turinia australiensis*	Denticle		Parallel	100-1000	1-10	Attachment
389	*Ungulaspis arctoa*	Denticle		Parallel	100-1000	0.1-1	Attachment
390	*Vendoconularia triradiata*			Parallel (Longitudinal) Perpendicular (Transverse)		100-1000	
391		Denticle		Parallel		1-10	Attachment
392		Denticle		Parallel		0.1-1	Attachment
393		Denticle		Parallel		1-10	Attachment
394		Denticle		Parallel		1-10	Attachment
395	*Titanethes albus*	Leg	Adult	Parallel	10-100	0.1-1	Friction and Wear reduction
396	*Titanethes albus*	Leg	Adult	Parallel	10-100	0.1-1	Particle removal
397	*Ligidium hypnorum*	Leg	Adult	Parallel	1-10	0.1-1	Friction and Wear reduction
398	*Ligidium hypnorum*	Leg	Adult	Parallel	1-10	0.1-1	Particle removal
399	*Abraximorpha davidii*	Wing	Adult	Parallel		0.1-1	Hydrophobicity
400	*Aceros waldeni*	Head	Ontogeny	Perpendicular	100-1000	1000-10000	Sexual ornamentation
401	*Adonis aestivalis*	Flower	Adult	Parallel	100-1000	0.1-1	Structural color
402	*Agapema homogena*	Wing	Adult	Perpendicular		100-1000	Acoustic decoy
403	*Aglia japonica*	Wing	Adult	Perpendicular			Acoustic decoy
404	*Alethinophidia snakes*	Tooth	Adult	Infoldings		100-1000	Structural support
405	*Allium cernuum*	Flower	Adult	Labyrinth	100-1000		
406	*Allium cernuum*	Flower	Adult	Labyrinth	100-1000		
407	*Allium cernuum*	Flower	Adult	Parallel	100-1000		
408	*Allium cernuum*	Flower	Adult	Parallel	100-1000		
409	*Allium cernuum*	Flower	Adult	Labyrinth	100-1000		
410	*Allium geyeri var. tenerum*	Flower	Adult	Wavy	100-1000		
411	*Allium geyeri var. tenerum*	Flower	Adult	Parallel	100-1000		
412	*Allium geyeri var. tenerum*	Flower	Adult	Labyrinth	100-1000		
413	*Allium stellatum*	Flower	Adult	Labyrinth	100-1000		
414	*Allium stellatum*	Flower	Adult	Wavy	100-1000		
415	*Allium stellatum*	Flower	Adult	Parallel	100-1000		
416	*Allium stellatum*	Flower	Adult	Labyrinth	100-1000		
417	*Allium textile*	Flower	Adult	Wavy	100-1000		

	N
388	Märss T. 2006 Exoskeletal ultrasculpture of early vertebrates. Journal of Vertebrate Paleontology 26, 235–252. (doi:10.1671/0272-4634(2006)26[235:EUOE
389	Märss T. 2006 Exoskeletal ultrasculpture of early vertebrates. Journal of Vertebrate Paleontology 26, 235–252. (doi:10.1671/0272-4634(2006)26[235:EUOE
390	Ivantsov AYu, Vickers-Rich P, Zakrevskaya MA, Hall M. 2019 Conical Thecae of Precambrian Macroorganisms. Paleontol. J. 53, 1134–1146. (doi:10.1134/S0
391	Märss T. 2006 Exoskeletal ultrasculpture of early vertebrates. Journal of Vertebrate Paleontology 26, 235–252. (doi:10.1671/0272-4634(2006)26[235:EUOE
392	Märss T. 2006 Exoskeletal ultrasculpture of early vertebrates. Journal of Vertebrate Paleontology 26, 235–252. (doi:10.1671/0272-4634(2006)26[235:EUOE
393	Märss T. 2006 Exoskeletal ultrasculpture of early vertebrates. Journal of Vertebrate Paleontology 26, 235–252. (doi:10.1671/0272-4634(2006)26[235:EUOE
394	Märss T. 2006 Exoskeletal ultrasculpture of early vertebrates. Journal of Vertebrate Paleontology 26, 235–252. (doi:10.1671/0272-4634(2006)26[235:EUOE
395	Vittori M. 2021 Structure of a hinge joint with textured sliding surfaces in terrestrial isopods (Crustacea: Isopoda: Oniscidea). Zoological Lett 7, 7. (doi:10.1186
396	Vittori M. 2021 Structure of a hinge joint with textured sliding surfaces in terrestrial isopods (Crustacea: Isopoda: Oniscidea). Zoological Lett 7, 7. (doi:10.1186
397	Vittori M. 2021 Structure of a hinge joint with textured sliding surfaces in terrestrial isopods (Crustacea: Isopoda: Oniscidea). Zoological Lett 7, 7. (doi:10.1186
398	Vittori M. 2021 Structure of a hinge joint with textured sliding surfaces in terrestrial isopods (Crustacea: Isopoda: Oniscidea). Zoological Lett 7, 7. (doi:10.1186
399	Fang Y, Sun G, Bi Y, Zhi H. 2015 Multiple-dimensional micro/nano structural models for hydrophobicity of butterfly wing surfaces and coupling mechanism. Sci
400	Curio E. 2004 On ornamental maturation of two Philippine hornbill species with a note on physiological colour change. J Ornithol 145. (doi:10.1007/s10336-0
401	Moyroud E et al. 2017 Disorder in convergent floral nanostructures enhances signalling to bees. Nature 550, 469–474. (doi:10.1038/nature24285)
402	Neil TR, Kennedy EE, Harris BJ, Holderied MW. 2021 Wingtip folds and ripples on saturniid moths create decoy echoes against bat biosonar. Current Biology
403	Neil TR, Kennedy EE, Harris BJ, Holderied MW. 2021 Wingtip folds and ripples on saturniid moths create decoy echoes against bat biosonar. Current Biology
404	Palci A, LeBlanc ARH, Panagiotopoulou O, Cleuren SGC, Mehari Abraha H, Hutchinson MN, Evans AR, Caldwell MW, Lee MSY. 2021 Plicidentine and the re
405	Jae Choi H, Davis AR, Hugo Cota-Sánchez J. 2011 Comparative Floral Structure of Four New World Allium (Amaryllidaceae) Species. Systematic Botany 36, 8
406	Jae Choi H, Davis AR, Hugo Cota-Sánchez J. 2011 Comparative Floral Structure of Four New World Allium (Amaryllidaceae) Species. Systematic Botany 36, 8
407	Jae Choi H, Davis AR, Hugo Cota-Sánchez J. 2011 Comparative Floral Structure of Four New World Allium (Amaryllidaceae) Species. Systematic Botany 36, 8
408	Jae Choi H, Davis AR, Hugo Cota-Sánchez J. 2011 Comparative Floral Structure of Four New World Allium (Amaryllidaceae) Species. Systematic Botany 36, 8
409	Jae Choi H, Davis AR, Hugo Cota-Sánchez J. 2011 Comparative Floral Structure of Four New World Allium (Amaryllidaceae) Species. Systematic Botany 36, 8
410	Jae Choi H, Davis AR, Hugo Cota-Sánchez J. 2011 Comparative Floral Structure of Four New World Allium (Amaryllidaceae) Species. Systematic Botany 36, 8
411	Jae Choi H, Davis AR, Hugo Cota-Sánchez J. 2011 Comparative Floral Structure of Four New World Allium (Amaryllidaceae) Species. Systematic Botany 36, 8
412	Jae Choi H, Davis AR, Hugo Cota-Sánchez J. 2011 Comparative Floral Structure of Four New World Allium (Amaryllidaceae) Species. Systematic Botany 36, 8
413	Jae Choi H, Davis AR, Hugo Cota-Sánchez J. 2011 Comparative Floral Structure of Four New World Allium (Amaryllidaceae) Species. Systematic Botany 36, 8
414	Jae Choi H, Davis AR, Hugo Cota-Sánchez J. 2011 Comparative Floral Structure of Four New World Allium (Amaryllidaceae) Species. Systematic Botany 36, 8
415	Jae Choi H, Davis AR, Hugo Cota-Sánchez J. 2011 Comparative Floral Structure of Four New World Allium (Amaryllidaceae) Species. Systematic Botany 36, 8
416	Jae Choi H, Davis AR, Hugo Cota-Sánchez J. 2011 Comparative Floral Structure of Four New World Allium (Amaryllidaceae) Species. Systematic Botany 36, 8
417	Jae Choi H, Davis AR, Hugo Cota-Sánchez J. 2011 Comparative Floral Structure of Four New World Allium (Amaryllidaceae) Species. Systematic Botany 36, 8

	A	B	C	D	E	F
418	Terrestrial	Plantae	Angiosperms	Monocots	Asparagales	Amaryllidaceae
419	Terrestrial	Plantae	Angiosperms	Monocots	Asparagales	Amaryllidaceae
420	Terrestrial	Plantae	Angiosperms	Monocots	Asparagales	Amaryllidaceae
421	Terrestrial	Plantae	Angiosperms	Monocots	Asparagales	Amaryllidaceae
422	Terrestrial	Animalia	Arthropoda	Insecta	Lepidoptera	Saturniidae
423	Terrestrial	Animalia	Arthropoda	Insecta	Lepidoptera	Saturniidae
424	Terrestrial	Animalia	Arthropoda	Insecta	Lepidoptera	Saturniidae
425	Terrestrial	Animalia	Arthropoda	Insecta	Lepidoptera	Saturniidae
426	Terrestrial	Animalia	Arthropoda	Insecta	Lepidoptera	Saturniidae
427	Terrestrial	Plantae	Angiosperms	Eudicots	Lamiales	Plantaginaceae
428	Terrestrial	Animalia	Chordata	Mammalia	Chiroptera	Vespertilionidae
429	Terrestrial	Animalia	Chordata	Mammalia	Chiroptera	Vespertilionidae
430	Terrestrial	Animalia	Arthropoda	Insecta	Lepidoptera	Nymphalidae
431	Terrestrial	Plantae	Angiosperms	Eudicots	Brassicales	Brassicaceae
432	Terrestrial	Plantae	Angiosperms	Eudicots	Brassicales	Brassicaceae
433	Terrestrial	Plantae	Angiosperms	Eudicots	Brassicales	Brassicaceae
434	Terrestrial	Plantae	Angiosperms	Eudicots	Brassicales	Brassicaceae
435	Terrestrial	Plantae	Angiosperms	Eudicots	Brassicales	Brassicaceae
436	Terrestrial	Animalia	Arthropoda	Insecta	Lepidoptera	Saturniidae
437	Terrestrial	Animalia	Arthropoda	Insecta	Lepidoptera	Nymphalidae
438	Terrestrial	Animalia	Arthropoda	Insecta	Lepidoptera	Saturniidae
439	Terrestrial	Animalia	Arthropoda	Insecta	Lepidoptera	Saturniidae
440	Terrestrial	Animalia	Arthropoda	Insecta	Lepidoptera	Saturniidae
441	Terrestrial	Animalia	Arthropoda	Insecta	Lepidoptera	Saturniidae
442	Terrestrial	Animalia	Arthropoda	Insecta	Lepidoptera	Saturniidae
443	Terrestrial	Plantae	Angiosperms	Eudicots	Astarales	Asteraceae
444	Terrestrial	Plantae	Angiosperms	Eudicots	Asterales	Asteraceae
445	Terrestrial	Plantae	Angiosperms	Eudicots	Asterales	Asteraceae
446	Terrestrial	Animalia	Arthropoda	Arachnida	Ixodida	Ixodidae
447	Terrestrial	Animalia	Arthropoda	Arachnida	Ixodida	Ixodidae
448	Terrestrial	Animalia	Arthropoda	Insecta	Lepidoptera	Nymphalidae
449	Terrestrial	Animalia	Chordata	Amphibia	Anura	Bufonidae

	G	H	I	J	K	L	M
418	*Allium textile*	Flower	Young	Parallel/ Labyrinth	100-1000		
419	*Allium textile*	Flower	Adult	Parallel	100-1000		
420	*Allium textile*	Flower	Adult	Parallel	100-1000		
421	*Allium textile*	Flower	Adult	Labyrinth	100-1000		
422	*Antheraea broschi*	Wing	Adult	Perpendicular			Acoustic decoy
423	*Antheraea pernyi*	Wing	Adult	Perpendicular	100-1000	100-1000	Acoustic decoy
424	*Antheraea polyphemus*	Wing	Adult	Perpendicular	100-1000	100-1000	Acoustic decoy
425	*Antheraea yamamai*	Wing	Adult	Perpendicular			Acoustic decoy
426	*Antheraeopsis youngi*	Wing	Adult	Perpendicular			Acoustic decoy
427	*Antirrhinum majus*	Flower	Adult	Parallel	100-1000	0.1-1	
428	*Antrozous pallidus*	Ear	Adult	Parallel/ Curved	10-100	100-1000	Sound detection
429	*Antrozous pallidus*	Ear	Adult	Parallel/ Curved	10-100	100-1000	Structural support
430	*Apatura laverna*	Wing	Adult	Parallel		0.1-1	Hydrophobicity
431	*Arabidopsis thaliana*	Flower	Adult	Labyrinth	100-1000	0.1-1	Chanelling
432	*Arabidopsis thaliana*	Flower	Adult	Parallel	100-1000	0.1-1	Chanelling
433	*Arabidopsis thaliana*	Flower	Adult	Wavy	100-1000	0.1-1	
434	*Arabidopsis thaliana*	Flower	Adult	Parallel on sides Labyrinth on apex	100-1000	0.1-1	
435	*Arabidopsis thaliana*	Flower	Adult	Wavy	100-1000	0.1-1	
436	*Archaeoattacus edwardsii*	Wing	Adult	Perpendicular		100-1000	Acoustic decoy
437	*Argynnis paphia*	Wing	Adult	Parallel	10-100	0.1-1	Hydrophobicity
438	*Arsenura armida*	Wing	Adult	Perpendicular			Acoustic decoy
439	*Athletes gigas*	Wing	Adult	Perpendicular			Acoustic decoy
440	*Attacus atlas*	Wing	Adult	Perpendicular	100-1000	100-1000	Acoustic decoy
441	*Attacus lorquinii*	Wing	Adult	Perpendicular			Acoustic decoy
442	*Aurivillius oberthuri*	Wing	Adult	Perpendicular			Acoustic decoy
443	*Bellis perennis*	Flower	Adult	Perpendicular	100-1000	1-10	
444	*Bellis perennis.*	Flower	Adult	Perpendicular	100-1000	0.1-1	Structural color
445	*Bidens ferulifolia*	Flower	Adult	Parallel	100-1000	0.1-1	Light absorption
446	*Boophilus microplus*	Skin	Adult	Parallel	1-10	10-100	
447	*Boophilus microplus*	Skin		Zig-zag	1-10	1-10	Stretching
448	*Brenthis ino*	Wing	Adult	Parallel	10-100	0.1-1	Hydrophobicity
449	*Bufo japonicus*	Toungue	Adult	Labyrinth	100-1000	0.1-1	Fluid retention

	N
418	Jae Choi H, Davis AR, Hugo Cota-Sánchez J. 2011 Comparative Floral Structure of Four New World Allium (Amaryllidaceae) Species. Systematic Botany 36, 8
419	Jae Choi H, Davis AR, Hugo Cota-Sánchez J. 2011 Comparative Floral Structure of Four New World Allium (Amaryllidaceae) Species. Systematic Botany 36, 8
420	Jae Choi H, Davis AR, Hugo Cota-Sánchez J. 2011 Comparative Floral Structure of Four New World Allium (Amaryllidaceae) Species. Systematic Botany 36, 8
421	Jae Choi H, Davis AR, Hugo Cota-Sánchez J. 2011 Comparative Floral Structure of Four New World Allium (Amaryllidaceae) Species. Systematic Botany 36, 8
422	Neil TR, Kennedy EE, Harris BJ, Holderied MW. 2021 Wingtip folds and ripples on saturniid moths create decoy echoes against bat biosonar. Current Biology
423	Neil TR, Kennedy EE, Harris BJ, Holderied MW. 2021 Wingtip folds and ripples on saturniid moths create decoy echoes against bat biosonar. Current Biology
424	Neil TR, Kennedy EE, Harris BJ, Holderied MW. 2021 Wingtip folds and ripples on saturniid moths create decoy echoes against bat biosonar. Current Biology
425	Neil TR, Kennedy EE, Harris BJ, Holderied MW. 2021 Wingtip folds and ripples on saturniid moths create decoy echoes against bat biosonar. Current Biology
426	Neil TR, Kennedy EE, Harris BJ, Holderied MW. 2021 Wingtip folds and ripples on saturniid moths create decoy echoes against bat biosonar. Current Biology
427	Antoniou Kourounioti RL et al. 2013 Buckling as an origin of ordered cuticular patterns in flower petals. Journal of The Royal Society Interface 10, 20120847.
428	Keeley BW, Keeley ATH, Houlahan P. 2018 Ridge number in bat ears is related to both guild membership and ear length. PLoS ONE 13, e0200255. (doi:10.
429	Keeley BW, Keeley ATH, Houlahan P. 2018 Ridge number in bat ears is related to both guild membership and ear length. PLoS ONE 13, e0200255. (doi:10.
430	Fang Y, Sun G, Bi Y, Zhi H. 2015 Multiple-dimensional micro/nano structural models for hydrophobicity of butterfly wing surfaces and coupling mechanism. Sci
431	Li-Beisson Y, Pollard M, Sauveplane V, Pinot F, Ohlrogge J, Beisson F. 2009 Nanoridges that characterize the surface morphology of flowers require the synt
432	Li-Beisson Y, Pollard M, Sauveplane V, Pinot F, Ohlrogge J, Beisson F. 2009 Nanoridges that characterize the surface morphology of flowers require the synt
433	Hong L, Brown J, Segerson NA, Rose JKC, Roeder AHK. 2017 CUTIN SYNTHASE 2 Maintains Progressively Developing Cuticular Ridges in Arabidopsis Sepa
434	Hong L, Brown J, Segerson NA, Rose JKC, Roeder AHK. 2017 CUTIN SYNTHASE 2 Maintains Progressively Developing Cuticular Ridges in Arabidopsis Sepa
435	Hong L, Brown J, Segerson NA, Rose JKC, Roeder AHK. 2017 CUTIN SYNTHASE 2 Maintains Progressively Developing Cuticular Ridges in Arabidopsis Sepa
436	Neil TR, Kennedy EE, Harris BJ, Holderied MW. 2021 Wingtip folds and ripples on saturniid moths create decoy echoes against bat biosonar. Current Biology
437	Fang Y, Sun G, Bi Y, Zhi H. 2015 Multiple-dimensional micro/nano structural models for hydrophobicity of butterfly wing surfaces and coupling mechanism. Sci
438	Neil TR, Kennedy EE, Harris BJ, Holderied MW. 2021 Wingtip folds and ripples on saturniid moths create decoy echoes against bat biosonar. Current Biology
439	Neil TR, Kennedy EE, Harris BJ, Holderied MW. 2021 Wingtip folds and ripples on saturniid moths create decoy echoes against bat biosonar. Current Biology
440	Neil TR, Kennedy EE, Harris BJ, Holderied MW. 2021 Wingtip folds and ripples on saturniid moths create decoy echoes against bat biosonar. Current Biology
441	Neil TR, Kennedy EE, Harris BJ, Holderied MW. 2021 Wingtip folds and ripples on saturniid moths create decoy echoes against bat biosonar. Current Biology
442	Neil TR, Kennedy EE, Harris BJ, Holderied MW. 2021 Wingtip folds and ripples on saturniid moths create decoy echoes against bat biosonar. Current Biology
443	Huang X, Hai Y, Xie W-H. 2017 Anisotropic cell growth-regulated surface micropatterns in flower petals. Theoretical and Applied Mechanics Letters 7, 169–17
444	Kooi CJ, Wilts BD, Leertouwer HL, Staal M, Elzenga JTM, Stavenga DG. 2014 Iridescent flowers? Contribution of surface structures to optical signaling. New P
445	Schulte AJ, Mail M, Hahn LA, Barthlott W. 2019 Ultraviolet patterns of flowers revealed in polymer replica – caused by surface architecture. Beilstein J. Nanote
446	Kang YB, Jang DH. 1985 Scanning electron microscopic observations on the surface structure of the tick Boophilus microplus(Canestrini, 1887) female specin
447	Hackman RH. Structure and function in tick cuticle. Annual review of entomology. 1982 Jan;27(1):75-95.
448	Fang Y, Sun G, Bi Y, Zhi H. 2015 Multiple-dimensional micro/nano structural models for hydrophobicity of butterfly wing surfaces and coupling mechanism. Sci
449	Iwasaki SI, Kobayashi K. Fine structure of the dorsal tongue surface in the Japanese toad, Bufo japonicus (Anura, Bufonidae). Zoological science. 1988 Apr;

	A	B	C	D	E	F
450	Terrestrial	Animalia	Arthropoda	Insecta	Lepidoptera	Saturniidae
451	Terrestrial	Animalia	Arthropoda	Insecta	Lepidoptera	Papilionidae
452	Terrestrial	Animalia	Arthropoda	Insecta	Diptera	
453	Terrestrial	Animalia	Arthropoda	Insecta	Lepidoptera	Saturniidae
454	Terrestrial	Animalia	Chordata	Mammalia	Chiroptera	Phyllostomidae
455	Terrestrial	Animalia	Chordata	Mammalia	Chiroptera	Phyllostomidae
456	Terrestrial	Animalia	Nematoda	Chromadorea	Rhabditida	Cephalobidae
457	Terrestrial	Animalia	Nematoda	Chromadorea	Rhabditida	Cephalobidae
458	Terrestrial	Animalia	Chordata	Reptilia	Squamata	Boidae
459	Terrestrial	Animalia	Arthropoda	Insecta	Lepidoptera	Nymphalidae
460	Terrestrial	Animalia	Chordata	Mammalia	Chiroptera	Phyllostomidae
461	Terrestrial	Animalia	Chordata	Mammalia	Chiroptera	Phyllostomidae
462	Terrestrial	Fungi	Zygomycota	Mucoromycetes	Mucorales	Syncephalastraceae
463	Terrestrial	Animalia	Arthropoda	Insecta	Lepidoptera	Saturniidae
464	Terrestrial	Animalia	Arthropoda	Insecta	Lepidoptera	Nymphalidae
465	Terrestrial	Animalia	Arthropoda	Insecta	Lepidoptera	Pieridae
466	Terrestrial	Animalia	Arthropoda	Insecta	Lepidoptera	Saturniidae
467	Terrestrial	Animalia	Arthropoda	Insecta	Hemiptera	Coreidae
468	Terrestrial	Animalia	Chordata	Mammalia	Chiroptera	Vespertilionidae
469	Terrestrial	Animalia	Chordata	Mammalia	Chiroptera	Vespertilionidae
470	Terrestrial	Animalia	Chordata	Mammalia	Chiroptera	Vespertilionidae
471	Terrestrial	Animalia	Chordata	Mammalia	Chiroptera	Vespertilionidae
472	Terrestrial	Animalia	Arthropoda	Arachnida	Araneae	Trechaleidae
473	Terrestrial	Plantae	Angiosperms	Eudicots	Ericales	Primulaceae
474	Terrestrial	Plantae	Angiosperms	Eudicots	Ericales	Primulaceae
475	Terrestrial	Animalia	Arthropoda	Crustacea	Isopoda	Cylisticidae
476	Terrestrial	Animalia	Arthropoda	Crustacea	Isopoda	Cylisticidae
477	Terrestrial	Animalia	Arthropoda	Insecta	Lepidoptera	Nymphalidae
478	Terrestrial	Animalia	Arthropoda	Insecta	Diptera	Bibionidae
479	Terrestrial	Animalia	Nematoda	Secernentea	Tylenchida	Nematoda
480	Terrestrial	Animalia	Nematoda	Secernentea	Tylenchida	Dolichodoridae
481	Terrestrial	Animalia	Chordata	Reptilia	Squamata	Colubridae
482	Terrestrial	Animalia	Chordata	Reptilia	Squamata	Colubridae

	G	H	I	J	K	L	M
450	*Bunaea aslauga*	Wing	Adult	Perpendicular			Acoustic decoy
451	*Byasa alcinous*	Wing	Adult	Parallel	10-100	0.1-1	Hydrophobicity
452	*C. testaceipes*	Eye	Adult	Labyrinth		0.1-1	
453	*Caio romulus*	Wing	Adult	Perpendicular			Acoustic decoy
454	*Centurio senex*	Face	Adult	Wavy	10-100	1000-10000	Courtship
455	*Centurio senex*	Face	Adult	Wavy	10-100	1000-10000	Sound detection
456	*Cervidellus spitzbergensis*	Skin	Adult	Parallel	0.1-1	0.1-1	
457	*Cervidellus spitzbergensis*	Skin	Adult	Perpendicular	0.1-1	1-10	
458	*Charina bottae*	Skin	Adult	Parallel	100-1000	10-100	Friction and Wear reduction
459	*Childrena zenobia*	Wing	Adult	Parallel		0.1-1	Hydrophobicity
460	*Chrotopterus auritus*	Ear	Adult	Parallel/ Curved	100-1000	100-1000	Sound detection
461	*Chrotopterus auritus*	Ear	Adult	Parallel/ Curved	100-1000	100-1000	Structural support
462	*Circinella umbellata van Tieg*	Sporangium		Wavy	0.1-1	0.1-1	
463	*Cirina forda*	Wing	Adult	Perpendicular			Acoustic decoy
464	*Coenonympha amaryllis*	Wing	Adult	Parallel		0.1-1	Hydrophobicity
465	*Colias erate*	Wing	Adult	Parallel	10-100	0.1-1	Hydrophobicity
466	*Copaxa syntheratoides*	Wing	Adult	Perpendicular			Acoustic decoy
467	*Coreus marginatus*	Leg	Adult	Parallel	10-100	1-10	Adhesion and friction
468	*Corynorhinus rafinesquii*	Ear	Adult	Parallel/ Curved	100-1000	100-1000	Sound detection
469	*Corynorhinus rafinesquii*	Ear	Adult	Parallel/ Curved	100-1000	100-1000	Structural support
470	*Corynorhinus townsendii*	Ear	Adult	Parallel/ Curved	10-100	100-1000	Sound detection
471	*Corynorhinus townsendii*	Ear	Adult	Parallel/ Curved	10-100	100-1000	Structural support
472	*Cupiennius salei*	Eye	Adult	Parallel	10-100		Structural color
473	*Cyclamen persicum*	Flower	Adult	Parallel	100-1000	0.1-1	Hydrophobicity
474	*Cyclamen persicum*	Flower	Adult	Parallel	100-1000	0.1-1	Insect adhesion
475	*Cylisticus convexus*	Leg	Adult	Parallel	10-100	0.1-1	Friction and Wear reduction
476	*Cylisticus convexus*	Leg	Adult	Parallel	10-100	0.1-1	Particle removal
477	*Damora sagana*	Wing	Adult	Parallel	10-100	0.1-1	Hydrophobicity
478	*Dilophus orbatus*	Eye	Adult	Labyrinth		0.01-0.1	
479	*Divittus labiatus*	Skin	Adult	Parallel	0.1-1	1-10	
480	*Dolichorhynchus*	Skin	Adult	Parallel			
481	*Elaphe climacophora*	Tongue	Adult	Labyrinth	1000-10000	0.1-1	Fluid retention
482	*Elaphe climacophora*	Tongue	Adult	Labyrinth	1000-10000	0.1-1	Flow control

	N
450	Neil TR, Kennedy EE, Harris BJ, Holderied MW. 2021 Wingtip folds and ripples on saturniid moths create decoy echoes against bat biosonar. Current Biology
451	Fang Y, Sun G, Bi Y, Zhi H. 2015 Multiple-dimensional micro/nano structural models for hydrophobicity of butterfly wing surfaces and coupling mechanism. Sci
452	Anderson MS, Gaimari SD. 2003 Raman-atomic force microscopy of the ommatidial surfaces of Dipteran compound eyes. Journal of Structural Biology 142, 3
453	Neil TR, Kennedy EE, Harris BJ, Holderied MW. 2021 Wingtip folds and ripples on saturniid moths create decoy echoes against bat biosonar. Current Biology
454	Rodríguez-Herrera B, Sánchez-Calderón R, Madrigal-Elizondo V, Rodríguez P, Villalobos J, Hernández E, Zamora-Mejías D, Gessinger G, Tschapka M. 2020
455	Rodríguez-Herrera B, Sánchez-Calderón R, Madrigal-Elizondo V, Rodríguez P, Villalobos J, Hernández E, Zamora-Mejías D, Gessinger G, Tschapka M. 2020
456	Boström S. 1987 A new terrestrial nematode species (Rhabditida: Cephalobidae) from spitzbergen. Polar Biol 7, 375–378. (doi:10.1007/BF00293227)
457	Boström S. 1987 A new terrestrial nematode species (Rhabditida: Cephalobidae) from spitzbergen. Polar Biol 7, 375–378. (doi:10.1007/BF00293227)
458	Martinez A, Nguyen D, Basson MS, Medina J, Irschick DJ, Baeckens S. 2021 Quantifying surface topography of biological systems from 3D scans. Methods E
459	Fang Y, Sun G, Bi Y, Zhi H. 2015 Multiple-dimensional micro/nano structural models for hydrophobicity of butterfly wing surfaces and coupling mechanism. Sci
460	Keeley BW, Keeley ATH, Houlahan P. 2018 Ridge number in bat ears is related to both guild membership and ear length. PLoS ONE 13, e0200255. (doi:10.
461	Keeley BW, Keeley ATH, Houlahan P. 2018 Ridge number in bat ears is related to both guild membership and ear length. PLoS ONE 13, e0200255. (doi:10.
462	Little SA, Young TWK. 1987 Light and electron microscopy of Circinella umbellata (Mucorales). Transactions of the British Mycological Society 88, 441–449. (
463	Neil TR, Kennedy EE, Harris BJ, Holderied MW. 2021 Wingtip folds and ripples on saturniid moths create decoy echoes against bat biosonar. Current Biology
464	Fang Y, Sun G, Bi Y, Zhi H. 2015 Multiple-dimensional micro/nano structural models for hydrophobicity of butterfly wing surfaces and coupling mechanism. Sci
465	Fang Y, Sun G, Bi Y, Zhi H. 2015 Multiple-dimensional micro/nano structural models for hydrophobicity of butterfly wing surfaces and coupling mechanism. Sci
466	Neil TR, Kennedy EE, Harris BJ, Holderied MW. 2021 Wingtip folds and ripples on saturniid moths create decoy echoes against bat biosonar. Current Biology
467	Rebora M, Salerno G, Piersanti S, Gorb EV, Gorb SN. 2021 Attachment devices and the tarsal gland of the bug Coreus marginatus (Hemiptera: Coreidae). Z
468	Keeley BW, Keeley ATH, Houlahan P. 2018 Ridge number in bat ears is related to both guild membership and ear length. PLoS ONE 13, e0200255. (doi:10.
469	Keeley BW, Keeley ATH, Houlahan P. 2018 Ridge number in bat ears is related to both guild membership and ear length. PLoS ONE 13, e0200255. (doi:10.
470	Keeley BW, Keeley ATH, Houlahan P. 2018 Ridge number in bat ears is related to both guild membership and ear length. PLoS ONE 13, e0200255. (doi:10.
471	Keeley BW, Keeley ATH, Houlahan P. 2018 Ridge number in bat ears is related to both guild membership and ear length. PLoS ONE 13, e0200255. (doi:10.
472	Politi Y, Bertinetti L, Fratzl P, Barth FG. 2021 The spider cuticle: a remarkable material toolbox for functional diversity. Phil. Trans. R. Soc. A. 379, 20200332.
473	Prüm B, Seidel R, Bohn HF, Speck T. 2012 Plant surfaces with cuticular folds are slippery for beetles. J. R. Soc. Interface. 9, 127–135. (doi:10.1098/rsif.2011
474	Prüm B, Seidel R, Bohn HF, Speck T. 2012 Plant surfaces with cuticular folds are slippery for beetles. J. R. Soc. Interface. 9, 127–135. (doi:10.1098/rsif.2011
475	Vittori M. 2021 Structure of a hinge joint with textured sliding surfaces in terrestrial isopods (Crustacea: Isopoda: Oniscidea). Zoological Lett 7, 7. (doi:10.1186
476	Vittori M. 2021 Structure of a hinge joint with textured sliding surfaces in terrestrial isopods (Crustacea: Isopoda: Oniscidea). Zoological Lett 7, 7. (doi:10.1186
477	Fang Y, Sun G, Bi Y, Zhi H. 2015 Multiple-dimensional micro/nano structural models for hydrophobicity of butterfly wing surfaces and coupling mechanism. Sci
478	Anderson MS, Gaimari SD. 2003 Raman-atomic force microscopy of the ommatidial surfaces of Dipteran compound eyes. Journal of Structural Biology 142, 3
479	Jairajpuri MS. 1984 Some studies on Tylenchorhynchinae: proposal ofDivittus n.g. (Nematoda: Tylenchida). Syst Parasitol 6, 107–112. (doi:10.1007/BF0218
480	Jairajpuri MS, Hunt DJ. 1984 The taxonomy of Tylenchorhynchinae (Nematoda: Tylenchida) with longitudinal lines and ridges. Syst Parasitol 6, 261–268. (doi:
481	Iwasaki S-I, Yoshizawa H, Kawahara I. 1996 Three-dimensional ultrastructure of the surface of the tongue of the rat snake,Elaphe climacophora. Anat. Rec. 2
482	Iwasaki S-I, Yoshizawa H, Kawahara I. 1996 Three-dimensional ultrastructure of the surface of the tongue of the rat snake,Elaphe climacophora. Anat. Rec. 2

	A	B	C	D	E	F
483	Terrestrial	Animalia	Chordata	Reptilia	Squamata	Colubridae
484	Terrestrial	Plantae	Angiosperms	Eudicots	Ranunculales	Papaveraceae
485	Terrestrial	Plantae	Angiosperms	Eudicots	Ranunculales	Papaveraceae
486	Terrestrial	Animalia	Arthropoda	Insecta	Lepidoptera	Saturniidae
487	Terrestrial	Plantae	Angiosperms	Eudicots	Gentianales	Gentianaceae
488	Terrestrial	Animalia	Arthropoda	Insecta	Lepidoptera	Lycaenidae
489	Terrestrial	Animalia	Arthropoda	Insecta	Lepidoptera	Nymphalidae
490	Terrestrial	Plantae	Angiosperms	Eudicots	Gentianales	Rubiaceae
491	Terrestrial	Animalia	Platyhelminthes	Trematoda		Gastrothylacidae
492	Terrestrial	Plantae	Angiosperms	Eudicots	Asterales	Asteraceae
493	Terrestrial	Animalia	Arthropoda	Insecta	Lepidoptera	Pieridae
494	Terrestrial	Animalia	Arthropoda	Insecta	Lepidoptera	Saturniidae
495	Terrestrial	Plantae	Angiosperms	Eudicots	Malvales	Neuradaceae
496	Terrestrial	Plantae	Angiosperms	Eudicots	Asterales	Asteraceae
497	Terrestrial	Animalia	Mollusca	Gastropoda	Stylommatophora	Helicidae
498	Terrestrial	Animalia	Chordata	Mammalia	Rodentia	Bathyergidae
499	Terrestrial	Animalia	Chordata	Mammalia	Rodentia	Bathyergidae
500	Terrestrial	Animalia	Chordata	Mammalia	Rodentia	Bathyergidae
501	Terrestrial	Plantae	Angiosperms	Eudicots	Malpighiales	Euphorbiaceae
502	Terrestrial	Plantae	Angiosperms	Eudicots	Malpighiales	Euphorbiaceae
503	Terrestrial	Plantae	Angiosperms	Eudicots	Malvaceae	Malvales
504	Terrestrial	Plantae	Angiosperms	Eudicots	Malvales	Malvaceae
505	Terrestrial	Plantae	Angiosperms	Eudicots	Malvales	Malvaceae
506	Terrestrial	Plantae	Angiosperms	Eudicots	Malvales	Malvaceae
507	Terrestrial	Plantae	Angiosperms	Eudicots	Malvales	Malvaceae
508	Terrestrial	Animalia	Chordata	Mammalia	Primates	Hominidae
509	Terrestrial	Animalia	Chordata	Mammalia	Primates	Hominidae
510	Terrestrial	Animalia	Chordata	Mammalia	Primates	Hominidae
511	Terrestrial	Animalia	Arthropoda	Arachnida	Ixodida	Ixodidae
512	Terrestrial	Animalia	Arthropoda	Insecta	Lepidoptera	Saturniidae
513	Terrestrial	Animalia	Arthropoda	Arachnida	Ixodida	Ixodidae

	G	H	I	J	K	L	M
483	*Elaphe climacophora*	Tongue	Adult	Labyrinth	1000-10000	0.1-1	Food or particle collection
484	*Eschscholzia californica*	Flower	Juvenile	Parallel	100-1000	0.1-1	Structural color
485	*Eschscholzia californica*	Flower	Adult	Parallel	100-1000	1-10	Structural color
486	*Eupackardia calleta*	Wing	Adult	Perpendicular			Acoustic decoy
487	*Eustoma grandiflorum*	Flower	Adult	Parallel and Labyrinth	100-1000	1-10	
488	*Everes argiades*	Wing	Adult	Parallel	10-100	0.1-1	Hydrophobicity
489	*Fabriciana niobe*	Wing	Adult	Parallel	10-100	0.1-1	Hydrophobicity
490	*Galium uruguayense*	Fruit		Parallel		1-10	
491	*Gastrothylux crumeni*	Skin		Perpendicular	1-10	10-100	Attachment
492	*Gazania krebsiana*	Flower	Adult	Perpendicular	100-1000		
493	*Gonepteryx rhamni*	Wing	Adult	Parallel	10-100	0.1-1	Hydrophobicity
494	*Gonimbrasia zambesina*	Wing	Adult	Perpendicular			Acoustic decoy
495	*Grielum humifusum*	Flower	Adult	Parallel		0.1-1	Structural color
496	*Helianthus annuus*	Flower	Adult	Parallel on sides<br>Labyrinth on apex	1000-10000		
497	*Helix aspersa*	Skin	Adult		10-100	100-1000	Chanelling
498	*Heterocephalus glaber*	Skin	Adult	Wavy	100-1000	100-1000	Friction and Wear reduction
499	*Heterocephalus glaber*	Skin	Adult	Wavy	100-1000	100-1000	Structural support
500	*Heterocephalus glaber*	Skin	Adult	Wavy	100-1000	100-1000	Thermoregulation
501	*Hevea brasiliensis*	Leaf	Adult	Labyrinth	10000-100000	0.1-1	Insect adhesion
502	*Hevea brasiliensis*	Leaf	Adult	Labyrinth	10000-100000	0.1-1	Structural support
503	*Hibiscus trionum*	Flower	Adult	Parallel	100-1000	1-10	Structural color
504	*Hibiscus trionum*	Flower	Adult	Parallel	100-1000	1-10	Structural color
505	*Hibiscus trionum*	Flower	Adult	Parallel	100-1000	0.1-1	Structural color
506	*Hibiscus trionum*	Flower	Adult	Parallel	100-1000	0.1-1	Structural color
507	*Hibiscus trionum*	Flower	Adult	Parallel	100-1000	0.1-1	Structural color
508	*Homo sapiens*	Finger	Adult	Wavy	1000-10000	1000-10000	Adhesion and friction
509	*Homo sapiens*	Finger	Adult	Wavy	1000-10000	1000-10000	Chanelling
510	*Homo sapiens*	Skin	Adult	Microrelief	10000-100000		Structural support
511	*Hyalomma asiaticum*	Skin		Zig-zag	1-10	10-100	Stretching
512	*Hyalophora cecropia*	Wing	Adult	Perpendicular			Acoustic decoy
513	*Ixodes ricinus*	Leg	Adult	Parallel	1-10	0.1-1	Adhesion and friction

	N
483	Iwasaki S-I, Yoshizawa H, Kawahara I. 1996 Three-dimensional ultrastructure of the surface of the tongue of the rat snake,Elaphe climacophora. Anat. Rec. 2
484	Wilts BD, Rudall PJ, Moyroud E, Gregory T, Ogawa Y, Vignolini S, Steiner U, Glover BJ. 2018 Ultrastructure and optics of the prism-like petal epidermal cells o
485	Wilts BD, Rudall PJ, Moyroud E, Gregory T, Ogawa Y, Vignolini S, Steiner U, Glover BJ. 2018 Ultrastructure and optics of the prism-like petal epidermal cells o
486	Neil TR, Kennedy EE, Harris BJ, Holderied MW. 2021 Wingtip folds and ripples on saturniid moths create decoy echoes against bat biosonar. Current Biology
487	Huang X, Hai Y, Xie W-H. 2017 Anisotropic cell growth-regulated surface micropatterns in flower petals. Theoretical and Applied Mechanics Letters 7, 169–17
488	Fang Y, Sun G, Bi Y, Zhi H. 2015 Multiple-dimensional micro/nano structural models for hydrophobicity of butterfly wing surfaces and coupling mechanism. Sci
489	Fang Y, Sun G, Bi Y, Zhi H. 2015 Multiple-dimensional micro/nano structural models for hydrophobicity of butterfly wing surfaces and coupling mechanism. Sci
490	De Toni KLG, Mariath JEA. 2011 Developmental Anatomy and Morphology of the Flowers and Fruits of Species from Galium and Relbunium (Rubieae, Rubiac
491	Tandon V, Maitra SC. Stereocan observations on the surface topography of Gastrothylax crumenifer (Creplin, 1847) Poirier, 1883 and Paramphistomum epicli
492	Whitney HM, Bennett KMV, Dorling M, Sandbach L, Prince D, Chittka L, Glover BJ. 2011 Why do so many petals have conical epidermal cells? Annals of Bota
493	Fang Y, Sun G, Bi Y, Zhi H. 2015 Multiple-dimensional micro/nano structural models for hydrophobicity of butterfly wing surfaces and coupling mechanism. Sci
494	Neil TR, Kennedy EE, Harris BJ, Holderied MW. 2021 Wingtip folds and ripples on saturniid moths create decoy echoes against bat biosonar. Current Biology
495	Moyroud E et al. 2017 Disorder in convergent floral nanostructures enhances signalling to bees. Nature 550, 469–474. (doi:10.1038/nature24285)
496	Whitney HM, Bennett KMV, Dorling M, Sandbach L, Prince D, Chittka L, Glover BJ. 2011 Why do so many petals have conical epidermal cells? Annals of Bota
497	Machin J. The evaporation of water from Helix aspersa. IV. Loss from the mantle of the inactive snail. J Exp Biol. 1966 Oct;45(2):269-78
498	Daly TJM, Buffenstein R. 1998 Skin morphology and its role in thermoregulation in mole-rats, Heterocephalus glaber and Cryptomys hottentotus. J Anatomy 1
499	Daly TJM, Buffenstein R. 1998 Skin morphology and its role in thermoregulation in mole-rats, Heterocephalus glaber and Cryptomys hottentotus. J Anatomy 1
500	Daly TJM, Buffenstein R. 1998 Skin morphology and its role in thermoregulation in mole-rats, Heterocephalus glaber and Cryptomys hottentotus. J Anatomy 1
501	Surapaneni VA, Bold G, Speck T, Thielen M. In press. Spatio-temporal development of cuticular ridges on leaf surfaces of Hevea brasiliensis alters insect atta
502	Surapaneni VA, Bold G, Speck T, Thielen M. In press. Spatio-temporal development of cuticular ridges on leaf surfaces of Hevea brasiliensis alters insect atta
503	Chen C, Airoldi CA, Lugo CA, Bay RK, Glover BJ, Crosby AJ. 2021 Flower Inspiration: Broad-Angle Structural Color through Tunable Hierarchical Wrinkles in T
504	Airoldi CA, Lugo CA, Wightman R, Glover BJ, Robinson S. 2021 Mechanical buckling can pattern the light-diffracting cuticle of Hibiscus trionum. Cell Reports 3
505	Kooi CJ, Wilts BD, Leertouwer HL, Staal M, Elzenga JTM, Stavenga DG. 2014 Iridescent flowers? Contribution of surface structures to optical signaling. New F
506	Antoniou Kourounioti RL et al. 2013 Buckling as an origin of ordered cuticular patterns in flower petals. Journal of The Royal Society Interface 10, 20120847.
507	Moyroud E et al. 2017 Disorder in convergent floral nanostructures enhances signalling to bees. Nature 550, 469–474. (doi:10.1038/nature24285)
508	Changizi M, Weber R, Kotecha R, Palazzo J. 2011 Are Wet-Induced Wrinkled Fingers Primate Rain Treads? Brain Behav Evol 77, 286–290. (doi:10.1159/000
509	Changizi M, Weber R, Kotecha R, Palazzo J. 2011 Are Wet-Induced Wrinkled Fingers Primate Rain Treads? Brain Behav Evol 77, 286–290. (doi:10.1159/000
510	Limbert G, Kuhl E. 2018 On skin microrelief and the emergence of expression micro-wrinkles. Soft Matter 14, 1292–1300. (doi:10.1039/C7SM01969F)
511	Hackman RH. Structure and function in tick cuticle. Annual review of entomology. 1982 Jan;27(1):75-95.
512	Neil TR, Kennedy EE, Harris BJ, Holderied MW. 2021 Wingtip folds and ripples on saturniid moths create decoy echoes against bat biosonar. Current Biology
513	Voigt D, Gorb S. 2017 Functional morphology of tarsal adhesive pads and attachment ability in ticks Ixodes ricinus (Arachnida, Acari, Ixodidae). Journal of Exp

	A	B	C	D	E	F
514	Terrestrial	Animalia	Arthropoda	Arachnida	Ixodida	Ixodidae
515	Terrestrial	Animalia	Arthropoda	Arachnida	Ixodida	Ixodidae
516	Terrestrial	Animalia	Arthropoda	Arachnida	Ixodida	Ixodidae
517	Terrestrial	Plantae	Angiosperms	Eudicots	Saxifragales	Crassulaceae
518	Terrestrial	Animalia	Arthropoda	Arachnida	Araneae	Theraphosidae
519	Terrestrial	Animalia	Arthropoda	Arachnida	Araneae	Theraphosidae
520	Terrestrial	Animalia	Chordata	Reptilia	Squamata	Colubridae
521	Terrestrial	Plantae	Angiosperms	Eudicots	Fabales	Fabaceae
522	Terrestrial	Animalia	Arthropoda	Insecta	Lepidoptera	Saturniidae
523	Terrestrial	Animalia	Arthropoda	Collembola	Entomobryomorpha	Entomobryidae
524	Terrestrial	Plantae	Angiosperms	Monocots	Asparagales	Amaryllidaceae
525	Terrestrial	Animalia	Arthropoda	Insecta	Lepidoptera	Nymphalidae
526	Terrestrial	Animalia	Arthropoda	Insecta	Lepidoptera	Nymphalidae
527	Terrestrial	Plantae	Angiosperms	Eudicots	Sapindales	Sapindaceae
528	Terrestrial	Plantae	Angiosperms	Eudicots	Sapindales	Sapindaceae
529	Terrestrial	Animalia	Arthropoda	Insecta	Lepidoptera	Saturniidae
530	Terrestrial	Animalia	Arthropoda	Insecta	Lepidoptera	Saturniidae
531	Terrestrial	Animalia	Chordata	Mammalia	Chiroptera	Phyllostomidae
532	Terrestrial	Animalia	Chordata	Mammalia	Chiroptera	Phyllostomidae
533	Terrestrial	Animalia	Chordata	Mammalia	Chiroptera	Phyllostomidae
534	Terrestrial	Animalia	Chordata	Mammalia	Chiroptera	Phyllostomidae
535	Terrestrial	Animalia	Arthropoda	Insecta	Lepidoptera	Nymphalidae
536	Terrestrial	Animalia	Chordata	Mammalia	Proboscidea	Elephantidae
537	Terrestrial	Animalia	Chordata	Mammalia	Proboscidea	Elephantidae
538	Terrestrial	Animalia	Chordata	Mammalia	Proboscidea	Elephantidae
539	Terrestrial	Animalia	Arthropoda	Insecta	Lepidoptera	Saturniidae
540	Terrestrial	Animalia	Chordata	Mammalia	Chiroptera	Phyllostomidae
541	Terrestrial	Animalia	Chordata	Mammalia	Chiroptera	Phyllostomidae
542	Terrestrial	Plantae	Angiosperms	Eudicots	Asterales	Asteraceae
543	Terrestrial	Animalia	Chordata	Reptilia	Squamata	Uropeltidae
544	Terrestrial	Animalia	Chordata	Reptilia	Squamata	Uropeltidae
545	Terrestrial	Animalia	Chordata	Reptilia	Squamata	Uropeltidae
546	Terrestrial	Animalia	Chordata	Reptilia	Squamata	Uropeltidae

	G	H	I	J	K	L	M
514	*Ixodes ricinus*	Leg	Adult	Parallel	1-10	0.1-1	Chanelling
515	*Ixodus canisuga*	Skin		Zig-zag	1-10	10-100	Stretching
516	*Ixodus ricinus*	Skin		Zig-zag	1-10	10-100	Stretching
517	*Kalanchoe blossfeldiana*	Flower	Adult	Parallel	100-1000	0.1-1	
518	*Lampropelma violaceopes*	Hair	Adult	Parallel	100-1000		Structural color
519	*Lampropelma violaceopes*	Hair	Adult	Parallel	100-1000		Structural color
520	*Lampropeltis getula*	Skin	Adult	Parallel	1000-10000	10-100	Friction and Wear reduction
521	*Lathyrus aureus*	Flower	Adult	Parallel	100-1000	0.1-1	Structural color
522	*Lemaireia luteopeplus*	Wing	Adult	Perpendicular			Acoustic decoy
523	*Lepidocyrtus curvicollis*	Scale	Adult			0.1-1	Fluid exchange
524	*Leucocoryne purpurea*	Flower	Adult	Parallel	100-1000	0.1-1	Structural color
525	*Limenitis doerriesi*	Wing	Adult	Parallel		0.1-1	Hydrophobicity
526	*Limenitis populi*	Wing	Adult	Parallel	10-100	0.1-1	Hydrophobicity
527	*Litchi chinensis*	Leaf	Adult	Labyrinth	10000-10000(	0.1-1	Hydrophobicity
528	*Litchi chinensis*	Leaf	Adult	Labyrinth	10000-10000(	0.1-1	Insect adhesion
529	*Lobobunaea saturnus*	Wing	Adult	Perpendicular			Acoustic decoy
530	*Loepa megacore*	Wing	Adult	Perpendicular			Acoustic decoy
531	*Lophostoma brasiliense*	Ear	Adult	Parallel/ Curved	10-100	100-1000	Sound detection
532	*Lophostoma brasiliense*	Ear	Adult	Parallel/ Curved	10-100	100-1000	Structural support
533	*Lophostoma silvicolum*	Ear	Adult	Parallel/ Curved	10-100	100-1000	Sound detection
534	*Lophostoma silvicolum*	Ear	Adult	Parallel/ Curved	10-100	100-1000	Structural support
535	*Lopinga achine*	Wing	Adult	Parallel	10-100	0.1-1	Hydrophobicity
536	*Loxodonta africana*	Skin	Adult, Juvenile	Crease	1000-10000	10000-100000	Parasitic protection
537	*Loxodonta africana*	Skin	Adult, Juvenile	Crease	1000-10000	10000-100000	Radiation protection
538	*Loxodonta africana*	Skin	Adult	Crease	1000-10000	10000-100000	Thermoregulation
539	*Loxolomia johnsoni*	Wing	Adult	Perpendicular			Acoustic decoy
540	*Macrotus californicus*	Ear	Adult	Parallel/ Curved	10-100	100-1000	Sound detection
541	*Macrotus californicus*	Ear	Adult	Parallel/ Curved	10-100	100-1000	Structural support
542	*Matricaria chamomilla*	Flower	Adult	Wavy	100-1000	0.1-1	Light reflection
543	*Melanophidium bilineatum*	Integument		Parallel		0.1-1	Structural color
544	*Melanophidium bilineatum*	Integument		Parallel		0.1-1	Particle removal
545	*Melanophidium punctatum*	Integument		Parallel		0.1-1	Structural color
546	*Melanophidium punctatum*	Integument		Parallel		0.1-1	Particle removal

	N
514	Voigt D, Gorb S. 2017 Functional morphology of tarsal adhesive pads and attachment ability in ticks Ixodes ricinus (Arachnida, Acari, Ixodidae). Journal of Ex[illegible]
515	Hackman RH. Structure and function in tick cuticle. Annual review of entomology. 1982 Jan;27(1):75-95.
516	Hackman RH. Structure and function in tick cuticle. Annual review of entomology. 1982 Jan;27(1):75-95.
517	Huang X, Hai Y, Xie W-H. 2017 Anisotropic cell growth-regulated surface micropatterns in flower petals. Theoretical and Applied Mechanics Letters 7, 169–17
518	Hsiung B-K, Blackledge TA, Shawkey MD. 2014 Structural color and its interaction with other color-producing elements: perspectives from spiders. In (eds R Li
519	Hsiung B-K, Blackledge TA, Shawkey MD. 2014 Structural color and its interaction with other color-producing elements: perspectives from spiders. In (eds R Li
520	Martinez A, Nguyen D, Basson MS, Medina J, Irschick DJ, Baeckens S. 2021 Quantifying surface topography of biological systems from 3D scans. Methods E
521	Moyroud E et al. 2017 Disorder in convergent floral nanostructures enhances signalling to bees. Nature 550, 469–474. (doi:10.1038/nature24285)
522	Neil TR, Kennedy EE, Harris BJ, Holderied MW. 2021 Wingtip folds and ripples on saturniid moths create decoy echoes against bat biosonar. Current Biology
523	Baker JR, McCrae JM. A further study of the Lepidocyrtus ("Podura") scale (Insecta, Collembola). Journal of ultrastructure research. 1967 Aug 30;19(5-6):611-
524	Moyroud E et al. 2017 Disorder in convergent floral nanostructures enhances signalling to bees. Nature 550, 469–474. (doi:10.1038/nature24285)
525	Fang Y, Sun G, Bi Y, Zhi H. 2015 Multiple-dimensional micro/nano structural models for hydrophobicity of butterfly wing surfaces and coupling mechanism. Sci
526	Fang Y, Sun G, Bi Y, Zhi H. 2015 Multiple-dimensional micro/nano structural models for hydrophobicity of butterfly wing surfaces and coupling mechanism. Sci
527	Prüm B, Seidel R, Bohn HF, Speck T. 2012 Plant surfaces with cuticular folds are slippery for beetles. J. R. Soc. Interface. 9, 127–135. (doi:10.1098/rsif.2011
528	Prüm B, Seidel R, Bohn HF, Speck T. 2012 Plant surfaces with cuticular folds are slippery for beetles. J. R. Soc. Interface. 9, 127–135. (doi:10.1098/rsif.2011
529	Neil TR, Kennedy EE, Harris BJ, Holderied MW. 2021 Wingtip folds and ripples on saturniid moths create decoy echoes against bat biosonar. Current Biology
530	Neil TR, Kennedy EE, Harris BJ, Holderied MW. 2021 Wingtip folds and ripples on saturniid moths create decoy echoes against bat biosonar. Current Biology
531	Keeley BW, Keeley ATH, Houlahan P. 2018 Ridge number in bat ears is related to both guild membership and ear length. PLoS ONE 13, e0200255. (doi:10.
532	Keeley BW, Keeley ATH, Houlahan P. 2018 Ridge number in bat ears is related to both guild membership and ear length. PLoS ONE 13, e0200255. (doi:10.
533	Keeley BW, Keeley ATH, Houlahan P. 2018 Ridge number in bat ears is related to both guild membership and ear length. PLoS ONE 13, e0200255. (doi:10.
534	Keeley BW, Keeley ATH, Houlahan P. 2018 Ridge number in bat ears is related to both guild membership and ear length. PLoS ONE 13, e0200255. (doi:10.
535	Fang Y, Sun G, Bi Y, Zhi H. 2015 Multiple-dimensional micro/nano structural models for hydrophobicity of butterfly wing surfaces and coupling mechanism. Sci
536	LILLYWHITE HB, Stein BR. 1987 Surface sculpturing and water retention of elephant skin. Journal of Zoology 211, 727–734. (doi:10.1111/j.1469-7998.1987
537	Kenagy J. 2017 Mammalogy: Adaptation, Diversity, Ecology . Fourth Edition. By George A. Feldhamer, Lee C. Drickamer, Stephen H. Vessey, Joseph F. Mer[illegible]
538	Kenagy J. 2017 Mammalogy: Adaptation, Diversity, Ecology . Fourth Edition. By George A. Feldhamer, Lee C. Drickamer, Stephen H. Vessey, Joseph F. Mer[illegible]
539	Neil TR, Kennedy EE, Harris BJ, Holderied MW. 2021 Wingtip folds and ripples on saturniid moths create decoy echoes against bat biosonar. Current Biology
540	Keeley BW, Keeley ATH, Houlahan P. 2018 Ridge number in bat ears is related to both guild membership and ear length. PLoS ONE 13, e0200255. (doi:10.
541	Keeley BW, Keeley ATH, Houlahan P. 2018 Ridge number in bat ears is related to both guild membership and ear length. PLoS ONE 13, e0200255. (doi:10.
542	Kooi CJ, Wilts BD, Leertouwer HL, Staal M, Elzenga JTM, Stavenga DG. 2014 Iridescent flowers? Contribution of surface structures to optical signaling. New [illegible]
543	Gower DJ. 2003 Scale microornamentation of uropeltid snakes. J. Morphol. 258, 249–268. (doi:10.1002/jmor.10147)
544	Gower DJ. 2003 Scale microornamentation of uropeltid snakes. J. Morphol. 258, 249–268. (doi:10.1002/jmor.10147)
545	Gower DJ. 2003 Scale microornamentation of uropeltid snakes. J. Morphol. 258, 249–268. (doi:10.1002/jmor.10147)
546	Gower DJ. 2003 Scale microornamentation of uropeltid snakes. J. Morphol. 258, 249–268. (doi:10.1002/jmor.10147)

	A	B	C	D	E	F
547	Terrestrial	Animalia	Chordata	Reptilia	Squamata	Uropeltidae
548	Terrestrial	Animalia	Chordata	Reptilia	Squamata	Uropeltidae
549	Terrestrial	Plantae	Angiosperms	Eudicots	Cornales	Loasaceae
550	Terrestrial	Plantae	Angiosperms	Eudicots	Cornales	Loasaceae
551	Terrestrial	Animalia	Chordata	Mammalia	Chiroptera	Phyllostomidae
552	Terrestrial	Animalia	Chordata	Mammalia	Chiroptera	Phyllostomidae
553	Terrestrial	Animalia	Chordata	Mammalia	Chiroptera	Phyllostomidae
554	Terrestrial	Animalia	Chordata	Mammalia	Chiroptera	Phyllostomidae
555	Terrestrial	Animalia	Chordata	Mammalia	Chiroptera	Phyllostomidae
556	Terrestrial	Animalia	Chordata	Mammalia	Chiroptera	Phyllostomidae
557	Terrestrial	Animalia	Chordata	Mammalia	Chiroptera	Phyllostomidae
558	Terrestrial	Animalia	Chordata	Mammalia	Chiroptera	Phyllostomidae
559	Terrestrial	Animalia	Chordata	Mammalia	Chiroptera	Phyllostomidae
560	Terrestrial	Animalia	Chordata	Mammalia	Chiroptera	Phyllostomidae
561	Terrestrial	Animalia	Arthropoda	Insecta	Lepidoptera	Nymphalidae
562	Terrestrial	Animalia	Chordata	Mammalia	Chiroptera	Phyllostomidae
563	Terrestrial	Animalia	Chordata	Mammalia	Chiroptera	Phyllostomidae
564	Terrestrial	Animalia	Chordata	Mammalia	Chiroptera	Phyllostomidae
565	Terrestrial	Animalia	Chordata	Mammalia	Chiroptera	Phyllostomidae
566	Terrestrial	Animalia	Arthropoda	Insecta	Lepidoptera	Nymphalidae
567	Terrestrial	Animalia	Chordata	Mammalia	Chiroptera	Vespertilionidae
568	Terrestrial	Animalia	Chordata	Mammalia	Chiroptera	Vespertilionidae
569	Terrestrial	Animalia	Chordata	Mammalia	Chiroptera	Vespertilionidae
570	Terrestrial	Animalia	Chordata	Mammalia	Chiroptera	Vespertilionidae
571	Terrestrial	Animalia	Chordata	Mammalia	Chiroptera	Vespertilionidae
572	Terrestrial	Animalia	Chordata	Mammalia	Chiroptera	Vespertilionidae
573	Terrestrial	Animalia	Chordata	Mammalia	Chiroptera	Vespertilionidae
574	Terrestrial	Animalia	Chordata	Mammalia	Chiroptera	Vespertilionidae
575	Terrestrial	Animalia	Chordata	Mammalia	Chiroptera	Vespertilionidae
576	Terrestrial	Animalia	Chordata	Mammalia	Chiroptera	Vespertilionidae
577	Terrestrial	Animalia	Chordata	Mammalia	Chiroptera	Vespertilionidae
578	Terrestrial	Animalia	Chordata	Mammalia	Chiroptera	Vespertilionidae
579	Terrestrial	Animalia	Arthropoda	Insecta	Blattodea	Blaberidae

	G	H	I	J	K	L	M
547	*Melanophidium wynaudense*	Integument		Parallel		0.1-1	Structural color
548	*Melanophidium wynaudense*	Integument		Parallel		0.1-1	Particle removal
549	*Mentzelia lindleyi*	Flower	Adult	Parallel	100-1000	0.1-1	Structural color
550	*Mentzelia lindleyi*	Flower	Adult	Parallel	100-1000		
551	*Micronycteris hirsuta*	Ear	Adult	Parallel/ Curved		100-1000	Sound detection
552	*Micronycteris hirsuta*	Ear	Adult	Parallel/ Curved		100-1000	Structural support
553	*Micronycteris megalotis*	Ear	Adult	Parallel/ Curved	10-100	100-1000	Sound detection
554	*Micronycteris megalotis*	Ear	Adult	Parallel/ Curved	10-100	100-1000	Structural support
555	*Micronycteris microtis*	Ear	Adult	Parallel/ Curved	10-100	100-1000	Sound detection
556	*Micronycteris microtis*	Ear	Adult	Parallel/ Curved	10-100	100-1000	Structural support
557	*Micronycteris minuta*	Ear	Adult	Parallel/ Curved	10-100	100-1000	Sound detection
558	*Micronycteris minuta*	Ear	Adult	Parallel/ Curved	10-100	100-1000	Structural support
559	*Micronycteris schmidtorum*	Ear	Adult	Parallel/ Curved	10-100	100-1000	Sound detection
560	*Micronycteris schmidtorum*	Ear	Adult	Parallel/ Curved	10-100	100-1000	Structural support
561	*Mimathyma nycteis*	Wing	Adult	Parallel		0.1-1	Hydrophobicity
562	*Mimon bennettii*	Ear	Adult	Parallel/ Curved	10-100	100-1000	Sound detection
563	*Mimon bennettii*	Ear	Adult	Parallel/ Curved	10-100	100-1000	Structural support
564	*Mimon crenulatum*	Ear	Adult	Parallel/ Curved	10-100	100-1000	Sound detection
565	*Mimon crenulatum*	Ear	Adult	Parallel/ Curved	10-100	100-1000	Structural support
566	*Minois dryas*	Wing	Adult	Parallel	10-100	0.1-1	Hydrophobicity
567	*Myotis auriculus*	Ear	Adult	Parallel/ Curved	10-100	100-1000	Sound detection
568	*Myotis auriculus*	Ear	Adult	Parallel/ Curved	10-100	100-1000	Structural support
569	*Myotis bechsteinii*	Ear	Adult	Parallel/ Curved	10-100	100-1000	Sound detection
570	*Myotis bechsteinii*	Ear	Adult	Parallel/ Curved	10-100	100-1000	Structural support
571	*Myotis blythii*	Ear	Adult	Parallel/ Curved	10-100	100-1000	Sound detection
572	*Myotis blythii*	Ear	Adult	Parallel/ Curved	10-100	100-1000	Structural support
573	*Myotis evotis*	Ear	Adult	Parallel/ Curved	10-100	100-1000	Sound detection
574	*Myotis evotis*	Ear	Adult	Parallel/ Curved	10-100	100-1000	Structural support
575	*Myotis myotis*	Ear	Adult	Parallel/ Curved	10-100	100-1000	Sound detection
576	*Myotis myotis*	Ear	Adult	Parallel/ Curved	10-100	100-1000	Structural support
577	*Myotis septentrionalis*	Ear	Adult	Parallel/ Curved	10-100	100-1000	Sound detection
578	*Myotis septentrionalis*	Ear	Adult	Parallel/ Curved	10-100	100-1000	Structural support
579	*Nauphoeta cinerea*	Leg	Adult	Perpendicular	10-100	0.1-1	Adhesion and friction

	N
547	Gower DJ. 2003 Scale microornamentation of uropeltid snakes. J. Morphol. 258, 249–268. (doi:10.1002/jmor.10147)
548	Gower DJ. 2003 Scale microornamentation of uropeltid snakes. J. Morphol. 258, 249–268. (doi:10.1002/jmor.10147)
549	Moyroud E et al. 2017 Disorder in convergent floral nanostructures enhances signalling to bees. Nature 550, 469–474. (doi:10.1038/nature24285)
550	Whitney HM, Bennett KMV, Dorling M, Sandbach L, Prince D, Chittka L, Glover BJ. 2011 Why do so many petals have conical epidermal cells? Annals of Bota
551	Keeley BW, Keeley ATH, Houlahan P. 2018 Ridge number in bat ears is related to both guild membership and ear length. PLoS ONE 13, e0200255. (doi:10.
552	Keeley BW, Keeley ATH, Houlahan P. 2018 Ridge number in bat ears is related to both guild membership and ear length. PLoS ONE 13, e0200255. (doi:10.
553	Keeley BW, Keeley ATH, Houlahan P. 2018 Ridge number in bat ears is related to both guild membership and ear length. PLoS ONE 13, e0200255. (doi:10.
554	Keeley BW, Keeley ATH, Houlahan P. 2018 Ridge number in bat ears is related to both guild membership and ear length. PLoS ONE 13, e0200255. (doi:10.
555	Keeley BW, Keeley ATH, Houlahan P. 2018 Ridge number in bat ears is related to both guild membership and ear length. PLoS ONE 13, e0200255. (doi:10.
556	Keeley BW, Keeley ATH, Houlahan P. 2018 Ridge number in bat ears is related to both guild membership and ear length. PLoS ONE 13, e0200255. (doi:10.
557	Keeley BW, Keeley ATH, Houlahan P. 2018 Ridge number in bat ears is related to both guild membership and ear length. PLoS ONE 13, e0200255. (doi:10.
558	Keeley BW, Keeley ATH, Houlahan P. 2018 Ridge number in bat ears is related to both guild membership and ear length. PLoS ONE 13, e0200255. (doi:10.
559	Keeley BW, Keeley ATH, Houlahan P. 2018 Ridge number in bat ears is related to both guild membership and ear length. PLoS ONE 13, e0200255. (doi:10.
560	Keeley BW, Keeley ATH, Houlahan P. 2018 Ridge number in bat ears is related to both guild membership and ear length. PLoS ONE 13, e0200255. (doi:10.
561	Fang Y, Sun G, Bi Y, Zhi H. 2015 Multiple-dimensional micro/nano structural models for hydrophobicity of butterfly wing surfaces and coupling mechanism. Sci
562	Keeley BW, Keeley ATH, Houlahan P. 2018 Ridge number in bat ears is related to both guild membership and ear length. PLoS ONE 13, e0200255. (doi:10.
563	Keeley BW, Keeley ATH, Houlahan P. 2018 Ridge number in bat ears is related to both guild membership and ear length. PLoS ONE 13, e0200255. (doi:10.
564	Keeley BW, Keeley ATH, Houlahan P. 2018 Ridge number in bat ears is related to both guild membership and ear length. PLoS ONE 13, e0200255. (doi:10.
565	Keeley BW, Keeley ATH, Houlahan P. 2018 Ridge number in bat ears is related to both guild membership and ear length. PLoS ONE 13, e0200255. (doi:10.
566	Fang Y, Sun G, Bi Y, Zhi H. 2015 Multiple-dimensional micro/nano structural models for hydrophobicity of butterfly wing surfaces and coupling mechanism. Sci
567	Keeley BW, Keeley ATH, Houlahan P. 2018 Ridge number in bat ears is related to both guild membership and ear length. PLoS ONE 13, e0200255. (doi:10.
568	Keeley BW, Keeley ATH, Houlahan P. 2018 Ridge number in bat ears is related to both guild membership and ear length. PLoS ONE 13, e0200255. (doi:10.
569	Keeley BW, Keeley ATH, Houlahan P. 2018 Ridge number in bat ears is related to both guild membership and ear length. PLoS ONE 13, e0200255. (doi:10.
570	Keeley BW, Keeley ATH, Houlahan P. 2018 Ridge number in bat ears is related to both guild membership and ear length. PLoS ONE 13, e0200255. (doi:10.
571	Keeley BW, Keeley ATH, Houlahan P. 2018 Ridge number in bat ears is related to both guild membership and ear length. PLoS ONE 13, e0200255. (doi:10.
572	Keeley BW, Keeley ATH, Houlahan P. 2018 Ridge number in bat ears is related to both guild membership and ear length. PLoS ONE 13, e0200255. (doi:10.
573	Keeley BW, Keeley ATH, Houlahan P. 2018 Ridge number in bat ears is related to both guild membership and ear length. PLoS ONE 13, e0200255. (doi:10.
574	Keeley BW, Keeley ATH, Houlahan P. 2018 Ridge number in bat ears is related to both guild membership and ear length. PLoS ONE 13, e0200255. (doi:10.
575	Keeley BW, Keeley ATH, Houlahan P. 2018 Ridge number in bat ears is related to both guild membership and ear length. PLoS ONE 13, e0200255. (doi:10.
576	Keeley BW, Keeley ATH, Houlahan P. 2018 Ridge number in bat ears is related to both guild membership and ear length. PLoS ONE 13, e0200255. (doi:10.
577	Keeley BW, Keeley ATH, Houlahan P. 2018 Ridge number in bat ears is related to both guild membership and ear length. PLoS ONE 13, e0200255. (doi:10.
578	Keeley BW, Keeley ATH, Houlahan P. 2018 Ridge number in bat ears is related to both guild membership and ear length. PLoS ONE 13, e0200255. (doi:10.
579	Clemente CJ, Dirks J-H, Barbero DR, Steiner U, Federle W. 2009 Friction ridges in cockroach climbing pads: anisotropy of shear stress measured on transpare

	A	B	C	D	E	F
580	Terrestrial	Animalia	Arthropoda	Insecta	Blattodea	Blaberidae
581	Terrestrial	Fungi	Ascomycota	Saccharomycetes	Saccharomycetales	Saccharomycetaceae
582	Terrestrial	Animalia	Nematoda	Secernentea	Tylenchida	Telotylenchidae
583	Terrestrial	Plantae	Angiosperms	Eudicots	Caryophyllales	Nepenthaceae
584	Terrestrial	Plantae	Angiosperms	Eudicots	Caryophyllales	Nepenthaceae
585	Terrestrial	Animalia	Arthropoda	Insecta	Lepidoptera	Nymphalidae
586	Terrestrial	Animalia	Arthropoda	Insecta	Lepidoptera	Nymphalidae
587	Terrestrial	Animalia	Arthropoda	Insecta	Lepidoptera	Saturniidae
588	Terrestrial	Animalia	Arthropoda	Insecta	Lepidoptera	Psychoidea
589	Terrestrial	Plantae	Angiosperms	Eudicots	Myrtales	Onagraceae
590	Terrestrial	Animalia	Nematoda	Chromadorea	Strongylida	Strongylidae
591	Terrestrial	Animalia	Nematoda	Chromadorea	Rhabditida	Onchocercidae
592	Terrestrial	Animalia	Arthropoda	Arachnida	Ixodida	Argasidae
593	Terrestrial	Plantae	Angiosperms	Monocots	Poales	Poaceae
594	Terrestrial	Animalia	Chordata	Mammalia	Chiroptera	Vespertilionidae
595	Terrestrial	Animalia	Chordata	Mammalia	Chiroptera	Vespertilionidae
596	Terrestrial	Plantae	Angiosperms	Eudicots	Saxifragales	Paeoniaceae
597	Terrestrial	Plantae	Angiosperms	Eudicots	Apiales	Araliaceae
598	Terrestrial	Animalia	Arthropoda	Insecta	Lepidoptera	Papilionidae
599	Terrestrial	Animalia	Arthropoda	Insecta	Lepidoptera	Papilionidae
600	Terrestrial	Animalia	Platyhelminthes	Trematoda	Plagiorchiida	Paramphistomatidae
601	Terrestrial	Animalia	Arthropoda	Insecta	Lepidoptera	Papilionidae
602	Terrestrial	Animalia	Chordata	Aves	Bucerotiformes	Bucerotidae
603	Terrestrial	Plantae	Angiosperms	Eudicots	Lamiales	Plantaginaceae
604	Terrestrial	Animalia	Arthropoda	Insecta	Coleoptera	Staphylinidae
605	Terrestrial	Animalia	Chordata	Mammalia	Chiroptera	Phyllostomidae
606	Terrestrial	Animalia	Chordata	Mammalia	Chiroptera	Phyllostomidae
607	Terrestrial	Animalia	Chordata	Mammalia	Chiroptera	Phyllostomidae
608	Terrestrial	Animalia	Chordata	Mammalia	Chiroptera	Phyllostomidae
609	Terrestrial	Animalia	Chordata	Reptilia	Squamata	Colubridae
610	Terrestrial	Animalia	Chordata	Mammalia	Chiroptera	Vespertilionidae
611	Terrestrial	Animalia	Chordata	Mammalia	Chiroptera	Vespertilionidae

	G	H	I	J	K	L	M
580	*Nauphoeta cinerea*	Leg	Adult	Perpendicular	10-100	0.1-1	Interlocking
581	*Nematospora (Eremothecium*	Integument		Perpendicular	0.01-0.1	0.1-1	Attachment
582	*Neodolichorhynchus n.g.*	Skin	Adult	Parallel			
583	*Nepenthes bicalcarata*	Peristome	Adult	Parallel	1000-10000	100-1000	Insect aquaplaning
584	*Nepenthes x mixta*	Peristome	Adult	Parallel	100-1000	100-1000	Chanelling
585	*Neptis philyroides*	Wing	Adult	Parallel		0.1-1	Hydrophobicity
586	*Neptis rivularis*	Wing	Adult	Parallel	10-100	0.1-1	Hydrophobicity
587	*Nudaurelia dione*	Wing	Adult	Perpendicular			Acoustic decoy
588	*Oeceticus omnivorus*	Antenna	Adult	Parallel and Labyrinth	10-100		
589	*Oenothera stricta*	Flower	Adult	Parallel	100-1000	0.1-1	Structural color
590	*Oesophagostomum dentatu*	Skin	Juvenile	Perpendicular	0.1-1	0.1-1	
591	*Onchocerca volvulus*	Skin	Adult	Parallel	100-1000	0.1-1	
592	*Ornithodoros savignyi*	Skin	Larva	Labyrinth	1-10	1-10	
593	*Oryza sativa L.*	Flower	Adult	Labyrlnth	1000-10000	1-10	Structural support
594	*Otonycteris hemprichi*	Ear	Adult	Parallel/ Curved	10-100	100-1000	Sound detection
595	*Otonycteris hemprichi*	Ear	Adult	Parallel/ Curved	10-100	100-1000	Structural support
596	*Paeonia mascula*	Flower	Adult	Parallel	1000-10000	1-10	Structural color
597	*Panax ginseng*	Leaf	Adult	Labyrinth	100-1000	0.1-1	Thermoregulation
598	*Papilio bianor*	Wing	Adult	Parallel	10-100	0.1-1	Hydrophobicity
599	*Papilio xuthus*	Wing	Adult	Parallel	10-100	0.1-1	Hydrophobicity
600	*Paramphistomum epklitu*	Skin		Perpendicular	1-10	10-100	Attachment
601	*Parnassius glacialis*	Wing	Adult	Parallel	10-100	0.1-1	Hydrophobicity
602	*Penelopides panini panini*	Head	Ontogeny	Perpendicular	100-1000	1000-10000	Sexual ornamentation
603	*Penstemon barrettiae*	Flower	Adult	Parallel	100-1000	0.1-1	Structural color
604	*Philonthus sericans*	Egg		Wavy	0.1-1	1-10	Structural support
605	*Phylloderma stenops*	Ear	Adult	Parallel/ Curved	100-1000	100-1000	Sound detection
606	*Phylloderma stenops*	Ear	Adult	Parallel/ Curved	100-1000	100-1000	Structural support
607	*Phyllostomus elongatus*	Ear	Adult	Parallel/ Curved	10-100	100-1000	Sound detection
608	*Phyllostomus elongatus*	Ear	Adult	Parallel/ Curved	10-100	100-1000	Structural support
609	*Pituophis catenifer*	Skin	Adult	Parallel	1000-10000	10-100	Friction and Wear reduction
610	*Plecotus alpinus*	Ear	Adult	Parallel/ Curved	10-100	100-1000	Sound detection
611	*Plecotus alpinus*	Ear	Adult	Parallel/ Curved	10-100	100-1000	Structural support

	N
580	Clemente CJ, Dirks J-H, Barbero DR, Steiner U, Federle W. 2009 Friction ridges in cockroach climbing pads: anisotropy of shear stress measured on transpare
581	cHolley RA, Allan-Wojtas P, Phipps-Todd BE. 1984 Nematospora sinecauda sp. nov., a yeast pathogen of mustard seeds. Antonie van Leeuwenhoek 50, 30
582	Jairajpuri MS, Hunt DJ. 1984 The taxonomy of Tylenchorhynchinae (Nematoda: Tylenchida) with longitudinal lines and ridges. Syst Parasitol 6, 261–268. (doi:
583	Bohn HF, Federle W. 2004 Insect aquaplaning: Nepenthes pitcher plants capture prey with the peristome, a fully wettable water-lubricated anisotropic surface
584	Box F, Thorogood C, Hui Guan J. 2019 Guided droplet transport on synthetic slippery surfaces inspired by a pitcher plant. Journal of The Royal Society Interfa
585	Fang Y, Sun G, Bi Y, Zhi H. 2015 Multiple-dimensional micro/nano structural models for hydrophobicity of butterfly wing surfaces and coupling mechanism. Sci
586	Fang Y, Sun G, Bi Y, Zhi H. 2015 Multiple-dimensional micro/nano structural models for hydrophobicity of butterfly wing surfaces and coupling mechanism. Sci
587	Neil TR, Kennedy EE, Harris BJ, Holderied MW. 2021 Wingtip folds and ripples on saturniid moths create decoy echoes against bat biosonar. Current Biology
588	Flower NE, Helson GAH. 1977 The structure of sensors on the antennae of Oeceticus omnivorus (Lepidoptera: Psychidae). New Zealand Journal of Zoology 4
589	Moyroud E et al. 2017 Disorder in convergent floral nanostructures enhances signalling to bees. Nature 550, 469–474. (doi:10.1038/nature24285)
590	Neuhaus B, Bresciani J, Christensen CM, Frandsen F. Ultrastructure and development of the body cuticle of Oesophagostomum dentatum (Strongylida, Nema
591	Lustigman S, Huima T, Brotman B, Miller K, Prince AM. 1990 Onchocerca volvulus: Biochemical and morphological characteristics of the surface of third- and f
592	Abdel-Shafy S, Gabr HSM, Abdullah HHAM, Mahmoud MS. 2016 Morphological and molecular description of immature stages of Ornithodoros savignyi (Acari:
593	Takeoka, Yoji, et al. "Scanning electron microscopic observations on morphogenesis of the panicle and spikelet in rice plants." Japanese Journal of Crop Scie
594	Keeley BW, Keeley ATH, Houlahan P. 2018 Ridge number in bat ears is related to both guild membership and ear length. PLoS ONE 13, e0200255. (doi:10.
595	Keeley BW, Keeley ATH, Houlahan P. 2018 Ridge number in bat ears is related to both guild membership and ear length. PLoS ONE 13, e0200255. (doi:10.
596	Moyroud E et al. 2017 Disorder in convergent floral nanostructures enhances signalling to bees. Nature 550, 469–474. (doi:10.1038/nature24285)
597	Lee K, Nah S-Y, Kim E-S. 2015 Micromorphology and development of the epicuticular structure on the epidermal cell of ginseng leaves. Journal of Ginseng R
598	Fang Y, Sun G, Bi Y, Zhi H. 2015 Multiple-dimensional micro/nano structural models for hydrophobicity of butterfly wing surfaces and coupling mechanism. Sci
599	Fang Y, Sun G, Bi Y, Zhi H. 2015 Multiple-dimensional micro/nano structural models for hydrophobicity of butterfly wing surfaces and coupling mechanism. Sci
600	Tandon V, Maitra SC. Stereocan observations on the surface topography of Gastrothylax crumenifer (Creplin, 1847) Poirier, 1883 and Paramphistomum epicli
601	Fang Y, Sun G, Bi Y, Zhi H. 2015 Multiple-dimensional micro/nano structural models for hydrophobicity of butterfly wing surfaces and coupling mechanism. Sci
602	Curio E. 2004 On ornamental maturation of two Philippine hornbill species with a note on physiological colour change. J Ornithol 145. (doi:10.1007/s10336-0(
603	Moyroud E et al. 2017 Disorder in convergent floral nanostructures enhances signalling to bees. Nature 550, 469–474. (doi:10.1038/nature24285)
604	Hu, G. Y., and J. H. Frank. "Structural comparison of the chorion surface of five Philonthus species (Coleoptera: Staphylinidae)." Entomological Society of Wa
605	Keeley BW, Keeley ATH, Houlahan P. 2018 Ridge number in bat ears is related to both guild membership and ear length. PLoS ONE 13, e0200255. (doi:10.
606	Keeley BW, Keeley ATH, Houlahan P. 2018 Ridge number in bat ears is related to both guild membership and ear length. PLoS ONE 13, e0200255. (doi:10.
607	Keeley BW, Keeley ATH, Houlahan P. 2018 Ridge number in bat ears is related to both guild membership and ear length. PLoS ONE 13, e0200255. (doi:10.
608	Keeley BW, Keeley ATH, Houlahan P. 2018 Ridge number in bat ears is related to both guild membership and ear length. PLoS ONE 13, e0200255. (doi:10.
609	Martinez A, Nguyen D, Basson MS, Medina J, Irschick DJ, Baeckens S. 2021 Quantifying surface topography of biological systems from 3D scans. Methods E
610	Keeley BW, Keeley ATH, Houlahan P. 2018 Ridge number in bat ears is related to both guild membership and ear length. PLoS ONE 13, e0200255. (doi:10.
611	Keeley BW, Keeley ATH, Houlahan P. 2018 Ridge number in bat ears is related to both guild membership and ear length. PLoS ONE 13, e0200255. (doi:10.

	A	B	C	D	E	F
612	Terrestrial	Animalia	Chordata	Mammalia	Chiroptera	Vespertilionidae
613	Terrestrial	Animalia	Chordata	Mammalia	Chiroptera	Vespertilionidae
614	Terrestrial	Animalia	Chordata	Mammalia	Chiroptera	Vespertilionidae
615	Terrestrial	Animalia	Chordata	Mammalia	Chiroptera	Vespertilionidae
616	Terrestrial	Animalia	Chordata	Reptilia	Squamata	Uropeltidae
617	Terrestrial	Animalia	Chordata	Reptilia	Squamata	Uropeltidae
618	Terrestrial	Animalia	Arthropoda	Insecta	Lepidoptera	Nymphalidae
619	Terrestrial	Animalia	Arthropoda	Insecta	Lepidoptera	Nymphalidae
620	Terrestrial	Animalia	Arthropoda	Insecta	Lepidoptera	Pieridae
621	Terrestrial	Animalia	Arthropoda	Crustacea	Isopoda	Porcellionidae
622	Terrestrial	Animalia	Arthropoda	Crustacea	Isopoda	Porcellionidae
623	Terrestrial	Animalia	Arthropoda	Insecta	Lepidoptera	Saturniidae
624	Terrestrial	Animalia	Arthropoda	Insecta	Lepidoptera	Saturniidae
625	Terrestrial	Animalia	Chordata	Reptilia	Squamata	Uropeltidae
626	Terrestrial	Animalia	Chordata	Reptilia	Squamata	Uropeltidae
627	Terrestrial	Animalia	Arthropoda	Insecta	Lepidoptera	Saturniidae
628	Terrestrial	Animalia	Chordata	Reptilia	Squamata	Uropeltidae
629	Terrestrial	Animalia	Chordata	Reptilia	Squamata	Uropeltidae
630	Terrestrial	Plantae	Angiosperms	Eudicots	Ericales	Ericaceae
631	Terrestrial	Chromista	Apicomplexa	Conoidasida	Eugregarinida	
632	Terrestrial	Animalia	Arthropoda	Insecta	Lepidoptera	Saturniidae
633	Terrestrial	Animalia	Arthropoda	Insecta	Lepidoptera	Saturniidae
634	Terrestrial	Animalia	Arthropoda	Insecta	Lepidoptera	Saturniidae
635	Terrestrial	Plantae	Angiosperms	Eudicots	Asterales	Asteraceae
636	Terrestrial	Animalia	Arthropoda	Insecta	Lepidoptera	Saturniidae
637	Terrestrial	Animalia	Arthropoda	Insecta	Lepidoptera	Saturniidae
638	Terrestrial	Animalia	Arthropoda	Insecta	Lepidoptera	Saturniidae
639	Terrestrial	Animalia	Arthropoda	Insecta	Lepidoptera	Saturniidae
640	Terrestrial	Plantae	Angiosperms	Monocots	Alismatales	Araceae
641	Terrestrial	Plantae	Angiosperms	Monocots	Alismatales	Araceae
642	Terrestrial	Plantae	Angiosperms	Monocots	Alismatales	Araceae
643	Terrestrial	Animalia	Platyhelminthes	Trematoda	Diplostomida	Schistosomatidae

	G	H	I	J	K	L	M
612	*Plecotus auritus*	Ear	Adult	Parallel/ Curved	10-100	100-1000	Sound detection
613	*Plecotus auritus*	Ear	Adult	Parallel/ Curved	10-100	100-1000	Structural support
614	*Plecotus austriacus*	Ear	Adult	Parallel/ Curved	10-100	100-1000	Sound detection
615	*Plecotus austriacus*	Ear	Adult	Parallel/ Curved	10-100	100-1000	Structural support
616	*Plectrurus perrotetii*	Integument		Parallel	100-1000	0.1-1	Structural color
617	*Plectrurus perrotetii*	Integument		Parallel	100-1000	0.1-1	Particle removal
618	*Polygonia c-album*	Wing	Adult	Parallel	10-100	0.1-1	Hydrophobicity
619	*Polygonia c-aureum*	Wing	Adult	Parallel	10-100	0.1-1	Hydrophobicity
620	*Pontia daplidice*	Wing	Adult	Parallel	10-100	0.1-1	Hydrophobicity
621	*Porcellio scaber*	Leg	Adult	Parallel	10-100	0.1-1	Friction and Wear reduction
622	*Porcellio scaber*	Leg	Adult	Parallel	10-100	0.1-1	Particle removal
623	*Pseudimbrasia deyrollei*	Wing	Adult	Perpendicular			Acoustic decoy
624	*Pseudobunaea epithyrena*	Wing	Adult	Perpendicular			Acoustic decoy
625	*Pseudotyphlops philippinus*	Integument		Parallel	10-100	0.1-1	Structural color
626	*Pseudotyphlops philippinus*	Integument		Parallel	10-100	0.1-1	Particle removal
627	*Rhescyntis hippodamia*	Wing	Adult	Perpendicular			Acoustic decoy
628	*Rhinophis blythii*	Integument		Parallel	100-1000	0.1-1	Structural color
629	*Rhinophis blythii*	Integument		Parallel	100-1000	0.1-1	Particle removal
630	*Rhododendron yunnanense*	Flower	Adult	Perpendicular	1000-10000	1-10	
631	*Rhynchocystis pilosa*	Skin	Adult	Parallel	0.1-1	1-10	Attachment
632	*Rothschildia aurota*	Wing	Adult	Perpendicular			Acoustic decoy
633	*Rothschildia erycina*	Wing	Adult	Perpendicular			Acoustic decoy
634	*Rothschildia lebeau*	Wing	Adult	Perpendicular			Acoustic decoy
635	*Rudbeckia fulgida*	Flower	Adult	Parallel on sides<br>Labyrinth on apex	1000-10000	0.1-1	Light absorption
636	*Salassa lola*	Wing	Adult	Perpendicular			Acoustic decoy
637	*Samia cynthia*	Wing	Adult	Perpendicular	100-1000	100-1000	Acoustic decoy
638	*Saturnia homogena*	Wing	Adult	Perpendicular			Acoustic decoy
639	*Saturnia japonica*	Wing	Adult	Perpendicular			Acoustic decoy
640	*Schismatoglottis calyptrata*	Leaf	Adult	Labyrinth	100-1000	0.1-1	Friction and Wear reduction
641	*Schismatoglottis calyptrata*	Leaf	Adult	Labyrinth	100-1000	0.1-1	Insect adhesion
642	*Schismatoglottis calyptrata*	Leaf	Adult	Labyrinth	100-1000	0.1-1	Structural support
643	*Schistosoma japonicum*	Skin	Adult	Labyrinth	10-100	1-10	

	N
612	Keeley BW, Keeley ATH, Houlahan P. 2018 Ridge number in bat ears is related to both guild membership and ear length. PLoS ONE 13, e0200255. (doi:10.
613	Keeley BW, Keeley ATH, Houlahan P. 2018 Ridge number in bat ears is related to both guild membership and ear length. PLoS ONE 13, e0200255. (doi:10.
614	Keeley BW, Keeley ATH, Houlahan P. 2018 Ridge number in bat ears is related to both guild membership and ear length. PLoS ONE 13, e0200255. (doi:10.
615	Keeley BW, Keeley ATH, Houlahan P. 2018 Ridge number in bat ears is related to both guild membership and ear length. PLoS ONE 13, e0200255. (doi:10.
616	Gower DJ. 2003 Scale microornamentation of uropeltid snakes. J. Morphol. 258, 249–268. (doi:10.1002/jmor.10147)
617	Gower DJ. 2003 Scale microornamentation of uropeltid snakes. J. Morphol. 258, 249–268. (doi:10.1002/jmor.10147)
618	Fang Y, Sun G, Bi Y, Zhi H. 2015 Multiple-dimensional micro/nano structural models for hydrophobicity of butterfly wing surfaces and coupling mechanism. Sci
619	Fang Y, Sun G, Bi Y, Zhi H. 2015 Multiple-dimensional micro/nano structural models for hydrophobicity of butterfly wing surfaces and coupling mechanism. Sci
620	Fang Y, Sun G, Bi Y, Zhi H. 2015 Multiple-dimensional micro/nano structural models for hydrophobicity of butterfly wing surfaces and coupling mechanism. Sci
621	Vittori M. 2021 Structure of a hinge joint with textured sliding surfaces in terrestrial isopods (Crustacea: Isopoda: Oniscidea). Zoological Lett 7, 7. (doi:10.1186
622	Vittori M. 2021 Structure of a hinge joint with textured sliding surfaces in terrestrial isopods (Crustacea: Isopoda: Oniscidea). Zoological Lett 7, 7. (doi:10.1186
623	Neil TR, Kennedy EE, Harris BJ, Holderied MW. 2021 Wingtip folds and ripples on saturniid moths create decoy echoes against bat biosonar. Current Biology
624	Neil TR, Kennedy EE, Harris BJ, Holderied MW. 2021 Wingtip folds and ripples on saturniid moths create decoy echoes against bat biosonar. Current Biology
625	Gower DJ. 2003 Scale microornamentation of uropeltid snakes. J. Morphol. 258, 249–268. (doi:10.1002/jmor.10147)
626	Gower DJ. 2003 Scale microornamentation of uropeltid snakes. J. Morphol. 258, 249–268. (doi:10.1002/jmor.10147)
627	Neil TR, Kennedy EE, Harris BJ, Holderied MW. 2021 Wingtip folds and ripples on saturniid moths create decoy echoes against bat biosonar. Current Biology
628	Gower DJ. 2003 Scale microornamentation of uropeltid snakes. J. Morphol. 258, 249–268. (doi:10.1002/jmor.10147)
629	Gower DJ. 2003 Scale microornamentation of uropeltid snakes. J. Morphol. 258, 249–268. (doi:10.1002/jmor.10147)
630	Antoniou Kourounioti RL et al. 2013 Buckling as an origin of ordered cuticular patterns in flower petals. Journal of The Royal Society Interface 10, 20120847.
631	WARNER FD. The fine structure of Rhynchocystis pilosa (Sporozoa, Eugregarinida). The Journal of Protozoology. 1968 Feb;15(1):59-73.
632	Neil TR, Kennedy EE, Harris BJ, Holderied MW. 2021 Wingtip folds and ripples on saturniid moths create decoy echoes against bat biosonar. Current Biology
633	Neil TR, Kennedy EE, Harris BJ, Holderied MW. 2021 Wingtip folds and ripples on saturniid moths create decoy echoes against bat biosonar. Current Biology
634	Neil TR, Kennedy EE, Harris BJ, Holderied MW. 2021 Wingtip folds and ripples on saturniid moths create decoy echoes against bat biosonar. Current Biology
635	Schulte AJ, Mail M, Hahn LA, Barthlott W. 2019 Ultraviolet patterns of flowers revealed in polymer replica – caused by surface architecture. Beilstein J. Nanote
636	Neil TR, Kennedy EE, Harris BJ, Holderied MW. 2021 Wingtip folds and ripples on saturniid moths create decoy echoes against bat biosonar. Current Biology
637	Neil TR, Kennedy EE, Harris BJ, Holderied MW. 2021 Wingtip folds and ripples on saturniid moths create decoy echoes against bat biosonar. Current Biology
638	Neil TR, Kennedy EE, Harris BJ, Holderied MW. 2021 Wingtip folds and ripples on saturniid moths create decoy echoes against bat biosonar. Current Biology
639	Neil TR, Kennedy EE, Harris BJ, Holderied MW. 2021 Wingtip folds and ripples on saturniid moths create decoy echoes against bat biosonar. Current Biology
640	Surapaneni, V. A., Aust, T., Speck, T., & Thielen, M. (2021). Polarity in cuticular ridge development and insect attachment on leaf surfaces of Schismatoglottis
641	Surapaneni, V. A., Aust, T., Speck, T., & Thielen, M. (2021). Polarity in cuticular ridge development and insect attachment on leaf surfaces of Schismatoglottis
642	Surapaneni, V. A., Aust, T., Speck, T., & Thielen, M. (2021). Polarity in cuticular ridge development and insect attachment on leaf surfaces of Schismatoglottis
643	Sakamoto K, Ishii Y. Scanning electron microscope observations on adult Schistosoma japonicum. J Parasitol. 1977 Jun;63(3):407-12

	A	B	C	D	E	F
644	Terrestrial	Animalia	Arthropoda	Insecta	Lepidoptera	Nymphalidae
645	Terrestrial	Animalia	Arthropoda	Insecta	Diptera	Anisopodidae
646	Terrestrial	Animalia	Arthropoda	Insecta	Lepidoptera	Saturniidae
647	Terrestrial	Animalia	Arthropoda	Insecta	Diptera	Tabanidae
648	Terrestrial	Animalia	Chordata	Mammalia	Chiroptera	Phyllostomidae
649	Terrestrial	Animalia	Chordata	Mammalia	Chiroptera	Phyllostomidae
650	Terrestrial	Animalia	Chordata	Mammalia	Chiroptera	Phyllostomidae
651	Terrestrial	Animalia	Chordata	Mammalia	Chiroptera	Phyllostomidae
652	Terrestrial	Animalia	Chordata	Mammalia	Chiroptera	Phyllostomidae
653	Terrestrial	Animalia	Chordata	Mammalia	Chiroptera	Phyllostomidae
654	Terrestrial	Animalia	Nematoda	Enoplea	Trichocephalida	Trichinellidae
655	Terrestrial	Animalia	Nematoda	Enoplea	Trichocephalida	Trichinellidae
656	Terrestrial	Animalia	Nematoda	Enoplea	Trichocephalida	Trichinellidae
657	Terrestrial	Animalia	Nematoda	Secernentea	Tylenchida	Tylenchorhynchinae
658	Terrestrial	Plantae	Angiosperms	Eudicots	Austrobaileyales	Trimeniaceae
659	Terrestrial	Animalia	Chordata	Mammalia	Chiroptera	Phyllostomidae
660	Terrestrial	Animalia	Chordata	Mammalia	Chiroptera	Phyllostomidae
661	Terrestrial	Plantae	Angiosperms	Monocots	Liliales	Liliaceae
662	Terrestrial	Plantae	Angiosperms	Monocots	Liliales	Liliaceae
663	Terrestrial	Plantae	Angiosperms	Monocots	Poales	Poaceae
664	Terrestrial	Plantae	Angiosperms	Monocots	Poales	Poaceae
665	Terrestrial	Plantae	Angiosperms	Monocots	Poales	Poaceae
666	Terrestrial	Animalia	Chordata	Reptilia	Squamata	Uropeltidae
667	Terrestrial	Animalia	Chordata	Reptilia	Squamata	Uropeltidae
668	Terrestrial	Animalia	Chordata	Reptilia	Squamata	Uropeltidae
669	Terrestrial	Animalia	Chordata	Reptilia	Squamata	Uropeltidae
670	Terrestrial	Animalia	Chordata	Reptilia	Squamata	Uropeltidae
671	Terrestrial	Animalia	Chordata	Reptilia	Squamata	Uropeltidae
672	Terrestrial	Plantae	Angiosperms	Eudicots	Asterales	Asteraceae
673	Terrestrial	Plantae	Angiosperms	Eudicots	Asterales	Asteraceae
674	Terrestrial	Animalia	Chordata	Mammalia	Chiroptera	Phyllostomidae
675	Terrestrial	Animalia	Chordata	Mammalia	Chiroptera	Phyllostomidae
676	Terrestrial	Animalia	Arthropoda	Insecta	Lepidoptera	Nymphalidae

	G	H	I	J	K	L	M
644	*Speyeria aglaja*	Wing	Adult	Parallel	10-100	0.1-1	Hydrophobicity
645	*Sylvicola fenestralis*	Eye	Adult	Labyrinth	1-10	0.1-1	
646	*Syntherata janetta*	Wing	Adult	Perpendicular			Acoustic decoy
647	*Tabanus punctifer*	Eye	Adult	Labyrinth	10-100	0.1-1	
648	*Tonatia bidens*	Ear	Adult	Parallel/ Curved	10-100	100-1000	Sound detection
649	*Tonatia bidens*	Ear	Adult	Parallel/ Curved	10-100	100-1000	Structural support
650	*Tonatia saurophila*	Ear	Adult	Parallel/ Curved	10-100	100-1000	Sound detection
651	*Tonatia saurophila*	Ear	Adult	Parallel/ Curved	10-100	100-1000	Structural support
652	*Trachops cirrhosus*	Ear	Adult	Parallel/ Curved	10-100	100-1000	Sound detection
653	*Trachops cirrhosus*	Ear	Adult	Parallel/ Curved	10-100	100-1000	Structural support
654	*Trichinella spiralis nativa*	Skin	Adult	Parallel	1-10	0.01-0.1	
655	*Trichinella spiralis pseudospi*	Skin	Adult	Parallel	1-10	0.01-0.1	
656	*Trichinella spiralis spiralis*	Skin	Adult	Parallel	1-10	0.01-0.1	
657	*Trilineellus clathrocutis*	Skin	Adult	Parallel	1-10		
658	*Trimenia moorei*	Flower	Adult	Parallel			
659	*Trinycteris nicefori*	Ear	Adult	Parallel/ Curved	10-100	100-1000	Sound detection
660	*Trinycteris nicefori*	Ear	Adult	Parallel/ Curved	10-100	100-1000	Structural support
661	*Tulipa 'Queen Of Night'*	Flower	Adult	Parallel	100-1000	0.1-1	Structural color
662	*Tulipa ‘Queen of the Night’*	Flower	Adult	Parallel	100-1000	0.1-1	Structural color
663	*Urochondra setulosa*	Leaf	Adult	Parallel	100-1000	100-1000	Radiation protection
664	*Urochondra setulosa*	Leaf	Adult	Parallel	100-1000	100-1000	Structural support
665	*Urochondra setulosa*	Leaf	Adult	Parallel	100-1000	100-1000	Transpiration control
666	*Uropeltis ceylanica*	Integument		Parallel	100-1000	0.1-1	Structural color
667	*Uropeltis ceylanica*	Integument		Parallel		0.1-1	Particle removal
668	*Uropeltis melanogaster*	Integument		Parallel	100-1000	0.1-1	Structural color
669	*Uropeltis melanogaster*	Integument		Parallel	100-1000	0.1-1	Particle removal
670	*Uropeltis phillipsi*	Integument		Parallel		0.1-1	Structural color
671	*Uropeltis phillipsi*	Integument		Parallel		0.1-1	Particle removal
672	*Ursinia calendulifolia*	Flower	Adult	Perpendicular	100-1000	0.1-1	
673	*Ursinia speciosa*	Flower	Adult	Parallel	100-1000	0.1-1	Structural color
674	*Vampyrum spectrum*	Ear	Adult	Parallel/ Curved	100-1000	100-1000	Sound detection
675	*Vampyrum spectrum*	Ear	Adult	Parallel/ Curved	100-1000	100-1000	Structural support
676	*Vanessa cardui*	Wing	Adult	Parallel	10-100	0.1-1	Hydrophobicity

	N
644	Fang Y, Sun G, Bi Y, Zhi H. 2015 Multiple-dimensional micro/nano structural models for hydrophobicity of butterfly wing surfaces and coupling mechanism. Sci
645	Anderson MS, Gaimari SD. 2003 Raman-atomic force microscopy of the ommatidial surfaces of Dipteran compound eyes. Journal of Structural Biology 142, 3[illegible]
646	Neil TR, Kennedy EE, Harris BJ, Holderied MW. 2021 Wingtip folds and ripples on saturniid moths create decoy echoes against bat biosonar. Current Biology
647	Anderson MS, Gaimari SD. 2003 Raman-atomic force microscopy of the ommatidial surfaces of Dipteran compound eyes. Journal of Structural Biology 142, 3[illegible]
648	Keeley BW, Keeley ATH, Houlahan P. 2018 Ridge number in bat ears is related to both guild membership and ear length. PLoS ONE 13, e0200255. (doi:10.
649	Keeley BW, Keeley ATH, Houlahan P. 2018 Ridge number in bat ears is related to both guild membership and ear length. PLoS ONE 13, e0200255. (doi:10.
650	Keeley BW, Keeley ATH, Houlahan P. 2018 Ridge number in bat ears is related to both guild membership and ear length. PLoS ONE 13, e0200255. (doi:10.
651	Keeley BW, Keeley ATH, Houlahan P. 2018 Ridge number in bat ears is related to both guild membership and ear length. PLoS ONE 13, e0200255. (doi:10.
652	Keeley BW, Keeley ATH, Houlahan P. 2018 Ridge number in bat ears is related to both guild membership and ear length. PLoS ONE 13, e0200255. (doi:10.
653	Keeley BW, Keeley ATH, Houlahan P. 2018 Ridge number in bat ears is related to both guild membership and ear length. PLoS ONE 13, e0200255. (doi:10.
654	Lichtenfels JR, Murrell KD, Pilitt PA. Comparison of three subspecies of Trichinella spiralis by scanning electron microscopy. J Parasitol. 1983 Dec;69(6):1131-
655	Lichtenfels JR, Murrell KD, Pilitt PA. Comparison of three subspecies of Trichinella spiralis by scanning electron microscopy. J Parasitol. 1983 Dec;69(6):1131-
656	Lichtenfels JR, Murrell KD, Pilitt PA. Comparison of three subspecies of Trichinella spiralis by scanning electron microscopy. J Parasitol. 1983 Dec;69(6):1131-
657	Jairajpuri MS. 1984 Some studies on Tylenchorhynchinae: proposal ofDivittus n.g. (Nematoda: Tylenchida). Syst Parasitol 6, 107–112. (doi:10.1007/BF0218[illegible]
658	Moyroud E et al. 2017 Disorder in convergent floral nanostructures enhances signalling to bees. Nature 550, 469–474. (doi:10.1038/nature24285)
659	Keeley BW, Keeley ATH, Houlahan P. 2018 Ridge number in bat ears is related to both guild membership and ear length. PLoS ONE 13, e0200255. (doi:10.
660	Keeley BW, Keeley ATH, Houlahan P. 2018 Ridge number in bat ears is related to both guild membership and ear length. PLoS ONE 13, e0200255. (doi:10.
661	Antoniou Kourounioti RL et al. 2013 Buckling as an origin of ordered cuticular patterns in flower petals. Journal of The Royal Society Interface 10, 20120847.
662	Moyroud E et al. 2017 Disorder in convergent floral nanostructures enhances signalling to bees. Nature 550, 469–474. (doi:10.1038/nature24285)
663	S. Masrahi Y. 2020 Ecological Adaptations of Urochondra setulosa (Poaceae) against Drought and Salinity. Asian J. of Plant Sciences 19, 443–454. (doi:10.[illegible]
664	S. Masrahi Y. 2020 Ecological Adaptations of Urochondra setulosa (Poaceae) against Drought and Salinity. Asian J. of Plant Sciences 19, 443–454. (doi:10.[illegible]
665	S. Masrahi Y. 2020 Ecological Adaptations of Urochondra setulosa (Poaceae) against Drought and Salinity. Asian J. of Plant Sciences 19, 443–454. (doi:10.[illegible]
666	Gower DJ. 2003 Scale microornamentation of uropeltid snakes. J. Morphol. 258, 249–268. (doi:10.1002/jmor.10147)
667	Gower DJ. 2003 Scale microornamentation of uropeltid snakes. J. Morphol. 258, 249–268. (doi:10.1002/jmor.10147)
668	Gower DJ. 2003 Scale microornamentation of uropeltid snakes. J. Morphol. 258, 249–268. (doi:10.1002/jmor.10147)
669	Gower DJ. 2003 Scale microornamentation of uropeltid snakes. J. Morphol. 258, 249–268. (doi:10.1002/jmor.10147)
670	Gower DJ. 2003 Scale microornamentation of uropeltid snakes. J. Morphol. 258, 249–268. (doi:10.1002/jmor.10147)
671	Gower DJ. 2003 Scale microornamentation of uropeltid snakes. J. Morphol. 258, 249–268. (doi:10.1002/jmor.10147)
672	Antoniou Kourounioti RL et al. 2013 Buckling as an origin of ordered cuticular patterns in flower petals. Journal of The Royal Society Interface 10, 20120847.
673	Moyroud E et al. 2017 Disorder in convergent floral nanostructures enhances signalling to bees. Nature 550, 469–474. (doi:10.1038/nature24285)
674	Keeley BW, Keeley ATH, Houlahan P. 2018 Ridge number in bat ears is related to both guild membership and ear length. PLoS ONE 13, e0200255. (doi:10.
675	Keeley BW, Keeley ATH, Houlahan P. 2018 Ridge number in bat ears is related to both guild membership and ear length. PLoS ONE 13, e0200255. (doi:10.
676	Fang Y, Sun G, Bi Y, Zhi H. 2015 Multiple-dimensional micro/nano structural models for hydrophobicity of butterfly wing surfaces and coupling mechanism. Sci

	A	B	C	D	E	F
677	Terrestrial	Animalia	Chordata	Reptilia	Squamata	Varanidae
678	Terrestrial	Plantae	Angiosperms	Eudicots	Sapindales	Rutaceae
679	Terrestrial	Plantae	Angiosperms	Eudicots	Sapindales	Rutaceae
680	Terrestrial	Plantae	Angiosperms	Eudicots	Sapindales	Rutaceae
681	Terrestrial	Plantae	Angiosperms	Eudicots	Sapindales	Rutaceae
682	Terrestrial	Plantae	Angiosperms	Eudicots	Sapindales	Rutaceae
683	Terrestrial	Plantae	Angiosperms	Eudicots	Sapindales	Rutaceae
684	Terrestrial - extinct	Animalia	Chordata	Sauropsida	Saurischia	Allosauridae
685	Terrestrial - extinct	Animalia	Chordata	Sauropsida	Saurischia	Carcharodontosauridae
686	Terrestrial - extinct	Animalia	Chordata			Carcharodontosauridae
687	Terrestrial - extinct	Animalia	Chordata	Reptilia	Avetheropoda	Neovenatoridae
688	Terrestrial - extinct	Animalia	Chordata	Reptilia	Avetheropoda	Neovenatoridae
689	Terrestrial - extinct	Animalia	Chordata	Sauropsida	Saurischia	Carcharodontosauridae
690	Terrestrial - extinct	Animalia	Chordata	Reptilia	Ornithischia	Hadrosauridae
691	Terrestrial - extinct	Animalia	Chordata	Reptilia	Ornithischia	Hadrosauridae
692	Terrestrial - extinct	Plantae				
693	Terrestrial - extinct	Animalia	Chordata	Sauropsida	Saurischia	Carcharodontosauridae
694	Terrestrial - extinct	Animalia	Chordata	Reptilia	Saurischia	Megalosauridae
695	Terrestrial - extinct	Animalia	Chordata	Mammalia	Multituberculata	
696	Terrestrial - extinct	Animalia	Chordata	Reptilia	Saurischia	Megalosauridae
697	Terrestrial - extinct	Animalia	Chordata	Sauropsida	Saurischia	Carcharodontosauridae
698		Animalia	Chordata	Reptilia	Squamata	Elapidae

	G	H	I	J	K	L	M
677	*Varanus rudicollis*	Tooth	Adult	Infoldings	1000-10000		
678	*Zieria arborescens subsp. ar*	Seed	Seed	Parallel	1-10		
679	*Zieria furfuracea subsp. euth*	Seed	Seed	Parallel	1-10		
680	*Zieria laevigata*	Seed	Seed	Parallel	1-10		
681	*Zieria minutiflora subsp. minu*	Seed	Seed	Parallel	1-10		
682	*Zieria smithii subsp. smithii*	Seed	Seed	Parallel	1-10	10-100	
683	*Zieria veronicea subsp. vero*	Seed	Seed	Parallel	1-10		
684	*Allosaurus fragilis*	Tooth	Adult	Perpendicular	1000-10000	100-1000	Feeding
685	*Carcharodontosaurus from N*	Tooth	Adult	Parallel	10000-10000(	100-1000	Feeding
686	*Carcharodontosaurus sahar*	Tooth	Adult	Perpendicular	10000-10000(	1000-10000	Feeding
687	*Fukuiraptor*	Tooth	Adult	Perpendicular	1000-10000		Feeding
688	*Fukuiraptor*	Tooth			1000-10000		Feeding
689	*Giganotosaurus*	Tooth	Adult	Perpendicular	10000-100000		Feeding
690	*Hadrosaur*	Integument		Radial	1000-10000	1000-10000	Thermoregulation
691	*Hadrosaur*	Integument		Radial	1000-10000	1000-10000	Structural support
692	*Lagenella martini*	Flower		Parallel (proximal) Perpendicular (distal)		0.1-1	
693	*Mapusaurus*	Tooth	Adult	Perpendicular	10000-100000		Feeding
694	*Megalosaurus*	Tooth	Adult	Perpendicular	1000-10000	100-1000	Feeding
695	*Tashtykia primaeva*	Cusp	Adult	Radial		10-100	
696	*Torvosaurus*	Tooth	Adult	Perpendicular	1000-10000		Feeding
697	*Tyrannotitan*	Tooth	Adult	Perpendicular	10000-100000		Feeding
698	*Hydrophis cyanocinctus*	Skin	Adult	Parallel	1000-10000		Friction and Wear reduction

	N
677	Maxwell EE, Caldwell MW, Lamoureux DO, Budney LA. Histology of tooth attachment tissues and plicidentine in Varanus (Reptilia: Squamata), and a discussi
678	Choi B-K, Duretto MF, Hong S-P. 2012 Comparative seed morphology of Boronia and related genera (Boroniinae: rutaceae) and its systematic implications. N
679	Choi B-K, Duretto MF, Hong S-P. 2012 Comparative seed morphology of Boronia and related genera (Boroniinae: rutaceae) and its systematic implications. N
680	Choi B-K, Duretto MF, Hong S-P. 2012 Comparative seed morphology of Boronia and related genera (Boroniinae: rutaceae) and its systematic implications. N
681	Choi B-K, Duretto MF, Hong S-P. 2012 Comparative seed morphology of Boronia and related genera (Boroniinae: rutaceae) and its systematic implications. N
682	Choi B-K, Duretto MF, Hong S-P. 2012 Comparative seed morphology of Boronia and related genera (Boroniinae: rutaceae) and its systematic implications. N
683	Choi B-K, Duretto MF, Hong S-P. 2012 Comparative seed morphology of Boronia and related genera (Boroniinae: rutaceae) and its systematic implications. N
684	Brusatte SL, Benson RBJ, Carr TD, Williamson TE, Sereno PC. 2007 The systematic utility of theropod enamel wrinkles. Journal of Vertebrate Paleontology 2
685	Brusatte SL, Benson RBJ, Carr TD, Williamson TE, Sereno PC. 2007 The systematic utility of theropod enamel wrinkles. Journal of Vertebrate Paleontology 2
686	Brusatte SL, Benson RBJ, Carr TD, Williamson TE, Sereno PC. 2007 The systematic utility of theropod enamel wrinkles. Journal of Vertebrate Paleontology 2
687	Brusatte SL, Benson RBJ, Carr TD, Williamson TE, Sereno PC. 2007 The systematic utility of theropod enamel wrinkles. Journal of Vertebrate Paleontology 2
688	Currie P, Azuma Y. 2005 New specimens, including a growth series, of Fukuiraptor (Dinosauria, Theropoda) from the Lower Cretaceous Kitadani Quarry of Ja
689	Brusatte SL, Benson RBJ, Carr TD, Williamson TE, Sereno PC. 2007 The systematic utility of theropod enamel wrinkles. Journal of Vertebrate Paleontology 2
690	Anderson BG, Lucas SG, Barrick RE, Heckert AB, Basabilvazo GT. 1998 Dinosaur skin impressions and associated skeletal remains from the upper Campania
691	Anderson BG, Lucas SG, Barrick RE, Heckert AB, Basabilvazo GT. 1998 Dinosaur skin impressions and associated skeletal remains from the upper Campania
692	Tekleva MV, Roghi G. 2018 Lagenella martini from the Triassic of Austria – Exine structure and relationships with other striate palynomorphs. Review of Palae
693	Brusatte SL, Benson RBJ, Carr TD, Williamson TE, Sereno PC. 2007 The systematic utility of theropod enamel wrinkles. Journal of Vertebrate Paleontology 2
694	Brusatte SL, Benson RBJ, Carr TD, Williamson TE, Sereno PC. 2007 The systematic utility of theropod enamel wrinkles. Journal of Vertebrate Paleontology 2
695	Averianov AO, Martin T, Lopatin AV, Schultz JA, Schellhorn R, Krasnolutskii S, Skutschas P, Ivantsov S. 2021 Multituberculate mammals from the Middle Jura
696	Brusatte SL, Benson RBJ, Carr TD, Williamson TE, Sereno PC. 2007 The systematic utility of theropod enamel wrinkles. Journal of Vertebrate Paleontology 2
697	Brusatte SL, Benson RBJ, Carr TD, Williamson TE, Sereno PC. 2007 The systematic utility of theropod enamel wrinkles. Journal of Vertebrate Paleontology 2
698	Martinez A, Nguyen D, Basson MS, Medina J, Irschick DJ, Baeckens S. 2021 Quantifying surface topography of biological systems from 3D scans. Methods E
