# Supplementary material for: Groovy and Gnarly: Surface Wrinkles as a Multifunctional Motif for Terrestrial and Marine Environments
Source: Integr Comp Biol. 2022 Jun 8;62(3):749–61. doi: 10.1093/icb/icac079 (PMC9703940; doi:10.1093/icb/icac079)
Supplement: icac079_Supplemental_File [file icac079_supplemental_file.zip › Supplementary information - Fig 2 image permissions and references.pdf]

	A	B	C
1	**Supplementary information - Fig 2 image credits, permissions and references**		
2	**Species**	**Image credits**	**Reference (wrinkle scale, organ and environment)**
3	Window gnat	Martin Cooper from Ipswich, UK, CC BY 2.0 via Wikimedia Commons	Anderson MS, Gaimari SD. 2003 Raman-atomic force microscopy of the ommatidial surfaces of Dipteran compound eyes. Journal of Structural Biology 142, 364–368. (doi:10.1016/S1047-8477(03)00026-1)
4	Hibiscus trionum	Cbaile19, Public domain, via Wikimedia Commons	Chen C, Airoldi CA, Lugo CA, Bay RK, Glover BJ, Crosby AJ. 2021 Flower Inspiration: Broad-Angle Structural Color through Tunable Hierarchical Wrinkles in Thin Film Multilayers. Adv. Funct. Mater. 31, 2006256. (doi:10.1002/adfm.202006256)
5	Isopod	Vittori M. 2021 Structure of a hinge joint with textured sliding surfaces in terrestrial isopods (Crustacea: Isopoda: Oniscidea). Zoological Lett 7, 7. (doi:10.1186/s40851-021-00177-9) - Creative Commons Attribution 4.0 International License	Vittori M. 2021 Structure of a hinge joint with textured sliding surfaces in terrestrial isopods (Crustacea: Isopoda: Oniscidea). Zoological Lett 7, 7. (doi:10.1186/s40851-021-00177-9)
6	Rubber tree	AxelBoldt, Public domain, via Wikimedia Commons	Surapaneni VA, Bold G, Speck T, Thielen M. In press. Spatio-temporal development of cuticular ridges on leaf surfaces of Hevea brasiliensis alters insect attachment. Royal Society Open Science 7, 201319. (doi:10.1098/rsos.201319)
7	Asian blue tick	Alan R Walker, CC BY-SA 3.0 <https://creativecommons.org/licenses/by-sa/3.0>, via Wikimedia Commons	Hackman RH. Structure and function in tick cuticle. Annual review of entomology. 1982 Jan;27(1):75-95 (https://doi.org/10.1146/annurev.en.27.010182.000451)
8	Dock bug	Peter Gabler, Public domain, via Wikimedia Commons	Rebora M, Salerno G, Piersanti S, Gorb EV, Gorb SN. 2021 Attachment devices and the tarsal gland of the bug Coreus marginatus (Hemiptera: Coreidae). Zoomorphology 140, 85–102. (doi:10.1007/s00435-020-00515-z)
9	Trematode	Krailas D, Namchote S, Koonchornboon T, Dechruksa W, Boonmekam D (2014) Trematodes obtained from the thiarid freshwater snail Melanoides tuberculata (Müller, 1774) as vector of human infections in Thailand. Zoosystematics and Evolution 90(1): 57-86. https://doi.org/10.3897/zse.90.7306, CCreative Commons Attribution License (CC BY 4.0)	Tandon V, Maitra SC. Stereocan observations on the surface topography of Gastrothylax crumenifer (Creplin, 1847) Poirier, 1883 and Paramphistomum epiclitum Fischoeder, 1904 (Trematoda: Digenea). J Helminthol. 1981 Sep;55(3):231-7
10	Human	Stephanie Pratt, Public domain, via Wikimedia Commons	Changizi M, Weber R, Kotecha R, Palazzo J. 2011 Are Wet-Induced Wrinkled Fingers Primate Rain Treads? Brain Behav Evol 77, 286–290. (doi:10.1159/000328223)
11	King snake	makindle55, Public domain, via Pixabay	Martinez A, Nguyen D, Basson MS, Medina J, Irschick DJ, Baeckens S. 2021 Quantifying surface topography of biological systems from 3D scans. Methods Ecol Evol 12, 1265–1276. (doi:10.1111/2041-210X.13603)
12	Nepenthes	Alex Lomas, CC BY 2.0, via Wikimedia Commons	Bohn HF, Federle W. 2004 Insect aquaplaning: Nepenthes pitcher plants capture prey with the peristome, a fully wettable water-lubricated anisotropic surface. Proc. Natl. Acad. Sci. U.S.A. 101, 14138–14143. (doi:10.1073/pnas.0405885101)
13	Big brown bat	Ryan Hodnett, CC BY-SA 4.0, via Wikimedia Commons	Keeley BW, Keeley ATH, Houlahan P. 2018 Ridge number in bat ears is related to both guild membership and ear length. PLoS ONE 13, e0200255. (doi:10.1371/journal.pone.0200255)
14	Carcharodontosaurus	Fred Wierum, CC BY-SA 4.0, via Wikimedia Commons	Brusatte SL, Benson RBJ, Carr TD, Williamson TE, Sereno PC. 2007 The systematic utility of theropod enamel wrinkles. Journal of Vertebrate Paleontology 27, 1052–1056. (doi:10.1671/0272-4634(2007)27[1052:TSUOTE]2.0.CO;2)
15	Writh-billed hornbill	Callan Bentley, CC BY-SA 3.0, via Wikimedia Commons	Curio E. 2004 On ornamental maturation of two Philippine hornbill species with a note on physiological colour change. J Ornithol 145. (doi:10.1007/s10336-004-0033-x)
16	Elephant	ajoheho, Public domain, via Pixabay	LILLYWHITE HB, Stein BR. 1987 Surface sculpturing and water retention of elephant skin. Journal of Zoology 211, 727–734. (doi:10.1111/j.1469-7998.1987.tb04483.x)
17	Nile Tilapia	Magdy A. Saleh, CC BY 3.0, via Wikimedia Commons	Fishelson L. 1984 A comparative study of ridge-mazes on surface epithelial cell-membranes of fish scales (Pisces, Teleostei). Zoomorphology 104, 231–238. (doi:10.1007/BF00312036)
18	Mud skipper fish	Totti, CC BY-SA 4.0, via Wikimedia Commons	Hu W, Zhang J, Kang B. 2016 Structure and function of corneal surface of mudskipper fishes. Fish Physiol Biochem 42, 1481–1489. (doi:10.1007/s10695-016-0234-2)
19	Net-winged midge larvae	Liu G-L, Chang H-K, Chuang Y-C, Lin Y-M, Chen P-Y. 2020 Reversible Underwater Adhesion: The Unique C-shaped Suckers of Net-winged Midge Larvae (Blepharicera sp.). Sci Rep 10, 9395. (doi:10.1038/s41598-020-66268-3) - Commons Attribution 4.0 International License	Liu G-L, Chang H-K, Chuang Y-C, Lin Y-M, Chen P-Y. 2020 Reversible Underwater Adhesion: The Unique C-shaped Suckers of Net-winged Midge Larvae (Blepharicera sp.). Sci Rep 10, 9395. (doi:10.1038/s41598-020-66268-3)
20	Mangrove	Dinesh Valke from Thane, India, CC BY-SA 2.0, via Wikimedia Commons	Fu J, Zhang H, Guo Z, Feng D, Thiyagarajan V, Yao H. 2018 Combat biofouling with microscopic ridge-like surface morphology: a bioinspired study. Journal of The Royal Society Interface 15, 20170823. (doi:10.1098/rsif.2017.0823)
21	Pandarus bicolor Leach	Joe Kunkel, some rights reserved (CC-BY-NC), via iNaturalist	Ingram AL, Parker AR. 2006 The functional morphology and attachment mechanism of pandarid adhesion pads (Crustacea: Copepoda: Pandaridae). Zoologischer Anzeiger - A Journal of Comparative Zoology 244, 209–221. (doi:10.1016/j.jcz.2005.11.001)
22	Bottlenose dolphin	Владислав Перић, Public domain, via Wikimedia Commons	Wainwright DK, Fish FE, Ingersoll S, Williams TM, St Leger J, Smits AJ, Lauder GV. 2019 How smooth is a dolphin? The ridged skin of odontocetes. Biol. Lett. 15, 20190103. (doi:10.1098/rsbl.2019.0103)
23	Orca	Mlewan, Public domain, via Wikimedia Commons	Wainwright DK, Fish FE, Ingersoll S, Williams TM, St Leger J, Smits AJ, Lauder GV. 2019 How smooth is a dolphin? The ridged skin of odontocetes. Biol. Lett. 15, 20190103. (doi:10.1098/rsbl.2019.0103)
24	Trilobite	Micha L. Rieser, Attribution, via Wikimedia Commons	Ivantsov AYu, Zakrevskaya MA. 2021 Trilobozoa, Precambrian Tri-Radial Organisms. Paleontol. J. 55, 727–741. (doi:10.1134/S0031030121070066)
25	Wolf fish	Vassil, Public domain, via Wikimedia Commons	Meunier FJ, De Mayrinck D, Brito PM. 2015 Presence of plicidentine in the labial teeth of Hoplias aimara (Erythrinidae; Ostariophysi; Teleostei). Acta Zool 96, 174–180. (doi:10.1111/azo.12065)
26	Giant false limpet	Naturalis Biodiversity Center, Public domain, via Wikimedia Commons	Harley CDG, Denny MW, Mach KJ, Miller LP. 2009 Thermal stress and morphological adaptations in limpets. Functional Ecology 23, 292–301. (doi:10.1111/j.1365-2435.2008.01496.x)
27	Spinosaurus	Levi bernardo, CC BY-SA 3.0, via Wikimedia Commons	McCurry MR, Evans AR, Fitzgerald EMG, McHenry CR, Bevitt J, Pyenson ND. 2019 The repeated evolution of dental apicobasal ridges in aquatic-feeding mammals and reptiles. Biological Journal of the Linnean Society 127, 245–259. (doi:10.1093/biolinnean/blz025)
28	Sponge	Copyright of Stephen B. Church - used with permission	Church SB. 2017 Efficient Ornamentation in Ordovician Anthaspidellid Sponges. PC (doi:10.17161/1808.24771)
29	Humpback whale	8365716, Public domain, via Pixabay	Clapham PJ. Humpback whale: Megaptera novaeangliae. InEncyclopedia of marine mammals 2018 Jan 1 (pp. 489-492). Academic Press.
